# Exceptional evolutionary lability of flower‐like inflorescences (pseudanthia) in Apiaceae subfamily Apioideae

**DOI:** 10.1002/ajb2.1819

**Published:** 2022-03-20

**Authors:** Jakub Baczyński, Hervé Sauquet, Krzysztof Spalik

**Affiliations:** ^1^ Institute of Evolutionary Biology, Faculty of Biology University of Warsaw Biological and Chemical Research Centre Warsaw Poland; ^2^ National Herbarium of New South Wales (NSW) Royal Botanic Gardens and Domain Trust Sydney NSW 2000 Australia; ^3^ Evolution and Ecology Research Centre, School of Biological, Earth and Environmental Sciences University of New South Wales Sydney Australia

**Keywords:** adaptive wandering, BAMM, diversification, macroevolution, MEDUSA, MuHiSSE, pseudocorolla, umbel

## Abstract

**Premise:**

Pseudanthia are widespread and have long been postulated to be a key innovation responsible for some of the angiosperm radiations. The aim of our study was to analyze macroevolutionary patterns of these flower‐like inflorescences and their potential correlation with diversification rates in Apiaceae subfamily Apioideae. In particular, we were interested to investigate evolvability of pseudanthia and evaluate their potential association with changes in the size of floral display.

**Methods:**

The framework for our analyses consisted of a time‐calibrated phylogeny of 1734 representatives of Apioideae and a morphological matrix of inflorescence traits encoded for 847 species. Macroevolutionary patterns in pseudanthia were inferred using Markov models of discrete character evolution and stochastic character mapping, and a principal component analysis was used to visualize correlations in inflorescence architecture. The interdependence between net diversification rates and the occurrence of pseudocorollas was analyzed with trait‐independent and trait‐dependent approaches.

**Results:**

Pseudanthia evolved in 10 major clades of Apioideae with at least 36 independent origins and 46 reversals. The morphospace analysis recovered differences in color and compactness between floral and hyperfloral pseudanthia. A correlation between pseudocorollas and size of inflorescence was also strongly supported. Contrary to our predictions, pseudanthia are not responsible for variation in diversification rates identified in this subfamily.

**Conclusions:**

Our results suggest that pseudocorollas evolve as an answer to the trade‐off between enlargement of floral display and costs associated with production of additional flowers. The high evolvability and architectural differences in apioid pseudanthia may be explained on the basis of adaptive wandering and evolutionary developmental biology.

Pseudanthia are inflorescences that resemble a single flower and usually act as a single pollination unit. Although some are formed only with uniform, undifferentiated flowers (i.e., Mimosoideae), their most widespread and derived types bear pseudocorollas: perianth‐like structures composed of modified peripheral florets in floral pseudocorollas or of extrafloral organs such as bracts, leaves, entire shoots or stipular excrescences in hyperfloral pseudocorollas. Pseudocorollar pseudanthia (hereafter, pseudanthia) arose independently in various lineages of angiosperms, including magnoliids (Tucker, 1981; Todzia, 1988), monocots (Rudall, 2003), and eudicots (Claßen‐Bockhoff, 1990). They are particularly common among campanulid asterids (i.e., Bruniaceae, Caprifoliaceae, Adoxaceae) and sometimes postulated to be a key innovation that led to the spectacular radiation of Asteraceae—one of the largest angiosperm families and having more than 20,000 species (Jeffrey, 2009; Panero and Crozier, 2016).

Contrary to other key floral traits involved in pollination biology such as floral symmetry (Reyes et al., 2016), color (Smith and Goldberg, 2015; Koski, 2020; Skeels et al., 2021), presence of nectar spurs (Fernández‐Mazuecos et al., 2019), or variation in sexual systems (Goldberg et al., 2017), the evolutionary patterns of flower‐like inflorescences and their potential link with diversification has not yet been addressed using phylogenetic comparative methods. Our knowledge about ecological importance of pseudanthia is also impaired due to their frequent association with highly generalized pollination systems, which are notoriously difficult to study. Few surveys indicated that perianth‐like organs give a strong selective advantage (Sun et al., 2008) and increase seed set (Stuessy et al., 1986), but the fitness gains resulting from such architecture may be dependent on the overall size of inflorescence (Andersson, 1996) and available pollinators niches (Andersson, 1999, 2008; Celedón‐Neghme et al., 2007; Nielsen et al., 2002).

Apioid umbellifers (Apiaceae subfamily Apioideae) with ca. 400 genera and 3000 species constitute the core clade of the campanulid order Apiales and include economically important crops (carrot, parsley) and spices (cumin, coriander, caraway). Their flowers are usually organized into a distinctive, modular inflorescence (Figure 1A), known as compound umbel (umbel composed of umbellets). Its ostensible uniformity in fact hides large variation in spatial and temporal organization, size gradient, and floral sex distribution (Reuther and Claßen‐Bockhoff, 2010). Apioids are also the only group of angiosperms where pseudanthia with both floral (Figure 1B) and hyperfloral (Figure 1C) pseudocorollas can be found in closely related plants (Claßen‐Bockhoff, 1990). These two types of perianth‐like organs occur in combination with differences in shape (flat‐surfaced vs. spherical), color (white, yellow, or purple), and size of floral display. The array of different forms of flower‐like inflorescences make Apioideae an interesting model for investigation of their evolutionary significance.

**Figure 1:**
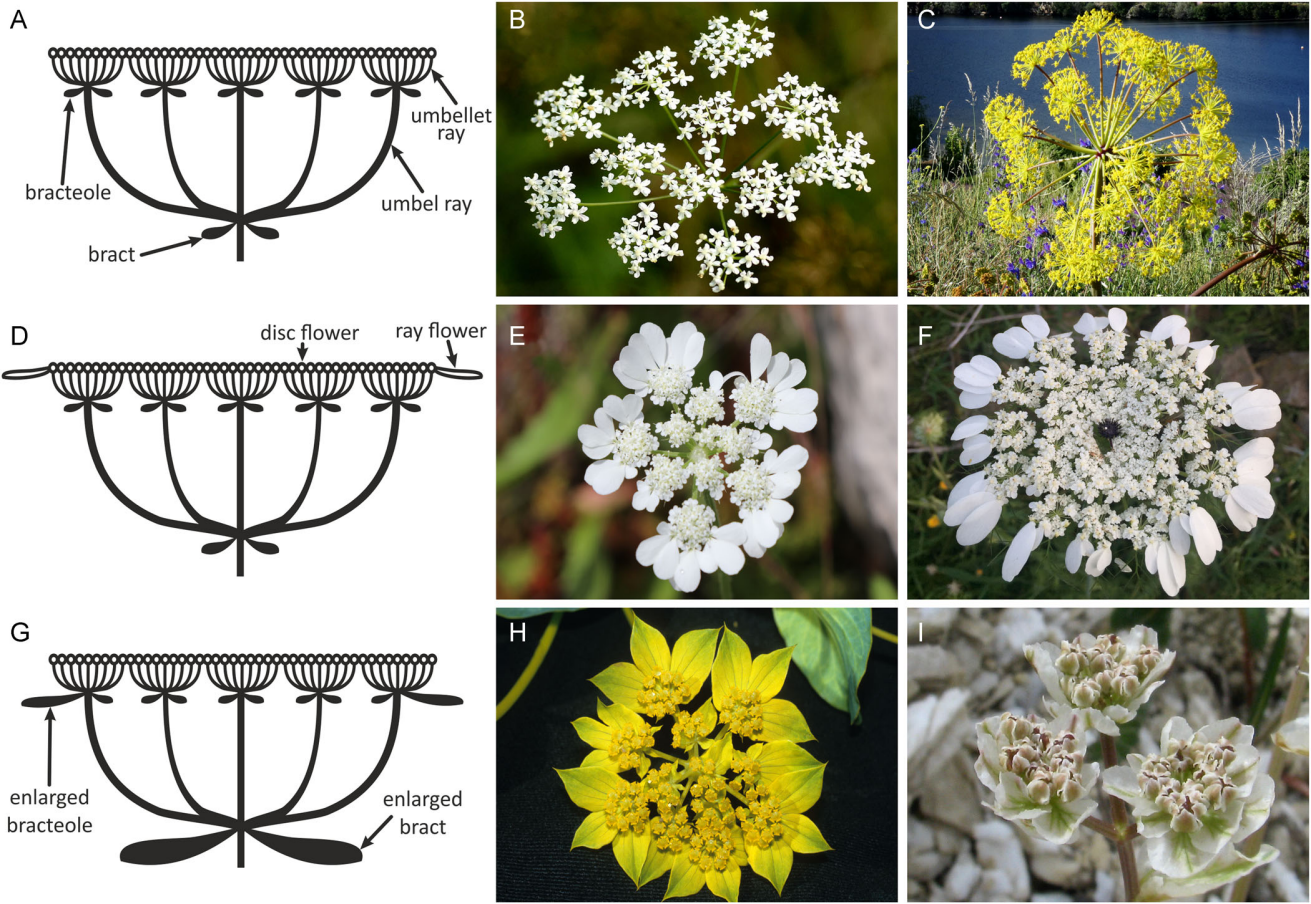
Diversity of inflorescences in Apiaceae subfamily Apioideae. (A–C) Compound umbel without a pseudocorolla: (B) Pimpinella saxifraga L. and (C) Ferula communis L. (D–F) Pseudanthium with a pseudocorolla formed by enlarged peripheral florets: (E) Tordylium brachytaenium Boiss. & Heldr. and (F) Artedia squamata L. (G–I) Pseudanthium with a hyperfloral pseudocorolla formed by involucellar and/or involucral bracts: (H) Bupleurum sp. L. and (I) Hymenidium nanum (Rupr.) Pimenov & Kljuykov. Photo credits: (E, F) Jakub Baczyński, Regine Classen‐Bockhoff (H) or adapted from Wikimedia Commons after Udo Schmidt (B), Miguel Angel Masegosa Martínez (C), and Alexey Yakovlev (I)

The exact number and phylogenetic placement of independent origins of pseudanthia in Apioideae has not been reconstructed so far. Fortunately, in the last 20 years much effort has been made to establish a well‐supported phylogeny for this subfamily (Downie et al., 1996, 2010; Downie and Katz‐Downie, 1996; Katz‐Downie et al., 1999; Ajani et al., 2008; Zhou et al., 2008; Magee et al., 2010; Banasiak et al., 2013; Wen et al., 2020; Clarkson et al., 2021) that can be used as a framework for such inference. Apioideae are also an interesting target for investigation of diversification patterns associated with pseudanthia because the distribution of species within this group is highly uneven among the major clades—often formally recognized as tribes—with some small (e.g., Coriandreae) or monospecific (e.g., Erigenieae) and others (particularly, Selineae and Scandiceae) comprising a few hundred species each (Plunkett et al., 2018). This variation, coupled with a comprehensively sampled molecular phylogeny and multiple independent origins of pseudanthia allow to address the limitations of existing trait‐dependent diversification models (Maddison and FitzJohn, 2015; Gamisch, 2016; Rabosky and Goldberg, 2015; May and Moore, 2016; Kodandaramaiah and Murali, 2018; Simpson et al., 2018).

In this study, we investigated evolutionary patterns of pseudanthia using Apiaceae subfamily Apioideae as a model. By applying a robust macroevolutionary toolkit (ancestral state reconstruction under Markov models and stochastic mapping, morphospace analysis, trait‐dependent and trait‐independent diversification models) over the most species‐rich apioid phylogeny and the largest morphological matrix of inflorescence‐derived traits encoded for this group, we aimed to answer the following questions: (1) What are the macroevolutionary dynamics of pseudanthia? (2) Is the evolution of pseudanthia correlated with changes in size of floral display? (3) Are there any morphological correlations in different pseudanthial architectures? (4) What are the diversification patterns of Apioideae and can they be explained by the evolution of pseudocorollas?

## MATERIALS AND METHODS

### Taxon sampling and molecular data set

Sequences of nuclear nrDNA ITS and ETS and plastid *rpoC1*, *rps16*, and *rpl16* introns for the species representing Apiaceae subfamily Apioideae were downloaded from NCBI using MatPhylobi, a bioinformatic tool created by Hanna Kranas and Łukasz Banasiak (https://github.com/hansiu/MatPhylobi). The software automatically joins conspecific ITS1 and ITS2 sequences that were frequently obtained and published separately because of historical limitations of sequencing methods. It also allows for specification of a blacklist including accessions previously identified as erroneous or dubious (e.g., obtained from misidentified specimens or resulting from laboratory contamination with other taxa) by various researchers. The tree was rooted with *Sanicula epipactis* E.H.L. Krause, a representative of subfamily Saniculoideae that constitutes the sister group of apioids (Valiejo‐Roman et al., 2002; Calviño and Downie, 2007; Calviño et al., 2008; Clarkson et al., 2021).

We filtered the molecular data set retaining only those species for which the ITS sequence was available. If the sequence was partial (e.g., contained only ITS1 or ITS2), we included the taxon only if at least one plastid marker was additionally sampled. Resulting matrices were aligned separately using the G‐INS‐I algorithm implemented in MAFFT v.7.271 (Katoh and Standley, 2013) and automatically trimmed with trimAl v.1.2rev59 (Capella‐Gutiérrez et al., 2009) with the –automated1 option. The resulting matrix comprised 4059 nucleotide positions and 1734 species including representatives of all major lineages (tribes) of Apioideae. The coverage of markers was 100% for nrDNA ITS, 19% for nrDNA ETS, 15% for both *rpoC1* and *rpl16* introns, and 46% for *rps16* intron. The inclusion of incompletely sampled markers in molecular data sets has proven to be beneficial by rescuing analyses from long‐branch attraction and improving phylogenetic resolution (Wiens, 2003, 2005; Wiens and Tiu, 2012; Jiang et al., 2014). Although some authors suggested that missing data can influence branch length estimates (Lemmon et al., 2009; Brown et al., 2010), the bias introduced even with highly incomplete molecular matrices was proven insignificant when available sites retain consistent information about branch lengths (Wiens and Morrill, 2011; Roure et al., 2013; Jiang et al., 2014).

The list of accessions is given in Appendix S1.

### Morphological data set

A data set of five quantitative (continuous) and six qualitative (discrete) floral and inflorescence traits was compiled for 847 species of apioid umbellifers (Table 1). The choice of species was primarily guided by the availability of molecular data and designed to account for both phylogenetic and morphological diversity of Apioideae. Character typology and scoring were mostly based on floristic and taxonomic accounts that, if possible, were verified with online photographs of herbarium specimens and personal observations (the list of main sources used for each species is available in Appendix S2). Because morphological descriptions are inevitably biased by individual perception of (frequently continuous) morphological variation, we applied a strict coding regimen for creating our typology. For example, flowers were scored as “white” only when described as such without any adjectives indicating hues; the latter were assigned to respective states: “yellow/yellowish” (e.g., “yellowish white”, “green‐yellow”, “creamy”) or “purple/purplish” (e.g., “purplish tinged”, “purplish white”). The discretization of other traits—sepals and involucral and involucellar bracts—was consistent across various sources, and we applied it without further adjustments. Although frequently provided in descriptions, the degree of ray flowers’ pronouncement (e.g., “outer petals weakly/strongly radiant”) is highly subjective, especially when intraspecific variation is taken into account (Sheppard, 1991). Therefore, in our analyses, we scored only the presence of floral pseudocorollas but not their size.

**Table 1:** Morphological characters used to score inflorescence traits

Trait name	Coding scheme
Minimum number of umbellets	Primary continuous
Maximum number of umbellets	Primary continuous
Minimum number of flowers per umbellet	Primary continuous
Maximum number of flowers per umbellet	Primary continuous
Average umbel size	Secondary continuous
Outer and inner rays	0, unequal; 1, subequal to equal
Flower color	0, white; 1, yellowish to yellow; 2, purplish to purple
Involucral bracts	0, absent; 1, single; 2, numerous; 3, showy (hyperfloral pseudocorolla)
Involucellar bracts	0, absent; 1, single; 2, numerous; 3, showy (hyperfloral pseudocorolla)
Sepals	0, absent; 1, minute; 2, conspicuous; 3, asymmetric
Ray flowers	0, absent; 1, present (floral pseudocorolla)

The scoring scheme allowed for missing data (when the description was incomplete or imprecise) and polymorphisms (indicating variable state of a character in a single species). Average umbel size, a secondary continuous character used in some analyses, was calculated as [(Minimum number of umbellets × Minimum number of flowers) + (Maximum number of umbellets × Maximum number of flowers)]/2. This approach should give a good approximation of inflorescence size for apioids, as their flowers are relatively uniform in size. (Although precise measurements are hardly available, individual florets rarely exceed 4 millimeters in diameter, e.g., Chen and Wang, 2001; Van Wyk et al., 2010; Thakur et al., 2020).

Although this typology may easily be applied to the vast majority of apioids, the taxa with strongly reduced compound umbels such as *Lilaeopsis* (Affolter, 1985) pose a challenge to the formulation of primary homology statements. In this study, we assumed that a derived simple umbel is an equivalent of a single ray from the compound umbel and its subtending organs are involucellar rather than involucral bracts.

### Phylogeny reconstruction and dating

Phylogenetic inference was conducted with a maximum likelihood (ML) approach implemented in RAxML 8.2.4 with a GTR + G substitution model recommended by the author of the software (Stamatakis, 2014). The data set was divided into two partitions (nuclear and plastid markers) following the results of PartitionFinder 2.1.1 (Lanfear et al., 2012) analyses with the options branch lengths linked and AIC_c_. Branch support was evaluated with 1000 rapid bootstrap replicates. Shimodaira–Hasegawa‐like approximate‐likelihood ratio test (SH‐like aLRT) analysis was performed to obtain an alternative measure of branch support, as pseudoreplication‐based methods may fail to recover particular nodes when data sets are highly incomplete. Prior to all subsequent analyses, the outgroup was removed from the tree.

The macrofossil record of Apioideae is extremely sparse (Manchester et al., 2015), while palynomorphs used to calibrate previous molecular dating studies were usually assigned to particular nodes at face value (Banasiak et al., 2013; Nicolas and Plunkett, 2014; Wen et al., 2020). More detailed analyses of pollen grains of Apiales (Baczyński et al., 2021) allowed for a choice of more reliable calibration points consisting of four minimum age constraints on internal nodes. Detailed data for the age and node assignment of the fossils are provided in Appendix S3, following the format used by Ramírez‐Barahona et al. (2020) to justify angiosperm fossil calibrations. Divergence times were estimated using penalized likelihood (Sanderson, 2002) as implemented in TreePL (Smith and O'Meara, 2012) with the root (crown node) of Apioideae constrained between 42 and 72 Ma in accordance to results of Nicolas and Plunkett (2014) and Wen et al. (2020). The priming analysis and the optimization of smoothing factor were performed before running the final analysis resulting in the following set of parameters: opt = 4, moredetail, optad = 4, moredetailad, optcvad = 4, smooth = 0.1. We refrained from using Bayesian Markov chain Monte Carlo (MCMC) dating because of the limitations on the computational power required to reach convergence due to the large number of taxa included in our data set.

### Ancestral state reconstruction and correlation analyses

We reconstructed ancestral states using Markov models and a maximum likelihood (ML) approach as implemented in the corHMM 2.4 package in R (Boyko and Beaulieu, 2020). The analyses were performed separately for the type of pseudocorolla and the average inflorescence size, then on both traits combined to test for correlated evolution between these two characters. For each analysis, the chronogram obtained with TreePL was pruned to match the morphological data set: 847 taxa for pseudocorollas and 525 taxa for inflorescence size and the two traits combined. Types of pseudocorollas were encoded as a three‐state character (0, absent; 1, floral; 2, hyperfloral). As the analyses required qualitative data, inflorescence size was discretized based on tertiles of its distribution resulting in three states: small (<82 flowers), medium (83–214 flowers), and large (>214 flowers). To compute the total number of state changes for the type of pseudocorolla, we used a stochastic mapping approach with the makeSimmap function (500 simulations across the tree) implemented in corHMM package. To account for potential impact of different coding scheme for recovered instances of pseudanthia origins, the analysis was replicated by combining floral and hyperfloral pseudocorollas as a single state (0, absent; 1, present; see Appendix S4).

To check the assumptions on directions of evolutionary transitions in inflorescence size and type of pseudocorolla, we compared ordered and unordered versions of all models for both these traits (Appendix S5A). Our preliminary analyses indicated that different types of pseudocorollas cannot directly evolve one into another but have to pass through the state “absent”, suggesting that this trait is ordered. Inflorescence size was also reconstructed as an ordered character with transitions between small and large umbels occurring via the “medium” state.

We further analyzed the type of pseudocorollas and inflorescence size jointly to test for correlated evolution between these two characters. We applied maximum likelihood tests derived from Pagel's (1994) method. The combination of our two three‐state characters resulted in a transition rate matrix of nine by nine possible state combinations, with 72 transition rate parameters. However, ordering both characters allowed for matrix to be reduced to 24 parameters in the most complex model. By editing the combination of ordered transition matrices for both traits, we fitted two variants of SYM and ARD models for the combination of the two traits: correlated (the state of one character affects the relative transition rates of a second) and uncorrelated (states of characters change independently from each other). The schematic diagrams of all transition matrices are listed in Appendix S5A.

### Principal component analysis

A principal component analysis was performed to investigate correlations in inflorescence architecture, which could have evolved in response to certain selective drives (e.g., adaptive wandering). Specifically, we used a mixed PCA approach implemented in R package PCAmixdata 3.1 (Chavent et al., 2014). This method handles a combination of both qualitative and quantitative data using generalized singular value decomposition but, unfortunately, does not allow for phylogenetic correction (Nye, 2011; Polly et al., 2013). The coding scheme followed Table 1, resulting in 23 distinct eigenvectors corresponding to either state (for qualitative data) or trait (for primary quantitative data). As no form of multidimensional scaling can tackle polymorphic qualitative characters, we decided to use the approach presented in Chartier et al. (2017). Briefly, this approach involved repeated random solving of polymorphisms (choosing one for each case) and re‐running the analysis to ascertain that initial differences do not affect data visualization. Additionally, to ensure that our data set has a structure that creates at least one distinct eigenvalue, we compared values of *ψ* and *φ* statistics on 100 replicates of unrandomized and randomized matrices (values for each trait are shuffled independently), following Björklund (2019). The scree plots for eigenvalues were inspected for both variants to determine how many principal components to interpret. The results were visualized with ggplot2 3.3.2 (Wickham, 2016).

To explore the influence of different ordination techniques on the reconstruction of morphospace, we applied NMDS (nonmetric multidimensional scaling) based on Gower distances (Appendix S6A), as implemented in the R package vegan 2.5‐6 (Oksanen et al., 2013). The polymorphisms were solved 100 times, according to the method described above. The homogeneity of dispersion for different pseudanthial architectures (absent, floral and hyperfloral) was tested using the betadisper function (Appendix S6B), which performs a multivariate analogue of Levene's test for homogeneity of variances. To check for significant differences of the groups' centroid/dispersion, we performed the permutational multivariate analysis of variance (PERMANOVA), available under the adonis function.

### Trait‐independent diversification analyses

Trait‐independent diversification rate shift analyses were performed on the TreePL chronogram with a stepwise AIC‐based approach as implemented in MEDUSA (Alfaro et al., 2009) and with Bayesian analysis of macroevolutionary mixtures (BAMM: Rabosky et al., 2014). Despite concerns surrounding both methods (May and Moore, 2016; Moore et al., 2016; Rabosky et al., 2017; Rabosky, 2018) and the use of dated molecular phylogenies to estimate parameters of generalized birth–death models in general (Louca and Pennell, 2020), we believe that this type of inference will at least allow us to test for net diversification rates heterogeneity within Apioideae. MEDUSA analyses were conducted in R with package geiger 2.07 using three available methods: pure Yule, birth–death, and a mix of these processes. BAMM was run over 100 million generations with initial parameters estimated with R package BAMMtools 2.1.7. The analysis was repeated with three different Poisson rate priors (0.05, 0.1, 0.2) and expected number of shifts (5, 10, 20) to ensure that the results are not entirely conditioned by these settings (Moore et al., 2016). Each variant reached convergence with estimated samples sizes >200 after initial 10% burn‐in. Because MEDUSA and BAMM both assume complete sampling within analyzed clades, we provided the information about species richness by evenly distributing missing species among sampled representatives of each genus (Appendix S7A, B). The information about generic level diversity of apioids (Appendix S7D) was taken from Plants of the World Online (http://www.plantsoftheworldonline.org/) and—whenever necessary—corrected following the most recent taxonomic revisions (Spalik et al., 2004; Sun et al., 2004; Ronse et al., 2010; Banasiak et al., 2016). To investigate how this approach may influence the estimates of diversification rates, we also replicated the BAMM and MEDUSA analyses without specifying the sampling fraction.

### Trait‐dependent diversification analyses

To address the influence of different types of pseudanthial pseudocorollas on diversification rates within Apioideae, we used one of the most recent members of the ever‐expanding family of state speciation and extinction (SSE) models. MuHiSSE (Nakov et al., 2019), implemented in the R package hisse 1.9.10, applies the framework developed within HiSSE (Beaulieu and O'Meara, 2016; Caetano et al., 2018) to MuSSE (Multiple State Speciation and Extinction; FitzJohn, 2012) to account for the potential impact of additional factors or traits on rate heterogeneity within the tree and to deal with multiple state/traits at the same time. While earlier iterations of SSE models independently estimate speciation (*λ*), extinction (*μ*), and transition rate parameters (*q*), MuHISSE fits derived measures of diversification defined as turnover (*τ* = *λ* + *μ*; rate of birth or death per unit of time) and extinction fraction (ε = *μ*/*λ*; ratio of death and birth events per unit of time).

**Table 2:** MuHiSSE models fitted for different types of pseudocorollas in trait‐dependent diversification analyses. Abbreviations “Div. pars” and “Trans. pars” denote the numbers of diversification rate parameters and transition rate parameters, respectively. The best‐fitting model as chosen by AIC_c_ is marked in boldface. See Appendix S4 for graphical illustrations of different models used in this analysis

Model	Hidden traits	ln L	Div. pars	Trans. pars	AIC_c_
MuSSE equivalent (constrained ε)	0	–3159.9	4	4	6335.9
MuSSE equivalent	0	–3156.5	6	4	7704.4
MuCID‐3 (constrained ε)	2	–2649.8	4	18	5344.8
**MuCID‐3**	**2**	**–2628.1**	**6**	**18**	**5305.7**
MuHiSSE absent (constrained ε)	1	–2669.5	5	6	5361.2
MuHiSSE absent	1	–2667.4	8	6	5363.3
MuHiSSE floral (constrained ε)	1	–2682.4	5	6	5387.2
MuHiSSE floral	1	–2707.4	8	6	5443.3
MuHiSSE hyperfloral (constrained ε)	1	–2670.0	5	6	5421.9
MuHiSSE hyperfloral	1	–2689.8	8	6	5407.5
MuHiSSE all states (constrained ε)	1	–2647.8	7	10	5330.3
MuHiSSE all states	1	–2660.5	12	10	5366.2

Using maximum likelihood, we fitted a set of 12 MuHiSSE models with increasing complexity (Table 3, Appendix S5B) to a data set comprising the morphological matrix with 847 taxa and the chronogram pruned to match it. Similar to the ancestral state reconstruction analyses, pseudocorollas were treated as an ordered character. To take incomplete taxonomic sampling into account, we specified the sampling fraction for each of the three states (probability of an extant species in a particular state being included in the phylogeny). While assessing these parameters, we used an indirect approach because the descriptions of many species were not easily available or lacked information on inflorescence architecture. First, for each sampled polymorphic genus (with more than one character‐state present among its representatives), we calculated the proportions of species with particular character states using the available data and assumed that these proportions applied to the entire genus. The number of species in each genus was taken from the Plants of the World Online (POWO) database. For example, *Chaerophyllum* is represented by 33 species in our data set, but 70 species in total are currently accepted. Of the species sampled in our data set, three (9.1%) have floral pseudocorollas, seven (21.2%) have hyperfloral pseudocorollas, while in 23 species (69.7%), pseudocorollas are absent. In polyphyletic genera (such as *Heracleum*), unsampled species were presumed to be distributed evenly among different clades. The genera that were monomorphic based on sampled species were also assumed to be monomorphic across all species.

**Table 3:** CorHMM models fitted for different types of pseudanthial pseudocorollas, size and both traits combined. The best models, chosen with AIC_c_, are marked in boldface

Trait	Rate parameters	ln L	No. of parameters	AIC_c_
Pseudocorollas	Ordered ER	–332.7	1	667.5
Pseudocorollas	Ordered SYM	–332.7	2	669.4
**Pseudocorollas**	**Ordered ARD**	**–287.3**	**4**	**582.7**
Pseudocorollas	ER	–337.1	1	676.2
Pseudocorollas	SYM	–332.7	3	671.4
Pseudocorollas	ARD	–287.3	6	586.8
Size	Ordered ER	–549.2	1	1100.4
Size	Ordered SYM	–545.6	2	1097.2
**Size**	**Ordered ARD**	**–542.8**	**4**	**1093.6**
Size	ER	–575.2	1	1152.5
Size	SYM	–545.6	3	1097.2
Size	ARD	–542.8	6	1097.7
Pseudocorollas + size	Ordered SYM, uncorrelated	–771.3	4	1575.6
Pseudocorollas + size	Ordered ARD, uncorrelated	–742.4	8	1537.5
Pseudocorollas + size	Ordered SYM, correlated	–758.8	12	1595.1
**Pseudocorollas** + **size**	**Ordered ARD, correlated**	**–726.3**	**24**	**1530.0**

All models were tested in two variants—with a single extinction fraction (*ε*) across all states or with varying extinction fraction—and subsequently evaluated using AIC_c_. First, we fitted MuSSE‐like models (without hidden traits) assuming that changes in diversification rates are driven only by the observed character. MuHiSSE (with hidden traits) models were then optimized with hidden state associated only with one of the observed states, i.e., separately for the absence of pseudocorollas, for floral pseudocorollas, and for hyperfloral pseudocorollas, and then with all these three states at once. MuCID (multistate character‐independent) models with diversification parameters unlinked to observed traits were used as a null hypothesis. Specifically, we optimized MuCID‐3, a “fair competitor” for MuSSE models sharing the same number of diversification parameters (Beaulieu and O'Meara, 2016). Graphs visualizing all used rate matrices are given in Appendix S5B.

Furthermore, we tested for trait‐dependent diversification with floral and hyperfloral pseudanthia treated as a single state to elucidate the effect of sheer pseudocorolla presence (without distinguishing between its different types) on speciation and extinction rates. The fitted models were equivalent to those used in the multistate approach, although redesigned to account for a binary character (BiSSE‐like, CID‐2, CID‐4, and HiSSE).

## RESULTS

### Phylogeny and divergence times

The results of our phylogenetic analyses (Appendix S8) are consistent with published studies (Ajani et al., 2008; Zhou et al., 2008; Downie et al., 2010; Banasiak et al., 2013), including recent phylotranscryptomic analyses of Apioideae or Apiales as a whole (Wen et al., 2020; Clarkson et al., 2021). Our reconstructed topology indicates that Choritenieae+Lichtensteinieae constitute the sister group to the rest of the subfamily, followed by other African apioids (Annesorhizeae, Heteromorpheae, and Chamaesieae), early‐diverging apioids (Bupleureae, Pleurospermeae, Komarovieae, Oenantheae) and two large sister clades of core Apioideae—‘Scandiceae and relatives’ and the apioid superclade. The backbone relationships and almost all tribes/groups were highly supported by bootstrap (>70%) and/or SH‐like aLRT (>95%). The results of our divergence time analysis show that all major lineages originated and diversified about 10 Mya earlier than the estimates of Banasiak et al. (2013) and Wen et al. (2020). As this study was not designed to directly address the phylogeny or timing of major divergence events in Apioideae, these results will not be further discussed here.

### Ancestral state reconstruction

**Figure 2:**
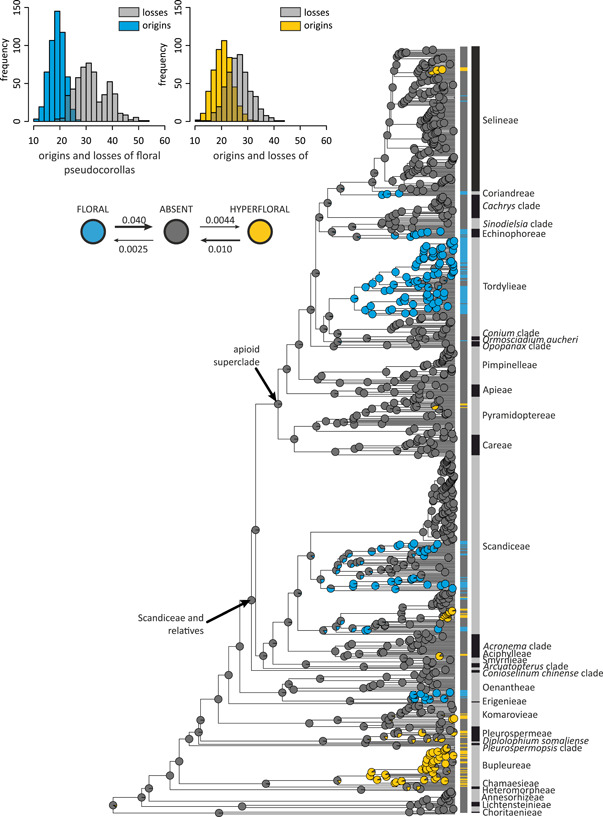
SIMMAP reconstruction for pseudocorolla type in Apiaceae subfamily Apioideae averaged across 500 stochastic character‐mapped trees. Pie charts at each node represent the probabilities for each state (gray: absent, blue: floral pseudocorollas, yellow: hyperfloral pseudocorollas). Boxes on the right side of the tree indicate tip states. Histograms represent the inferred number of origins and reversals for floral and hyperfloral pseudocorollas. The plot below the histograms shows a visualization of the transition rate matrix resolved by corHMM, with arrow widths proportional to transition rates

The evolution of pseudocorollas is best explained by the ordered ARD model (ΔAIC_c_ < 2; Table 3; Appendix S5A) with rates of gains being more than one order of magnitude lower (0.003 and 0.004 for floral and hyperfloral pseudocorollas, respectively) than reversals (0.040 and 0.010, respectively). Unordered versions of investigated models estimated the rate of direct changes between floral and hyperfloral pseudocorollas as zero, effectively reconstructing identical patterns to their ordered equivalent. Pseudanthia were ancestrally absent in Apioideae and results of stochastic mapping (Figure 2) recovered between 93 and 144 state changes for this character (with 95% CI for number of transitions being 115–117). Floral pseudocorollas were acquired less frequently (95% CI of 19–20) than hyperfloral ones (28–29) with a very similar number of losses recorded for both these architectures (32–33 vs. 34–35 reversals on average). The lowest numbers of origins and losses of pseudanthia were reconstructed as 36 and 64, respectively. When treated as a single character (Appendix S4D), pseudocorollas were estimated to have originated between 34 and 66 (95% CI of 52–54) with 40 to 113 subsequent losses (95% CI of 62–65). In this scenario, the ARD model was also chosen as the best fit.

Hyperfloral pseudocorollas are common in early‐branching apioids and evolved in Komarovieae, Bupleureae, the *Pleurospermopsis* clade, and Pleurospermeae. In later‐diverging apioids, this architecture is rare and dispersed among several distantly related genera: *Chaerophyllum* (Scandiceae), *Hyalolaena* (Pyramidoptereae), *Vesper* (Selineae), and a part of the polyphyletic genus *Hymenidium* (Figure 2; Appendices S4D, S5C). In contrast, floral pseudocorollas appeared relatively late in Apioideae and evolved independently in Oenantheae and Scandiceae and at the base of a large clade composed of Echinophoreae, the *Sinodielsia* clade, the *Cachrys* clade, Tordylieae, Coriandreae, and Selineae.

In contrast to pseudocorollas, the ancestral state for average inflorescence size was ambiguous not only at the root, but also along the backbone of apioid phylogeny (with all three size classes being almost equally likely; Appendix S5D). Large umbels are rather uncommon in early‐diverging apioids, appearing more frequently in Scandiceae and relatives (mostly in Ferulinae and Daucinae) and the apioid superclade (especially, Selineae and Tordylieae). The best‐fitting model was the ordered ARD model (ΔAIC_c_ < 2, Table 3) with transition rates from small to medium (0.21), and medium to large inflorescences (0.010) being higher than changes in the opposite direction (respectively 0.16 and 0.078). As for the pseudocorolla character, unordered versions of models of inflorescence size evolution suggested that direct transitions between small and large inflorescences are unlikely, with the estimated rates close to zero. This result supports the notion that umbel size in Apioideae changes gradually rather than abruptly in the course of evolution.

**Figure 3:**
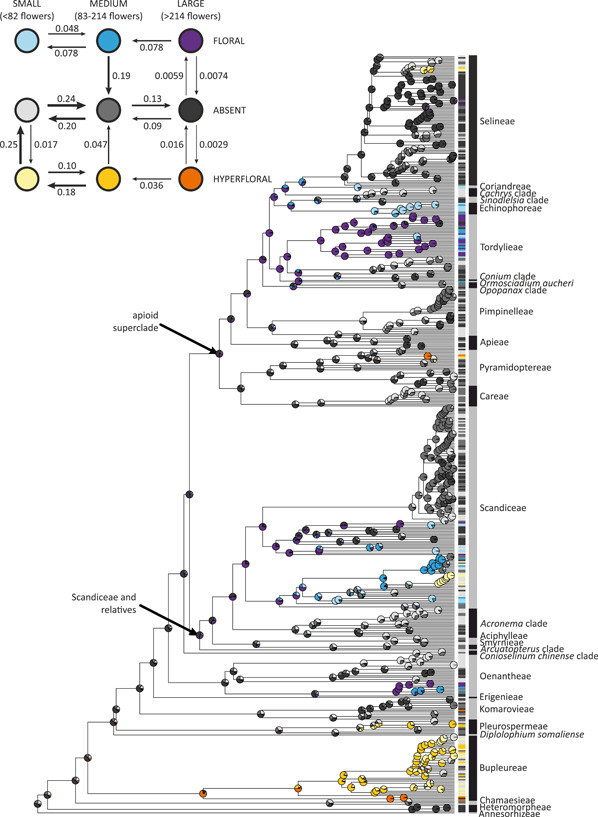
Maximum likelihood reconstruction of ancestral states for the correlated model of combined inflorescence size and type of pseudocorolla. The plot in the top‐left corner shows a visualization of the transition rate matrix with arrow widths proportional to transition rates. Transitions rates estimated as zero are not shown. Boxes on the right side of the tree indicate tip states. For ancestral state reconstructions of pseudocorollas and inflorescence size treated separately, see Appendix S5C,D

The analysis of combined umbel size and pseudocorolla type (Figure 3) strongly indicated that the evolution of these traits is correlated and best explained by an ARD model (ΔAIC_c_ < 2, Table 3). The most likely ancestral state for Apioideae was reconstructed as large, non‐pseudanthial inflorescence. While hyperfloral pseudocorollas may originate in either small or large umbels, floral ones appear only in the latter. In all aforementioned evolutionary scenarios, the losses are favored over gains, especially for hyperfloral pseudocorollas (0.25 over 0.017 for small and 0.016 over 0.0029 for large inflorescences). The transition rates from medium‐sized inflorescences to pseudanthia (floral and hyperfloral) are estimated as zero, and a similar pattern was found for transitions between small‐sized inflorescences and floral pseudanthia (in both directions). The increase of inflorescence size is more common than the decrease in non‐pseudanthial lineages, whereas in pseudanthial lineages the trend is reversed.

### Principal component analyses

**Figure 4:**
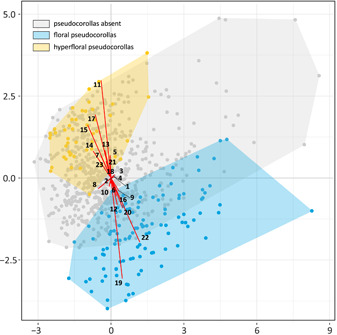
Principal component analysis (PCA) for the mix of qualitative and quantitative inflorescence traits. The plot shows the first two principal components, which together explain 24% of the variance. Each point represents a species with colors indicating presence of floral (blue) or hyperfloral (yellow) pseudocorollas. Convex hulls represent parts of the morphospace occupied by species with each of these traits. Numbers at tips of eigenvectors indicate associated trait/state: 1, minimum number of rays; 2, maximum number of rays; 3, minimum number of flowers per umbellet; 4, maximum number of flowers per umbellet; 5, outer and inner rays equal to subequal, 6, outer and inner rays unequal; 7, flowers yellow to yellowish; 8, flowers purple to purplish; 9, flowers white; 10, single involucral bract; 11, involucral bracts showy; 12, involucral bracts numerous; 13, involucral bracts absent; 14, single involucellar bract; 15, involucellar bracts showy; 16, involucellar bracts numerous; 17, involucellar bracts absent; 18, sepals minute; 19, sepals asymmetric; 20, sepals conspicuous; 21, sepals absent; 22, ray flowers present; 23, ray flowers absent

In the principal component analyses, the *ψ* and *φ* statistics for randomized data sets were lower than unrandomized ones across all replicates indicating that the original data set has a biologically meaningful structure. The first two principal components (PCs) explain 24% of the variance within the data set (Figure 4); therefore, we also checked the combinations of the first five PCs that together accounted for over 50% of the variance (Appendix S9). All analyzed PCs were situated above the elbow of scree plot and had higher eigenvalues than their randomized equivalents.

In the morphospace defined by first two PCs, species with floral pseudocorollas do not overlap with those having hyperfloral pseudocorollas; however, both groups partly overlap with those without pseudocorollas (Figure 4). The presence of ray flowers is correlated with asymmetric sepals and, to a lesser extent, unequal outer and inner rays (i.e., outer rays longer than inner ones resulting in a flat inflorescence surface), white petals, and the presence of involucral and involucellar bracts. Hyperfloral pseudocorollas are not strongly linked to any characters analyzed although species with this trait generally occupy a part of the morphospace defined by yellow petals and subequal/equal outer and inner umbel rays. Parameters describing inflorescence size (the numbers of umbel rays and of flowers) very weakly contribute to the first five principal components and are unable to explain differences between the floral and hyperfloral pseudanthia.

The results of betadisper indicate that the variance between groups is heterogenous across all 100 replicates and thus violates one of the assumptions of PERMANOVA. However, as our design is unbalanced in a way that larger groups have larger dispersion, we expect conservative behavior of PERMANOVA that makes type I error less likely to occur (Anderson and Walsh, 2013). Therefore, we report that the centroid of species with floral pseudocorollas was significantly different from centroids of remaining groups (Appendix S6B). The stress value in each replicate of NMDS analysis was below 0.2, indicating that the produced plot (Appendix S6A) provides good representation of reduced dimensions.

### Diversification rate analyses

**Figure 5:**
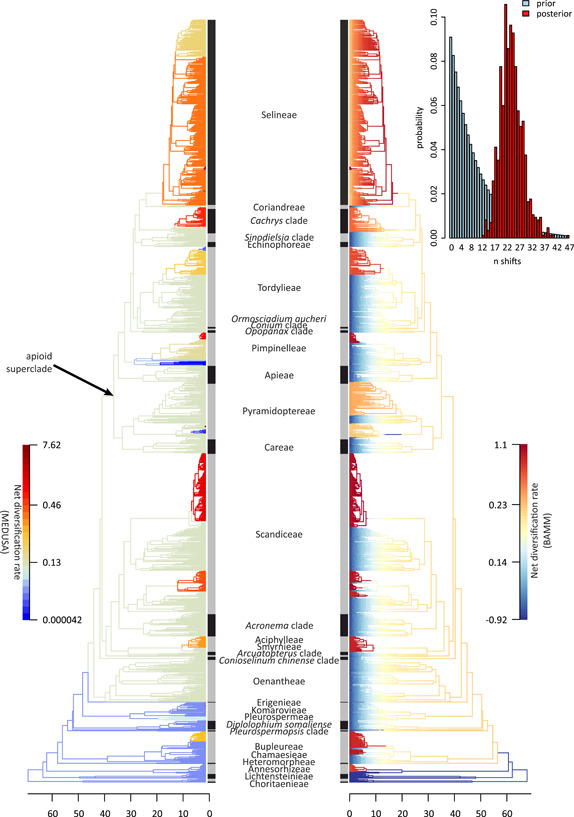
Shifts in net diversification rates as estimated with MEDUSA (left) and BAMM (right) analyses. The plot in the top‐right corner shows prior and posterior probabilities for inferred number of shifts (*n*) in BAMM. Thermal gradient for BAMM was scaled with Jenks natural breaks method (nonlinear) because rates across the phylogeny were skewed toward higher values. Due to the large number of investigated models, we show only the results for the “mixed” MEDUSA and BAMM analyses with five expected shifts and Poisson rate prior equaling 0.05, both with specified richness. The results of the remaining trait‐independent diversification analyses are given in Appendix S10

Character‐independent analyses performed with BAMM and MEDUSA point to a high variation in diversification rates within Apioideae, with BAMM reconstructing 26–31 shifts (highest posterior density range) for analyses with specified species richness and 19–22 shifts when this parameter was not provided (Figure 5; Appendix S10G–X). MEDUSA analyses yielded similar results but with fewer inferred rates (e.g., 21 for the mixed process with species richness correction; Figure 5; Appendix S10A–F). Clades consistently linked with elevated diversification rates across all tested models are tribes Selineae, Aciphylleae, and Bupleureae and genera *Pimpinella* (Pimpinelleae), *Heracleum* (Tordylieae), *Chaerophyllum*, and *Ferula* (both Scandiceae). The *Cachrys* clade, Annesorhizeae, and *Bunium* (Pyramidoptereae) were also frequently associated with such an increase.

The analyses with character‐dependent models indicate that the heterogeneity of diversification parameters within Apioideae is independent of the presence or absence of pseudocorollas. The best‐fitting model was a MuCID‐3 (ΔAIC_c_ < 2; Table 2), in which diversification rates do not depend on the state of the observed character. The character‐independent model (CID‐4) was also chosen as the best fit when two types of pseudocorollas were encoded as a single state (Appendix S4C).

## DISCUSSION

The presence of pseudanthia is evolutionary labile with 95% confidence interval for total number of transitions reconstructed as 115–117 and at least 36 independent origins in a moderately large clade like Apioideae (Figure 2). Although limited, data available for several lineages of Asteraceae indicate that, in other plant groups, these structures are also prone to convergence (Francisco‐Ortega et al., 1999; Sanz et al., 2008; Panero et al., 2014). The high evolvability of flower‐like inflorescences can be tentatively explained by the developmental preadaptation and their inability to cause an irreversible phenotypical and ecological specialization, which would hinder eventual losses and re‐gains. According to our results, we hypothesize that the main factors responsible for numerous origins of pseudanthia are optimization of floral display size and adaptive wandering within generalized pollination syndromes. Because evolution of pseudocorollas is unlikely to lead to a pollinator‐mediated speciation, this trait alone cannot explain diversification patterns in Apioideae and is unlikely to provide such an explanation in other plant groups.

### Adaptive wandering of flower‐like inflorescences

Generalist pollination systems are frequently considered suboptimal because flowers have to specialize to mitigate trade‐offs resulting from mutually exclusive demands and qualitative differences between various pollinators (Ohashi et al., 2021). Some generalist plants, however, could have reached a stable equilibrium (adaptive generalization) with a tight adjustment to available pollinator niches. This mechanism, known as adaptive wandering, is expected to lead to evolution of various phenotypic traits that attract more beneficial pollinators without exclusion of others (Wilson and Thomson, 1996; Dilley et al., 2000; Thomson and Wilson, 2008; Gómez et al., 2014, 2015). Because the nature of such adaptations is described as “transient, reversible and idiosyncratic” (Thomson and Wilson, 2008, p. 25), we may expect them to be highly labile and easily lost during evolution. Umbellifers are generally regarded as ecologically “promiscuous” with a large number of visiting pollinators and frequent spatiotemporal fluctuations in their composition (Lamborn and Ollerton, 2000; Davila and Wardle, 2008). Some researchers speculated that, despite superficial generalism, Apiaceae may undergo “cryptic specialization” with variation of some traits resulting from selection toward specific pollination niches (Bell, 1971; Lindsey and Bell, 1985; Pérez‐Bañón et al., 2007; Niemirski and Zych, 2011; Zych et al., 2014, 2019). Pseudocorollas constitute an auxiliary pool of such phenotypic characters that in combination with other traits can be used to reach particular fitness peaks during such adaptive wandering. The high number of transitions (especially reversals) recovered for both types of pseudocorollas (Figure 2) creates a landscape congruent with this evolutionary scenario.

The important question to ask is how specific pseudanthial architectures answer the demands of various pollinators? Our morphospace analysis (Figure 5; Appendices S6, S9) implies existence of disjunct sets of correlated traits in floral and hyperfloral pseudocorollas that in combination with minor phenotypic characters can make displays attractive to particular groups of insects. According to these results, petaloid bracts are strongly associated with yellow or purple flowers and subequal/equal outer and inner umbel rays (making the inflorescence more spherical and less compact). Hyperfloral pseudocorollas found in numerous yellow‐flowered representatives of the genus *Bupleurum* are sometimes intensively colored and very prominent (e.g., *Bupleurum aureum* subsp. *porfirii* Kamelin), making them more appealing to visually sensitive pollinators. Such coloration patterns, however, show some degree of polymorphism. For example, *Hymenidium apiolens* Lindley includes three color variants of involucel margins (white, pink, and purple), probably in response to differences in pollination niches (Guo et al., 2020). White petaloid bracts are frequently associated with deeply purple flowers, which may enhance contrast and serve as visual cues for dipterans, especially carrion flies. Hyperfloral pseudocorollas can additionally have deeply colored, reddish veins (e.g., *Pleurospermum amabile* W.G. Craib & W.W. Sm. or *Vesper purpurascens* (A. Gray) R.L. Hartm. & G.L. Nesom.), which further mimic the appearance of flesh.

A distinct morphology and different evolutionary correlations can be found in species with floral pseudocorollas, the blossoms of which are universally white and frequently create a flat surface due to unequal outer and inner umbel rays. White, inconspicuous flowers are generally considered a mark of generalist pollination systems, but their aggregation in compact, showy pseudanthia may be favored by particular group of insects and beneficial under pollinator limitation. This hypothesis can account for differences between two subspecies of *Heracleum sphondylium* (Zych, 2002, 2007) with apomorphic yellow, loose inflorescences found in *H. sphondylium* subsp. *sibiricum* (L.) Simonk. evolving as a visual cue for lowland beetles, while compact, white‐flowered and pseudocorollar umbels of *H. sphondylium* subsp. *sphondylium* (L.) are a plesiomorphic adaptation to flies and wasps in ancestral montane habitats. Moreover, many apioids with white petals and floral pseudocorollas (e.g., *Daucus carota*, *Artedia squamata*, and some species of *Echinophora* L.) develop so‐called “dark central florets”, which have been thought to attract certain species of coleopterans (Westmoreland and Muntan, 1996; Goulson et al., 2009) and flies (Eisikowitch, 1980) or facilitate pollinator's orientation during foraging (Polte and Reinhold, 2013).

### Pseudanthia evolve in response to constraints on floral display size

It is frequently assumed that the number of pollinator visitations and the number of flowers probed per visit are positively correlated with display size, and these interdependences have been proven true both on theoretical grounds (Ohashi and Yahara, 1999) and an experimental basis (Ohara and Higashi, 1994; Grindeland et al., 2005). The attractivity of large inflorescences is particularly clear for dense, multiflowered units like umbels (Ishii et al., 2008), but their augmentation cannot proceed indefinitely without diminishing returns resulting from the risk of geitonogamy (Robertson, 1992; Harder and Barrett, 1995) and production‐associated costs (Harder and Johnson, 2005). The risk of geitonogamy (pollination between flowers of the same plant) may be particularly high in flat‐surfaced displays as their architecture encourages inconsistent foraging pathways, frequent revisitations, and pollen discounting (Ohashi and Yahara, 2001, 2002; Harder et al., 2004). The opposite size trends (Figure 3) recovered for pseudanthial and non‐pseudanthial inflorescences indicate that similar attractiveness can be achieved either by increasing the number of individual flowers or through the formation of pseudocorollas; the latter are likely to evolve in response to constraints on the former.

The modular architecture of apioid pseudanthia allows for their compactness and promotion to be altered as a means of balancing between the attractiveness to pollinators (overall size of floral display) and offspring genetic diversity (related to the number of visits required to pollinate all reproductive units). In large‐sized umbels, pseudanthia frequently develop at the level of individual umbellets rather than the entire umbel, forming several distinct, smaller units that cannot be freely explored without considerable energetic expense (i.e., flight or prolonged walk). This situation is fairly common in tribe Tordylieae, which has a high prevalence of species with floral pseudocorollas (Figure 2). Conversely, in situations of pollinator limitation or when plant population densities are very high, a strong condensation of umbellets within the umbel into a single pseudanthium may be favored as competitively superior (e.g., *Daucus carota* or *Artedia squamata*).

Massive displays are of great adaptive value under pollinator limitation but require lots of resources and are hardly sustainable. Many umbellifers with such inflorescences (e.g., representatives of *Ferula* L., *Heracleum* or *Angelica*) are monocarpic perennials, which produce spectacular blossoms made of tens of thousands of florets but only once in a lifetime, following several seasons of vegetative growth. In our study, small pseudanthia were reconstructed to evolve predominantly by reduction of larger inflorescences (Figure 3). Because advertising organs demand less resources than flowers (Thomann et al., 2015; Cerca et al., 2019), the development of pseudocorollas may help to diminish the investment in reproductive structures without sacrificing the attractiveness of inflorescence. In this scenario, pseudanthia may first appear as tools for counterbalancing the trade‐offs associated with large displays and can later be co‐opted in adaptive wandering, particularly to facilitate adaptation to habitats where pollinator limitation is common. This mechanism could potentially explain why among apioids pseudanthia are common in many montane or arid‐adapted species, such as representatives of the genera *Tordylium*, *Echinophora*, *Dicyclophora*, or *Pleurospermum*. Interestingly, similar secondarily compartmented or aggregated inflorescences (syncephalia) occurring in Asteraceae subtribe Nassauvinae are also considered an adaptation to dry habitats (Katinas et al., 2008; Katinas and Forte, 2020).

### Developmental perspective on the evolutionary lability of pseudanthia

An interesting question concerning pseudanthia in Apioideae is how can such complex structures be so easily “turned on” and “off” in response to selection pressures. The proximal causes underlying this macroevolutionary phenomenon may to some extent be explained on the grounds of evolutionary developmental biology. As the exact molecular mechanisms underlying the formation of different architectures in compound umbels are currently unknown, the inference in this matter must be made based on the data available for distantly related plants. In the case of *Cornus* L. (Zhang et al., 2008; Feng et al., 2012) and *Davidia* Baill. (Vekemans et al., 2012) from the order Cornales, hyperfloral pseudocorollas are patterned by genes otherwise responsible for petal identity (B‐ and C‐class MADS box genes), whose expression shifted to encompass bracts. This shift had been preceded by the duplication of at least some of the aforementioned genes (most notably, orthologues of *GLOBOSA/PISTILLATA*), allowing paralogues to gain new or additional functions without losing the original one. On the contrary to ray flowers, which are restricted to Apioideae, hyperfloral pseudocorollas are relatively common in all subfamilies of Apiaceae (e.g., *Sanicula epipactis* L. in Saniculoideae, *Pozoa coriacea* Lag. in Azorelloideae, *Actinotus helianthi* Labill. in Mackinlayoideae). As we are not aware of any species with pseudanthia in other families within Apiales (Myodocarpaceae, Araliaceae, Pittosporaceae, Griseliniaceae, Torricelliacaeae, the expansion of MADS‐box genes (or different genetic alteration that accounts for origin of bract petaloidy) must have taken place after the divergence.of umbellifers.

The independent gains of floral pseudocorollas across angiosperms are usually associated with *CYCLOIDEA* genes, which expanded their initial role in the establishment of floral symmetry (Fambrini and Pugliesi, 2017) to control the specialization of florets (Tähtiharju et al., 2012; Claßen‐Bockhoff et al., 2013; Berger et al., 2016; Zhao et al., 2020). The search for *CYCLOIDEA* genes in the genomic data for carrot (Scandiceae) and coriander (Coriandreae) has so far revealed two paralogues, which must have been present in their last common ancestor (both species develop floral pseudocorollas; J. Baczyński et al., unpublished data). Knowing that some representatives of Oenantheae also develop ray flowers, it is probable that the duplication occurred in the common ancestor of these lineages. The subsequent repeated recruitment/de‐recruitment of already existing CYC paralogues could be the “molecular switch” responsible for numerous gains and losses of floral pseudocorollas in Apioideae. This explanation is especially tempting in the light of recent studies documenting exceptionally high number of transitions between actinomorphy and zygomorphy (Reyes et al., 2016; Joly and Schoen, 2021), with the evolution of ray flowers representing a special case of such changes—driven by the same genes but occurring only in some flowers within the inflorescence (Hileman, 2014). The high rate of reversals (losses) inferred for floral pseudocorollas in this study are concordant with the general pattern found for floral symmetry in angiosperms, whereby losses of zygomorphy are more likely to occur than gains when modeled with maximum‐likelihood methods (Sauquet et al., 2017; Reyes et al., 2018). In the future, these important issues concerning evolvability of flower‐like inflorescences may be properly addressed with hidden Markov models such as the precursor model (Marazzi et al., 2012; Tarasov, 2019).

### Diversification of Apioideae

The phylogeny of Apioideae was shaped by extraordinarily numerous shifts in diversification rates, consistently exceeding 10 in all variants of trait‐independent analyses and reaching more than 30 for some runs of BAMM. Similar numbers have not been recorded in any other study of comparable phylogenetic scale (Baker and Couvreur, 2013; Reyes et al., 2015; Panero and Crozier, 2016; Xue et al., 2020) and are closer to the family‐level analyses of all flowering plants with 30 shifts found by Magallón et al. (2019) and 27 by Tank et al. (2015). MEDUSA estimates in the mega‐scale analysis of Smith and Brown (2018) recovered only 10 shifts in Apioideae.

Our understanding of ecological and morphological evolution in apioids (and Apiales in general) is full of gaps, and it remains difficult to reliably identify major drivers of their diversification. The explosive radiation of some clades identified in our study could have arisen from hybridization/introgression events (Panahi et al., 2015), long‐distance dispersal (Sun et al., 2004; Chung et al., 2005) or expansion of gene families involved in the synthesis of biochemical compounds (Krieger et al., 2018), but none of these traits have been subject to detailed analyses. Although pseudanthia cannot explain diversification patterns observed in the entire subfamily (Table 2; Appendix S4C), they could have played an important role on a smaller scale, facilitating radiation of some large, panboreal genera such as *Heracleum*, the pseudocorollar representatives of which include many invasive, noxious weeds (Jahodová et al., 2007).

## CONCLUSIONS

Our study indicates that flower‐like inflorescences are scattered throughout the apioid phylogeny and are extremely labile evolutionarily, with recurrent gains and reversals occurring in most of the lineages that evolved this architecture. Like many other traits of Apioideae, pseudanthia may arise due to adaptive generalization and optimal use of local pollination niches. Furthermore, pseudocorollas can be used to mitigate trade‐offs associated with the enlargement of floral display, especially under pollinator limitation. Surprisingly, the presence of pseudanthia cannot sufficiently explain the high variation in diversification rates recovered for Apioideae. The ecological importance of different pseudanthial architectures can be extrapolated from more general patterns, but without experimental studies on generalized pollination systems and reaching beyond “floricentrism” such hypotheses will remain highly speculative.

## AUTHOR CONTRIBUTIONS

J.B. with H.S. and K.S. developed ideas for the manuscript; J.B. gathered the data, performed analyses, and wrote the manuscript; H.S. and K.S. edited the manuscript.

## Supporting information


**Appendix S1**. List of accessions for molecular markers used in phylogenetic reconstruction.

**Appendix S2**. Morphological data set with a complete list of references.

**Appendix S3**. Information about fossils used for phylogeny calibration with age and node justifications and references.

**Appendix S4**. Graphs illustrating transition rate matrices (A, B) used in corHMM and trait‐dependent diversification analyses with pseudocorollas encoded as a single state and results of the model‐fitting (C) and stochastic mapping (D) conducted for this variant of analyses.

**Appendix S5**. Graphs with transition rate matrices used in corHMM (A) and trait‐dependent diversification analyses (B) and marginal likelihood ancestral character estimation conducted separately for types of pseudocorollas (C) and size (D).

**Appendix S6**. Results of the betadisper (A) and NMDS (B) analyses.

**Appendix S7**. Four Excel sheets containing information about species richness provided for MEDUSA (A) or BAMM (B), calculations of sampling fraction for trait‐dependent analyses (C), and data on apioid biodiversity according to Plants of the World Online database (POWO) (D).

**Appendix S8**. Phylogenetic tree of apioids with mapped bootstrap/SH‐aLRT support. All major clades/tribes are collapsed into triangles.

**Appendix S9**. Plots of all possible two‐dimensional combinations for the first five principal components.

**Appendix S10**. Phylorate plots for all variants of MEDUSA (A–F) and BAMM (G–X) analyses.

## Data Availability

All sequence alignments (before and after trimming) and maximum likelihood tree are available from TreeBase repository (www.treebase.org) under Study Accession URL: http://purl.org/phylo/treebase/phylows/study/TB2:S29132.
