# Supplementary material for: Exceptional evolutionary lability of flower‐like inflorescences (pseudanthia) in Apiaceae subfamily Apioideae
Source: Am J Bot. 2022 Mar 20;109(3):437–55. doi: 10.1002/ajb2.1819 (PMC9310750; doi:10.1002/ajb2.1819)
Supplement: Supplementary file 3 — Appendix S3. Information about fossils used for phylogeny calibration with age and node justifications and references. [file AJB2-109-437-s009.docx]

Baczyński et al.—American Journal of Botany 2021—Appendix S3

**APPENDIX S3.** Information about fossils used for phylogeny calibration with age and node justifications and references.

**Fossil 1**

**Organs:** pollen
**Locality:** laminated claystones in Beipaizi
**Formation:** Daotiaqiao
**Country:** China

**Reference (description):**

Manchester, S. R., Grímsson, F., & Zetter, R. (2015). Assessing the fossil record of asterids in the context of our current phylogenetic framework. *Annals of the Missouri Botanical Garden* 100(4), 329.

**Safe minimum age:** 12 Ma **Absolute age source:** stratigraphic (upper limit of oldest stratigraphic age)
**Oldest stratigraphic age:** Late Miocene (Serravallian)
**Referenece time scale:** ICS (v2020/03)

**Age justification**

*“The claystone belongs to the lower part of a sedimentary succession composing the Daotiaqiao Formation. The age of the Daotaiqiao Formation has been assigned to the late middle Miocene to early late Miocene (approximately 12--11 Ma), by correlating well-dated fossil macro- and microfloras in East Asia (*[*Liu et al., 1995*](https://www.ncbi.nlm.nih.gov/pmc/articles/PMC6485501/#R88)*,*[*1996*](https://www.ncbi.nlm.nih.gov/pmc/articles/PMC6485501/#R89)*;*[*Liu, 1998*](https://www.ncbi.nlm.nih.gov/pmc/articles/PMC6485501/#R87)*; [Leng, 1997](https://www.ncbi.nlm.nih.gov/pmc/articles/PMC6485501/#R83),*[*2000a*](https://www.ncbi.nlm.nih.gov/pmc/articles/PMC6485501/#R84)*,*[*2000b*](https://www.ncbi.nlm.nih.gov/pmc/articles/PMC6485501/#R85)*), and by the correlation of vertebrate faunas, including various fossil freshwater fish (*[*Chang et al., 1996*](https://www.ncbi.nlm.nih.gov/pmc/articles/PMC6485501/#R15)*) and fossils of terrestrial mammals (*[*Qi, 1992*](https://www.ncbi.nlm.nih.gov/pmc/articles/PMC6485501/#R155)*).”*

**Reference (age)**

Manchester, S. R., Grímsson, F., & Zetter, R. (2015). Assessing the fossil record of asterids in the context of our current phylogenetic framework. *Annals of the Missouri Botanical Garden* 100(4), 329.

**Node calibrated:** Apioid superclade

**Node justification**

Manchester et al. (2015): *“Apiaceae pollen is also recognizable in the fossil record. We illustrate examples from the middle to late Miocene of Beipaizi, northeast China (Fig. 18). The prolate tricolporate grains show a distinctive rugulate and striate to verrucate and fossulate ornamentation.”*

Baczyński et al. (2021): *“Most of their [apioid superclade/Scandiceae and relatives] representatives have perprolate pollen grains with relatively short ectocolpus (usually not exceeding 65% of polar length), bone-shaped outline in colpus view and crenate or undulate tectum at mesocolpium. […] Representatives of apioid superclade tend to have more pronounced columellae increasing total wall thickness and sexine to nexine ratio.”*

**Reference (relationships)**

Manchester, S. R., Grímsson, F., & Zetter, R. (2015). Assessing the fossil record of asterids in the context of our current phylogenetic framework. *Annals of the Missouri Botanical Garden* 100(4), 329.

**Fossil 2 (investigated by authors)**

**Organs:** pollen
**Locality:** limestones in Ebulbahar section of Kars-Erzurum-Muş basin
**Formation:** Yazla (Kelereş)
**Country:** Turkey

**Reference (description):**

Sancay, R. H., Bati, Z., Işik, U., Kirici, S., & Akça, N. (2006). Palynomorph, Foraminifera, and Calcareous Nannoplankton Biostratigraphy of Oligo--Miocene Sediments in the Muş Basin, Eastern Anatolia, Turkey. *Turkish Journal of Earth Sciences* *15*(3).

**Safe minimum age:** 23.03 Ma **Absolute age source:** stratigraphic (upper limit of oldest stratigraphic age)
**Oldest stratigraphic age:** Late Oligocene (Chattian)
**Referenece time scale:** ICS (v2020/03)

**Age justification**

*The zone may correspond to the lower part of DO3, Late Oligocene, (top of the youngest acme of* Deflandrea *spp.) of Biffi & Manum (1988), the* Distatodinium biffii *(Dbi) Interval Zone of Brinkhuis et al. (1992) representing Late Oligocene–Early Miocene, and the* Distatodinium biffii *(Dbi) Interval Zone of Zevenboom (1996) indicating Late Oligocene. The two latter studies suggest that the FO [first occurence] of* Distatodinium biffii *defines the base of the zone whereas the base of the youngest acme of* Deflandrea *spp. can be used as a confirmatory event for the top.*

**Reference (age)**

Sancay, R. H., Bati, Z., Işik, U., Kirici, S., & Akça, N. (2006). Palynomorph, Foraminifera, and Calcareous Nannoplankton Biostratigraphy of Oligo--Miocene Sediments in the Muş Basin, Eastern Anatolia, Turkey. *Turkish Journal of Earth Sciences* *15*(3).

**Node calibrated:** Scandiceae and relatives

**Node justification**

Sancay (2005): *“Small, dicolporate [probably a mistake, should be TRIcolporate] pollen grains. It seems constricted in the middle with distinct colpi.”*

Banasiak et al. (2013): *“Additionally, fossil pollen of higher apioids was reported from the Oligocene–Miocene boundary (Sancay et al., 2006). This pollen may be attributed to a monophyletic group comprising Scandiceae, Smyrnieae, Aciphylleae and the Acronema clade (Ł.B. & K.S., unpublished data).”*

**References (relationships)**

Sancay, R. H. (2005). Palynostratigraphic and Palynofacies Investigation of the Oligocene-Miocene units in the Kars-Erzurum-Mus sub-basins (Eastern Anatolia). *Middle East Technical University, Ankara, Turkey*.

Banasiak, Ł., Piwczyński, M., Uliński, T., Downie, S. R., Watson, M. F., Shakya, B., & Spalik, K. (2013). Dispersal patterns in space and time: a case study of Apiaceae subfamily Apioideae. *Journal of Biogeography* 40(7).

**Fossil 3 (investigated by authors)**

**Organs:** pollen
**Locality:** marl in Zirnak section of Kars-Erzurum-Muş basin
**Formation:** Zirnak
**Country:** Turkey

**Reference (description):**

Sancay, R. H., Bati, Z., Işik, U., Kirici, S., & Akça, N. (2006). Palynomorph, Foraminifera, and Calcareous Nannoplankton Biostratigraphy of Oligo--Miocene Sediments in the Muş Basin, Eastern Anatolia, Turkey. *Turkish Journal of Earth Sciences* *15*(3).

**Safe minimum age:** 5.33 Ma **Absolute age source:** stratigraphic (upper limit of oldest stratigraphic age)
**Oldest stratigraphic age:** Upper Miocene (Messinian)
**Referenece time scale:** ICS (v2020/03)

**Age justification**

*The Lower–Middle Miocene Aflkale Formation, represented by limestone, shale, marl, sandstone, gypsum, conformably overlies the Kelerefl Formation. The Adilcevaz Member of the Aflkale Formation is characterized by reefal limestones of Burdigalian age (Demirtafll & Pisoni 1965), and the Aflkale Formation is unconformably overlain by sedimentary and volcano-sedimentary rocks belonging to the Upper Miocene–Pliocene Zirnak Formation.*

**Reference (age)**

Sancay, R. H., Bati, Z., Işik, U., Kirici, S., & Akça, N. (2006). Palynomorph, Foraminifera, and Calcareous Nannoplankton Biostratigraphy of Oligo--Miocene Sediments in the Muş Basin, Eastern Anatolia, Turkey. *Turkish Journal of Earth Sciences* *15*(3).

**Node calibrated:** MRCA of *Turgenia* and *Lisaea*

**Node justification**

Sancay (2006): *“Fungal spores:* Anatolinites dongyingensis, Biporisporites *sp., All in situ palynomorphs except Compositae, Umbelliferae and* Monoporopollenites gramineoides *have quite long stratigraphic ranges (Tertiary). Monoporopollenites gramineoides and small/less ornamented Umbelliferae can be traced back to Upper Oligocene (Batı, 1996 and references therein), but Compositae (tubuliflorae-type) and big/well ornamented Umbelliferae” pollen were believed to be occurred in the beginning of the Miocene.*

From authors: After detailed analysis of palynomorphs from Kars-Erzurum-Basin we were able to find “large/well ornamented umbellifers” only in late Miocene sediments. Their earlier occurrences were doubtful and could not be unequivocally assigned to Apiaceae. Palynomorphs used for calibration have highly apomorphic morphology (extremely large grains for apioid standards with digitate columellae and highly reduced compound aperture) which can be found only in *Turgenia* and *Lisaea* (formerly considered a single genus).

**Reference (relationships)**

Sancay, R. H., Bati, Z., Işik, U., Kirici, S., & Akça, N. (2006). Palynomorph, Foraminifera, and Calcareous Nannoplankton Biostratigraphy of Oligo--Miocene Sediments in the Muş Basin, Eastern Anatolia, Turkey. *Turkish Journal of Earth Sciences* *15*(3).

**Fossil 4**

**Organs:** fruits
**Locality:** fluvio-lacustrine sediments near Porto da Cruz
**Formation:** Funchal unit, Upper Volcanic complex
**Country:** Portugal (Madeira)

**Reference (description):**

Góis‐Marques, C. A., de Nascimento, L., Fernández‐Palacios, J. M., Madeira, J., & Menezes de Sequeira, M. (2019). Tracing insular woodiness in giant Daucus (sl) fruit fossils from the Early Pleistocene of Madeira Island (Portugal). *TAXON* 68(6), 1314-1320.

**Safe minimum age:** 1.30 Ma **Absolute age source:** radioisotopic
**Oldest stratigraphic age:** Lower Pleistocene (Clabarian)
**Referenece time scale:** ICS (v2020/03)

**Age justification**

*“To test these predictions, we have sampled the Early Pleistocene Porto da Cruz lacustrine and fluvial sediments for plant fossils that could confirm the GDM and GSM extinction predictions. Additionally, two new 40Ar/39Ar geochronological analyses were performed, constraining the age of the sediments to 1.3 Ma (Calabrian).”*

**Reference (age)**

Góis-Marques, C. A., Mitchell, R. L., de Nascimento, L., Fernández-Palacios, J. M., Madeira, J., & de Sequeira, M. M. (2019). Eurya stigmosa (Theaceae), a new and extinct record for the Calabrian stage of Madeira Island (Portugal): 40Ar/39Ar dating, palaeoecological and oceanic island palaeobiogeographical implications. *Quaternary Science Reviews* 206, 129-140.

**Node calibrated:** MRCA of *Daucus decipiens* and *Daucus edulis*

**Node justification**

Sancay (2006): *“When compared with large fruits of Macaronesian neoendemic Apiaceae, the fossils studied fall within extant* Melanoselinum decipiens *mericarp morphological variability (Fig. 2D), especially in their size and in the morphology of the marginal wings, which are irregularly toothed to undulate, smooth surfaced, and auriculate. The extant mericarps of* M. decipiens *are pubescent (Fig. 2D), differing from the fossils (Table 1). Most probably, hairs were lost during diagenesis.* Monizia edulis *differs from the fossils due to the smaller teeth and the truncate to emarginate base of the fruit (see Table 1).”*

**Reference (relationships)**

Góis‐Marques, C. A., de Nascimento, L., Fernández‐Palacios, J. M., Madeira, J., & Menezes de Sequeira, M. (2019). Tracing insular woodiness in giant Daucus (sl) fruit fossils from the Early Pleistocene of Madeira Island (Portugal). *TAXON* 68(6), 1314-1320.
