# Supplementary material for: Exceptional evolutionary lability of flower‐like inflorescences (pseudanthia) in Apiaceae subfamily Apioideae
Source: Am J Bot. 2022 Mar 20;109(3):437–55. doi: 10.1002/ajb2.1819 (PMC9310750; doi:10.1002/ajb2.1819)
Supplement: Supplementary file 4 — Appendix S4. Graphs illustrating transition rate matrices (A, B) used in corHMM and trait‐dependent diversification analyses with pseudocorollas encoded as a single state and results of the model‐fitting (C) and stochastic mapping (D) conducted for this variant of analyses. [file AJB2-109-437-s002.pdf]

Appendix S4. Graphs illustrating transition rate matrices used in corHMM and trait-dependent diversification analyses (A, B) with pseudocorollas encoded as a single state and results of the model-fitting (C) and stochastic mapping (D) conducted for this variant of analyses.

Appendix S4A. Graphical illustration of all matrices used in HiSSE analysis, including transition and diversification rate parameters (wrapped arrows pointing towards associated state). Letters A, B and C (e.g. ABSENT A) indicate the presence hidden states.

Appendix S4B. Graphical illustration of all transition rate matrices used in corHMM analyses; ER and ARD**.**

Appendix S4C. HiSSE models fitted for presence of pseudocorollas in trait-dependent diversification analyses. Abbreviations “Div. pars” and “Trans. pars” denote the numbers of diversification rate parameters and transition rate parameters, respectively. The best-fitting model as chosen by AICc is marked in boldface.

Appendix S4D. S**IMMAP reconstruction for presence of pseudocorolla in Apiaceae subfamily Apioideae averaged across 500 stochastic character-mapped trees.** Pie charts at each node represent the probabilities for each state (grey: pseudocorollas absent, green: pseudocorollas present). Boxes on the right side of the tree indicate tip states. Histograms represent the inferred number of origins and reversals for pseudocorollas. The plot below histograms shows a visualization of the transition rate matrix resolved by corHMM, with arrow widths proportional to transition rates.

Appendix S4A. Graphical illustration of all matrices used in HiSSE analysis, including transition and diversification rate parameters (wrapped arrows pointing towards associated state). Letters A, B and C (e.g. ABSENT A) indicate the presence hidden states.

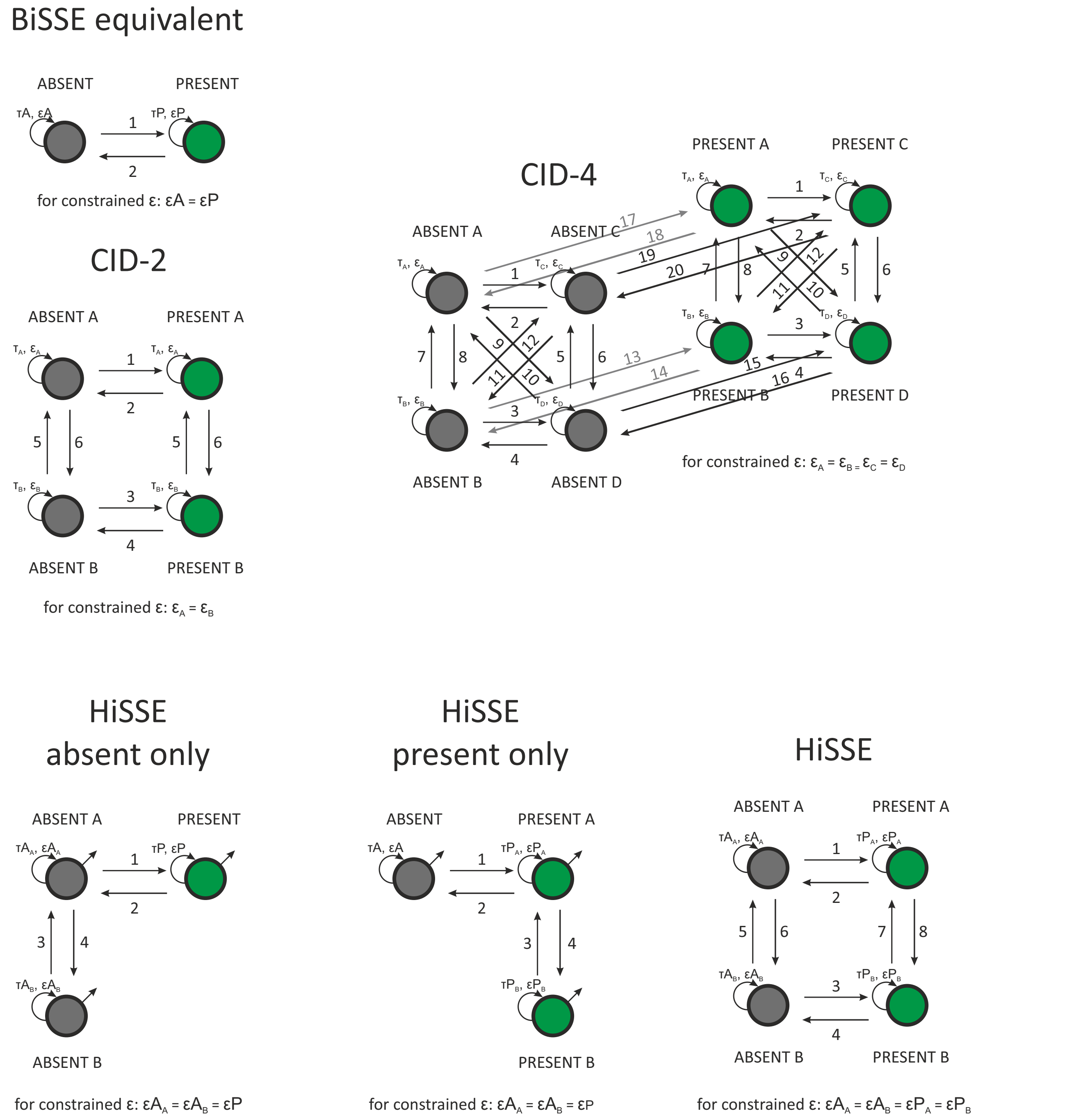

Appendix S4B. Graphical illustration of all transition rate matrices used in corHMM analyses; ER and ARD.

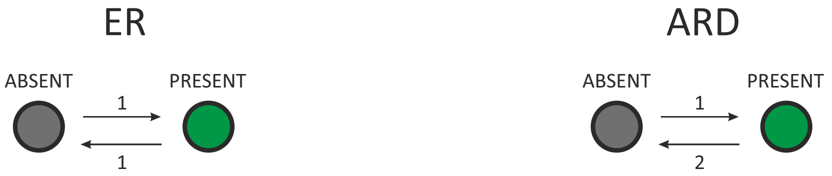

Appendix S4C. HiSSE models fitted for presence of pseudocorollas in trait-dependent diversification analyses. Abbreviations "Div. pars" and "Trans. pars" denote the numbers of diversification rate parameters and transition rate parameters, respectively. The best-fitting model as chosen by AICc is marked in boldface.

Model	Hidden traits	ln L	Div. pars	Trans. pars	AICc
BiSSE equivalent (constrained ε)	0	-3145.7	3	2	6301.5
BiSSE equivalent	0	-3149.9	4	2	6312.0
CID-2 (constrained ε)	1	-2651.8	3	12	5321.8
CID-2	1	-2649.3	4	12	5318.9
CID-4 (constrained ε)	3	-2601.9	5	20	5255.4
**CID-4**	**3**	**-2591.2**	**8**	**20**	**5240.3**
HiSSE absent (constrained ε)	1	-2664.1	4	4	5344.4
HiSSE absent	1	-2646.9	6	4	5316.1
HiSSE present (constrained ε)	1	-2668.5	4	4	5353.2
HiSSE present	1	-2668.1	6	4	5356.4
HiSSE all states (constrained ε)	1	-2646.9	5	8	5316.1
HiSSE all states	1	-2658.9	8	8	5346.4

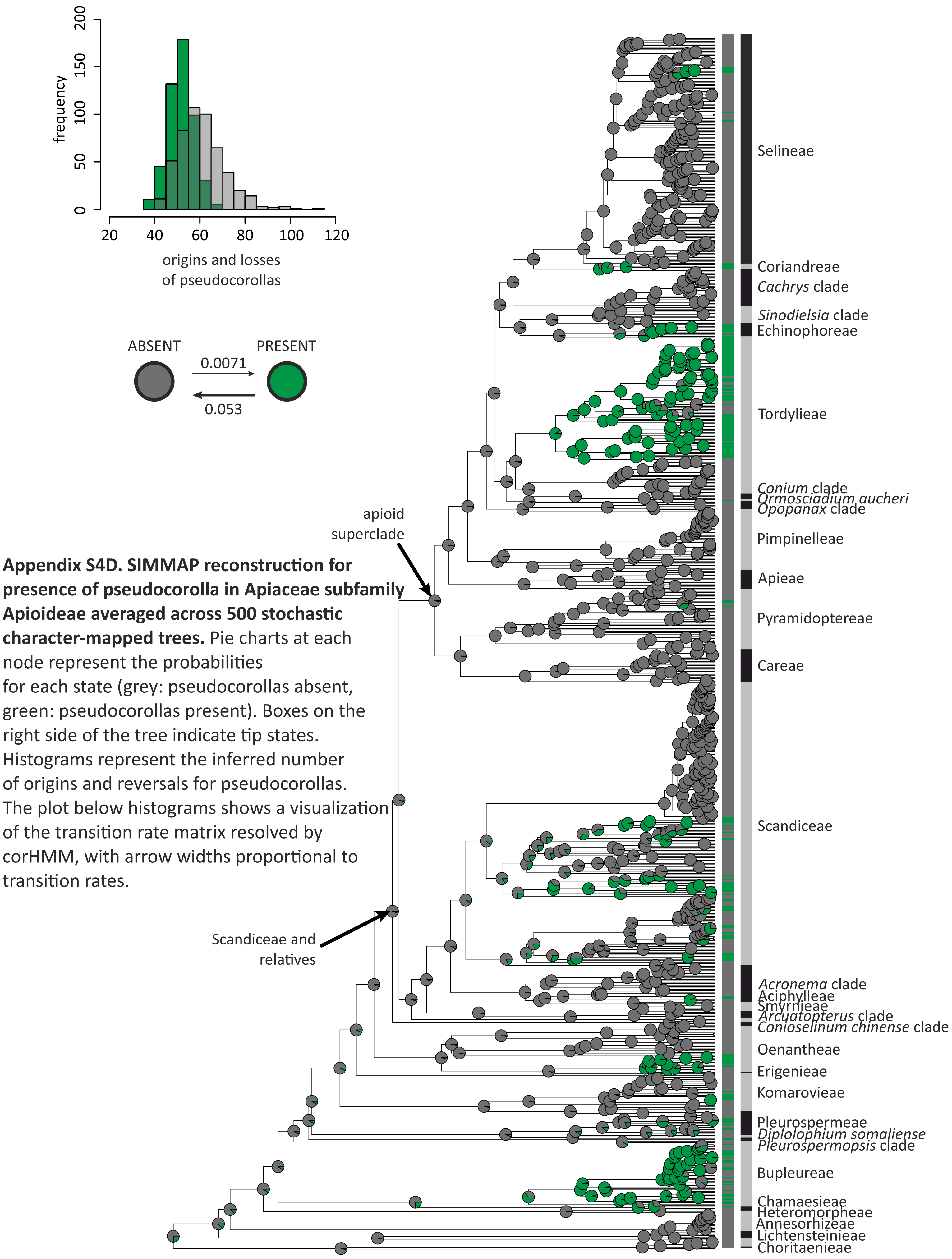

Appendix S4D. SIMMAP reconstruction for presence of pseudocorolla in Apiaceae subfamily Apioideae averaged across 500 stochastic character-mapped trees. Pie charts at each node represent the probabilities for each state (grey: pseudocorollas absent, green: pseudocorollas present). Boxes on the right side of the tree indicate tip states. Histograms represent the inferred number of origins and reversals for pseudocorollas. The plot below histograms shows a visualization of the transition rate matrix resolved by corHMM, with arrow widths proportional to transition rates.
