# Supplementary material for: Exceptional evolutionary lability of flower‐like inflorescences (pseudanthia) in Apiaceae subfamily Apioideae
Source: Am J Bot. 2022 Mar 20;109(3):437–55. doi: 10.1002/ajb2.1819 (PMC9310750; doi:10.1002/ajb2.1819)
Supplement: Supplementary file 5 — Appendix S5. Graphs with transition rate matrices used in corHMM (A) and trait‐dependent diversification analyses (B) and marginal likelihood ancestral character estimation conducted separately for types of pseudocorollas (C) and size (D). [file AJB2-109-437-s006.pdf]

Appendix S5. Graphs with transition rate matrices used in corHMM (A) and trait-dependent diversification analyses (B) and marginal likelihood ancestral character estimation conducted separately for types of pseudocorollas (C) and size (D).

Appendix S5A. Graphical illustration of all transition rate matrices used in corHMM analyses; ordered/unordered size, ordered/unordered types of pseudocorollas and correlated/uncorellated size with types of pseudcorollas.

Appendix S5B. Graphical illustration of all matrices used in MuHiSSE analysis, including transition and diversificationrate parameters (wrapped arrows pointing towards associated state). Letters A, B and C (e.g. FLORAL A) indicate the presence hidden states.

Appendix S5C. Maximum likelihood reconstruction of ancestral states for pseudocorollas conducted according to ordered ARD model (the best fit). The plot in the top-left corner shows a solution of the transition rate matrix, with arrow width proportional to transition rates. Boxes on the right side of the tree indicate tip states.

Appendix S5D. Maximum likelihood reconstruction of ancestral states for inflorescence size conducted according to ordered ARD model (the best fit). The plot in the top-left corner shows a solution of the transition rate matrix, with arrow width proportional to transition rates. Boxes on the right side of the tree indicate tip states.

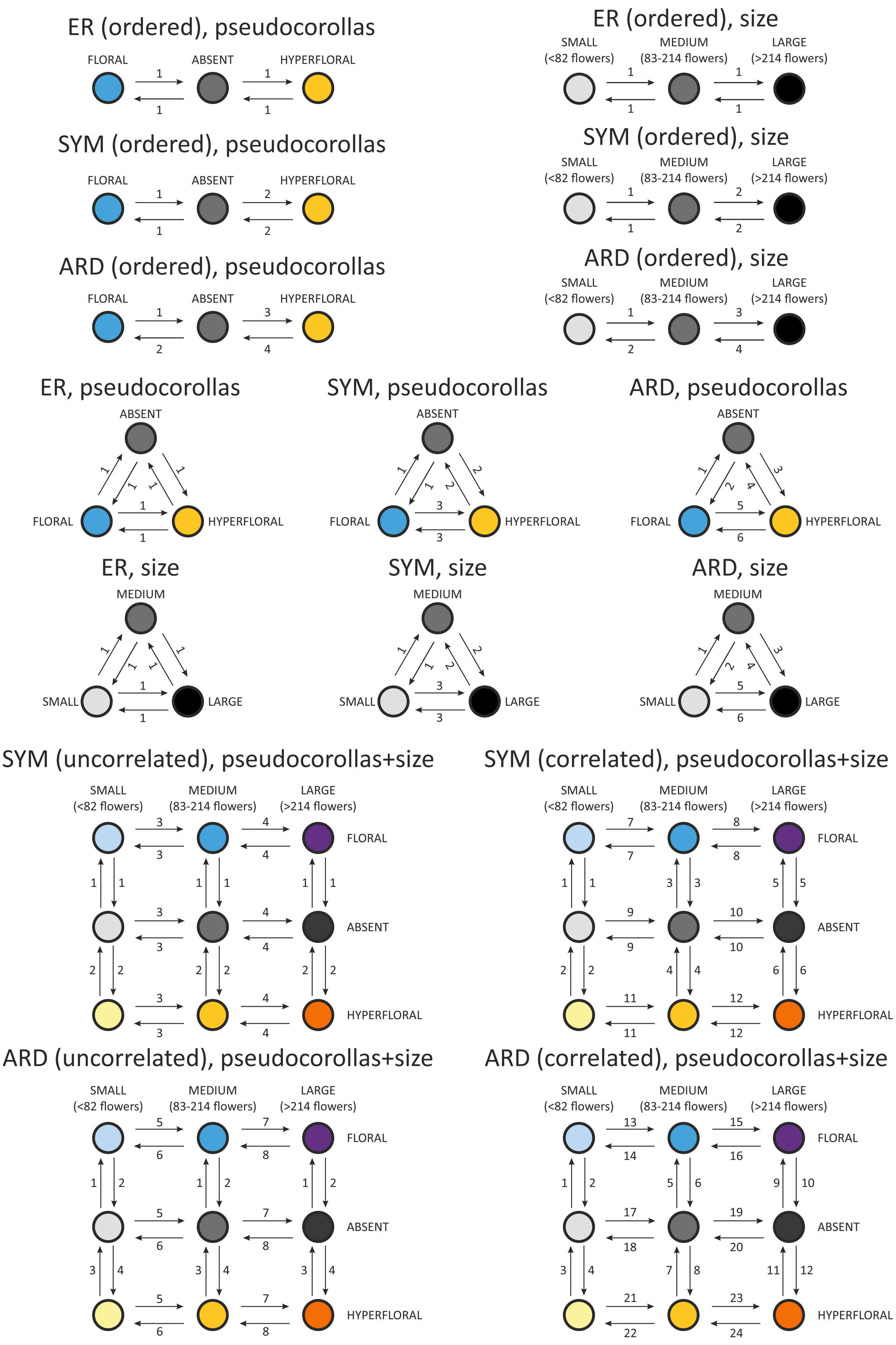

Appendix S5A. Graphical illustration of all transition rate matrices used in corHMM analyses; ordered/unordered size, ordered/unordered types of pseudocorollas and correlated/uncorrelated size with types of pseudcorollas.

Appendix S5B. Graphical illustration of all matrices used in MuHiSSE analysis, including transition and diversification rate parameters (wrapped arrows pointing towards associated state). Letters A, B and C (e.g. FLORAL A) indicate the presence hidden states.

MuSSE equivalent

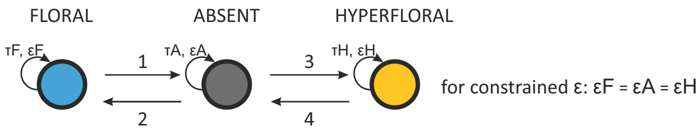

MuCID-3

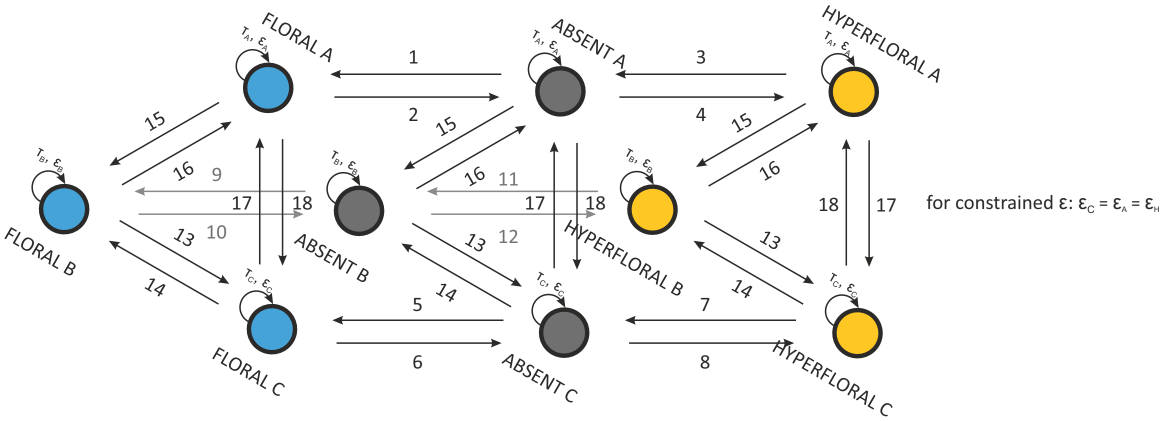

MuHiSSE floral pseudocorollas only

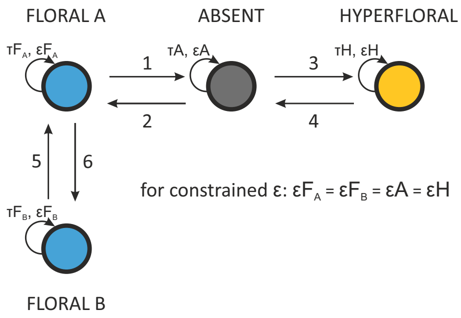

MuHiSSE absent only

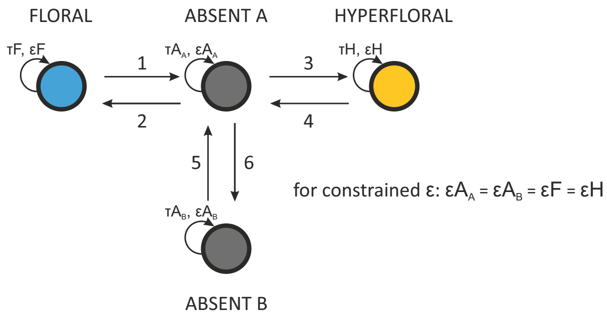

MuHiSSE hyperfloral pseudocorollas only

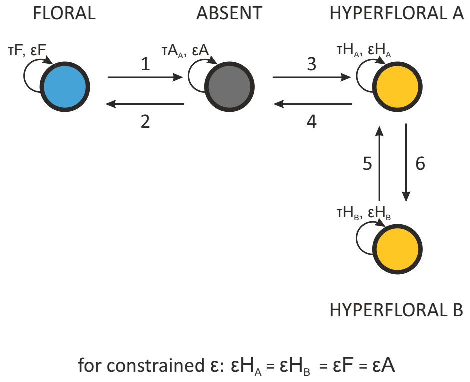

MuHiSSE

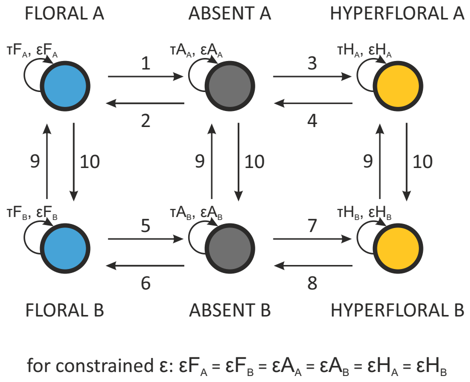

Appendix S5C. Maximum likelihood reconstruction of ancestral states for pseudocorollas conducted according to ordered ARD model (the best fit). The plot in the top-left corner shows a solution of the transition rate matrix, with arrow width proportional to transition rates. Boxes on the right side of the tree indicate tip states.

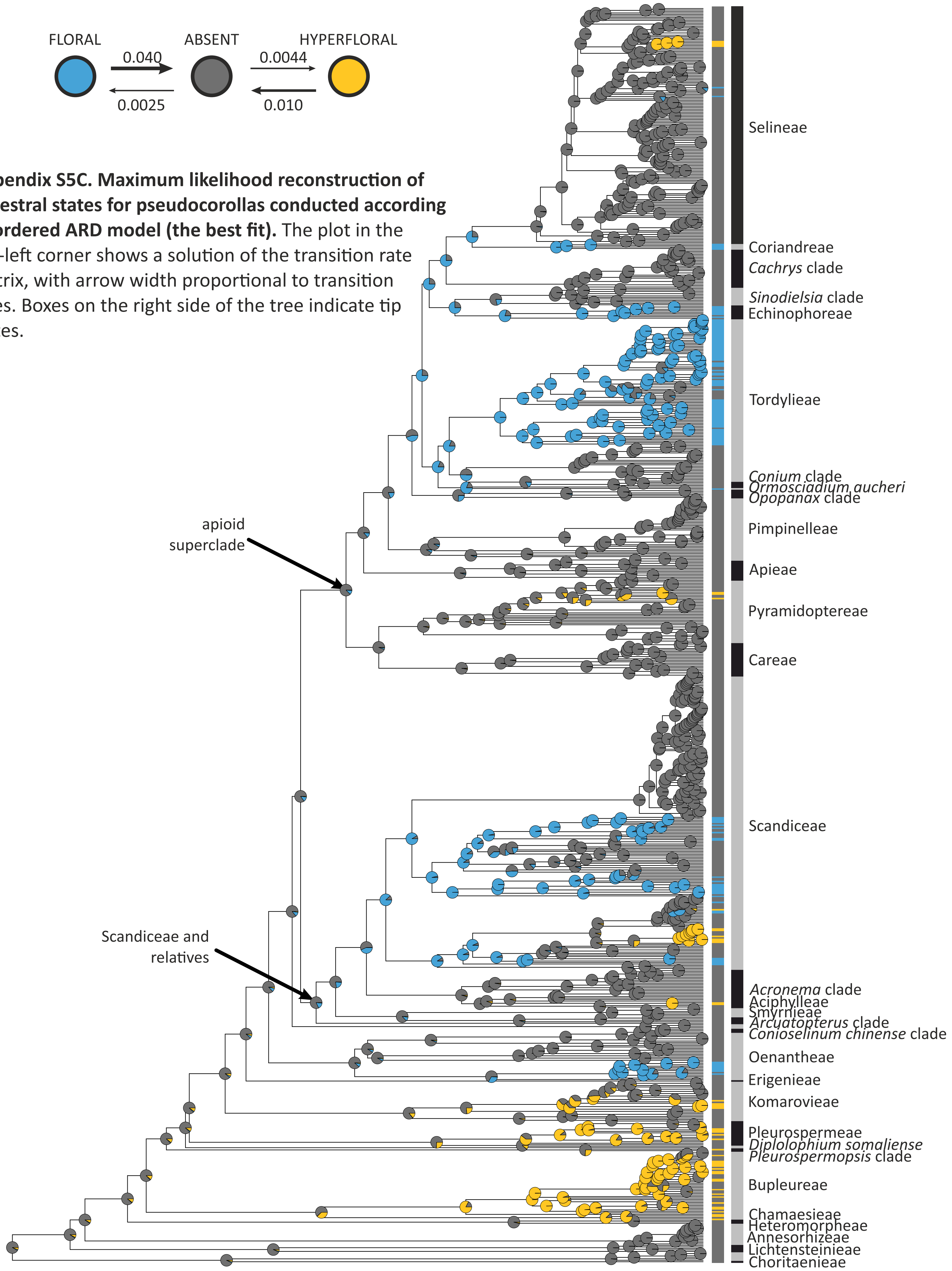

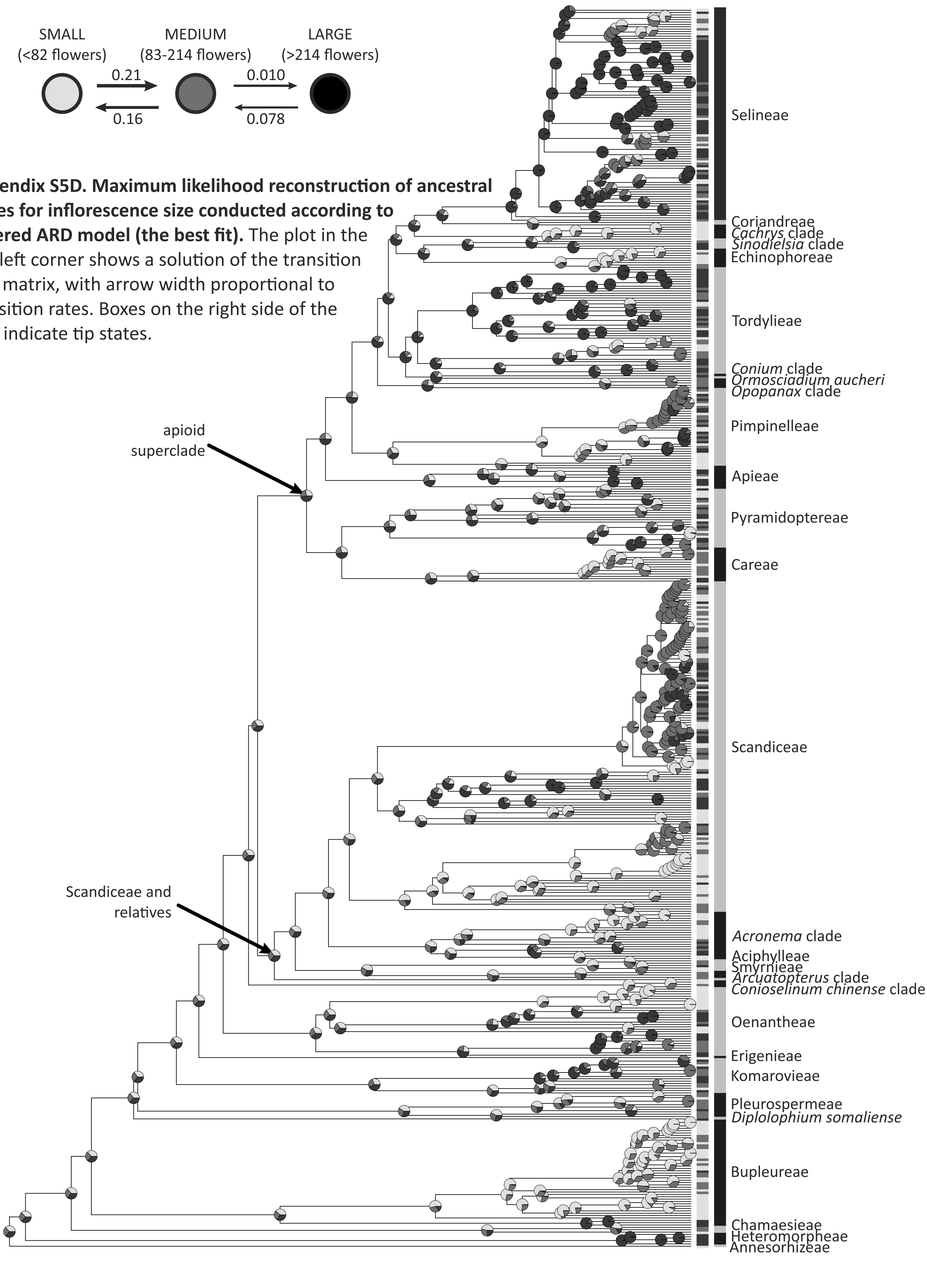

Appendix S5D. Maximum likelihood reconstruction of ancestral states for inflorescence size conducted according to ordered ARD model (the best fit). The plot in the top-left corner shows a solution of the transition rate matrix, with arrow width proportional to transition rates. Boxes on the right side of the tree indicate tip states.
