# Supplementary material for: Exceptional evolutionary lability of flower‐like inflorescences (pseudanthia) in Apiaceae subfamily Apioideae
Source: Am J Bot. 2022 Mar 20;109(3):437–55. doi: 10.1002/ajb2.1819 (PMC9310750; doi:10.1002/ajb2.1819)
Supplement: Supplementary file 6 — Appendix S6. Results of the betadisper (A) and NMDS (B) analyses. [file AJB2-109-437-s008.pdf]

Appendix S6. Results of *betadisper* (A) and NMDS (B) analyses.

Appendix S6A. Non-metric multidimensional scaling (NMDS) ordination of inflorescence architecture in Apioideae. Points correspond to species with non-pseudocorollar umbels (grey), floral (blue) and hyperfloral (yellow) pseudanthia. Convex hulls represent parts of morphospace occupied by species with each of these traits.

Appendix S6B. The visualization of the multivariate dispersion for *betadisper* analysis. Gower distances between objects and group centroids were handled by reducing the original distances to two principal coordinates (PCos) which together explain 42% of variance. Points correspond to species with non-pseudocorollar umbels (grey), floral (blue) and hyperfloral (yellow) pseudanthia. Convex hulls represent the dispersion for each group. Group-wise centroids are marked with red letters (A - absent, F - floral pseudocorollas, H - hyperfloral pseudocorollas).

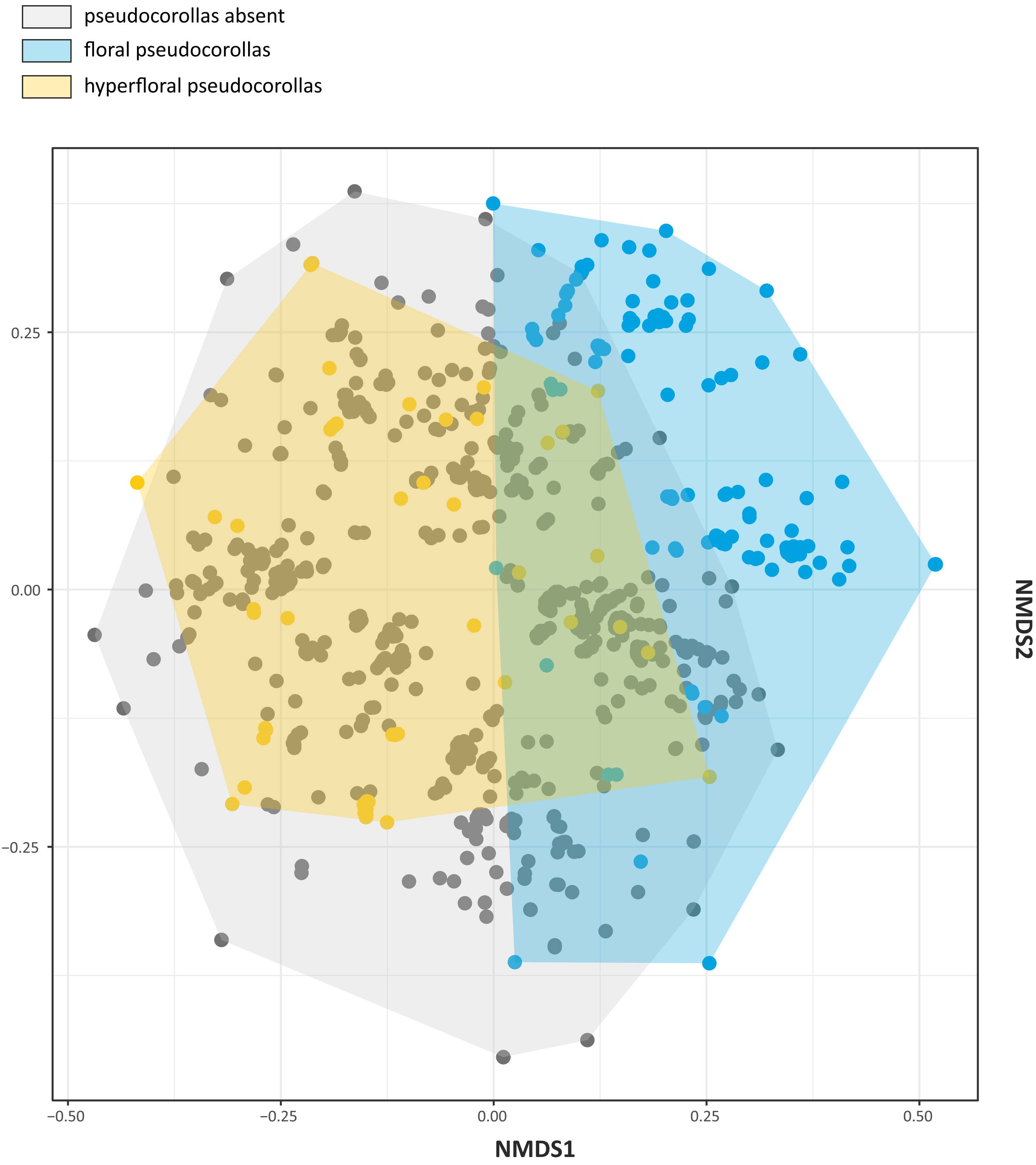

Appendix S6A. Non-metric multidimensional scaling (NMDS) ordination of inflorescence architecture in Apioideae. Points correspond to species with non-pseudocorollar umbels (grey), floral (blue) and hyperfloral (yellow) pseudanthia. Convex hulls represent parts of morphospace occupied by species with each of these traits.

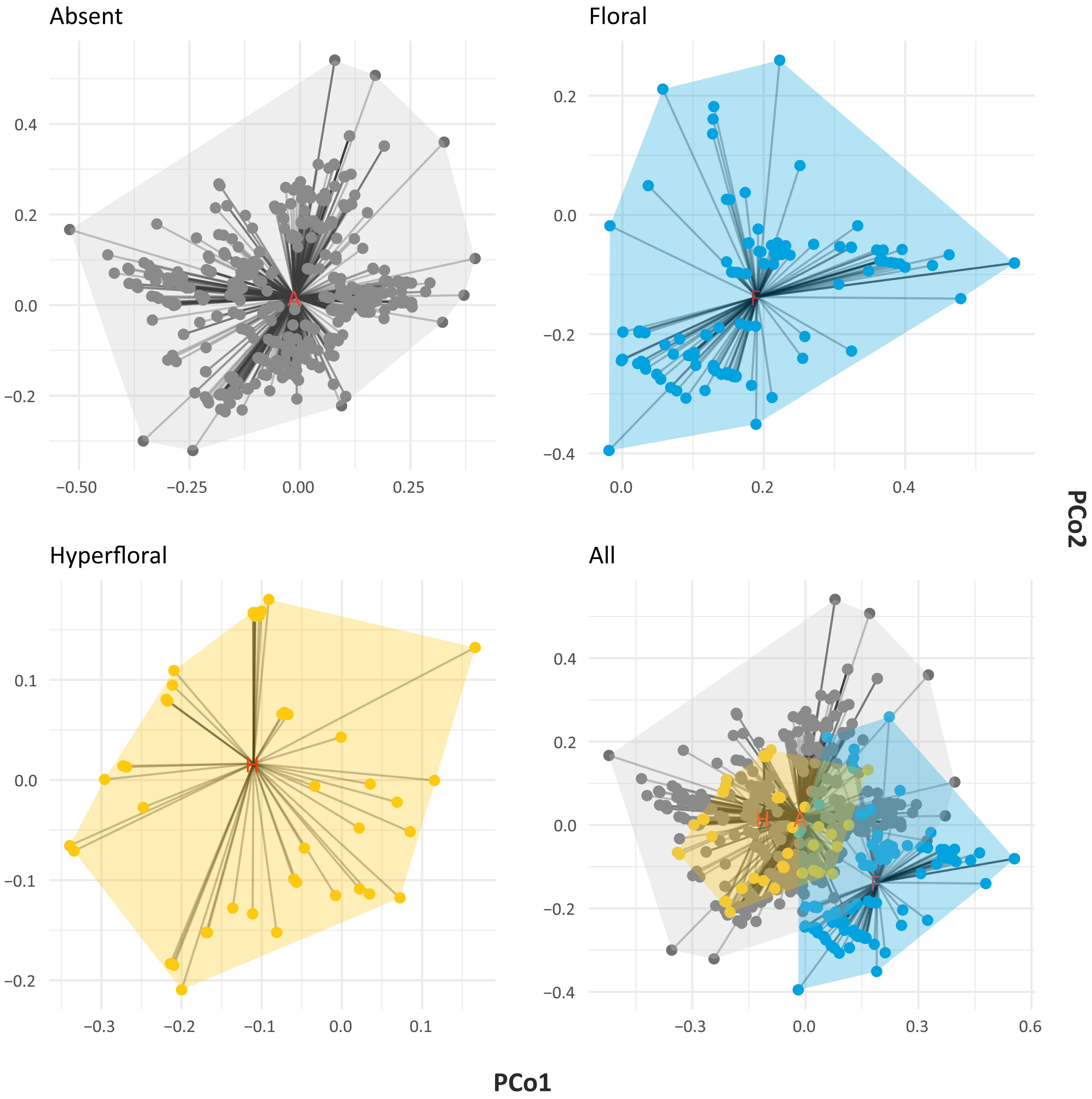

Appendix S6B. The visualization of the multivariate dispersion for *betadisper* analysis. Gower distances between objects and group centroids were handled by reducing the original distances to two principal coordinates (PCos) which together explain 42% of variance. Points correspond to species with non-pseudocorollar umbels (grey), floral (blue) and hyperfloral (yellow) pseudanthia. Convex hulls represent the dispersion for each group. Group-wise centroids are marked with red letters (A - absent, F - floral pseudocorollas, H - hyperfloral pseudocorollas).
