# Supplementary material for: Exceptional evolutionary lability of flower‐like inflorescences (pseudanthia) in Apiaceae subfamily Apioideae
Source: Am J Bot. 2022 Mar 20;109(3):437–55. doi: 10.1002/ajb2.1819 (PMC9310750; doi:10.1002/ajb2.1819)

Appendix S8. Phylogenetic tree of apioids with mapped bootstrap/ SH-aLRT support. All major clades/tribes are collapsed into triangles. Symbols adjacent to nodes indicate that they were supported with SH-aLRT (>0.95, SH), bootstrap (>70, B) or both.

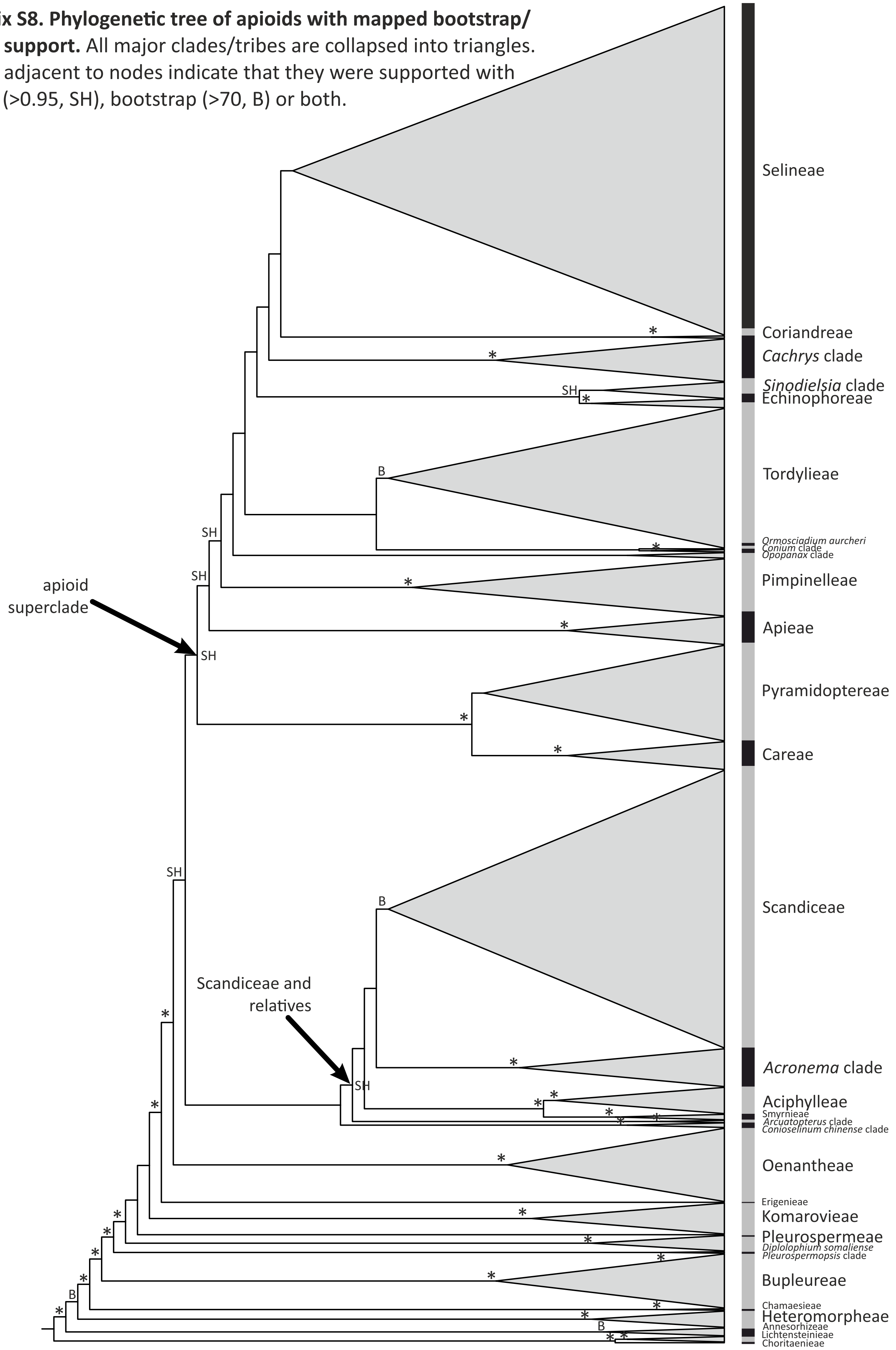

Supplement: Supplementary file 8 — Appendix S8. Phylogenetic tree of apioids with mapped bootstrap/SH‐aLRT support. All major clades/tribes are collapsed into triangles. [file AJB2-109-437-s004.pdf]
