# Supplementary material for: Exceptional evolutionary lability of flower‐like inflorescences (pseudanthia) in Apiaceae subfamily Apioideae
Source: Am J Bot. 2022 Mar 20;109(3):437–55. doi: 10.1002/ajb2.1819 (PMC9310750; doi:10.1002/ajb2.1819)
Supplement: Supplementary file 9 — Appendix S9. Plots of all possible two‐dimensional combinations for the first five principal components. [file AJB2-109-437-s005.pdf]

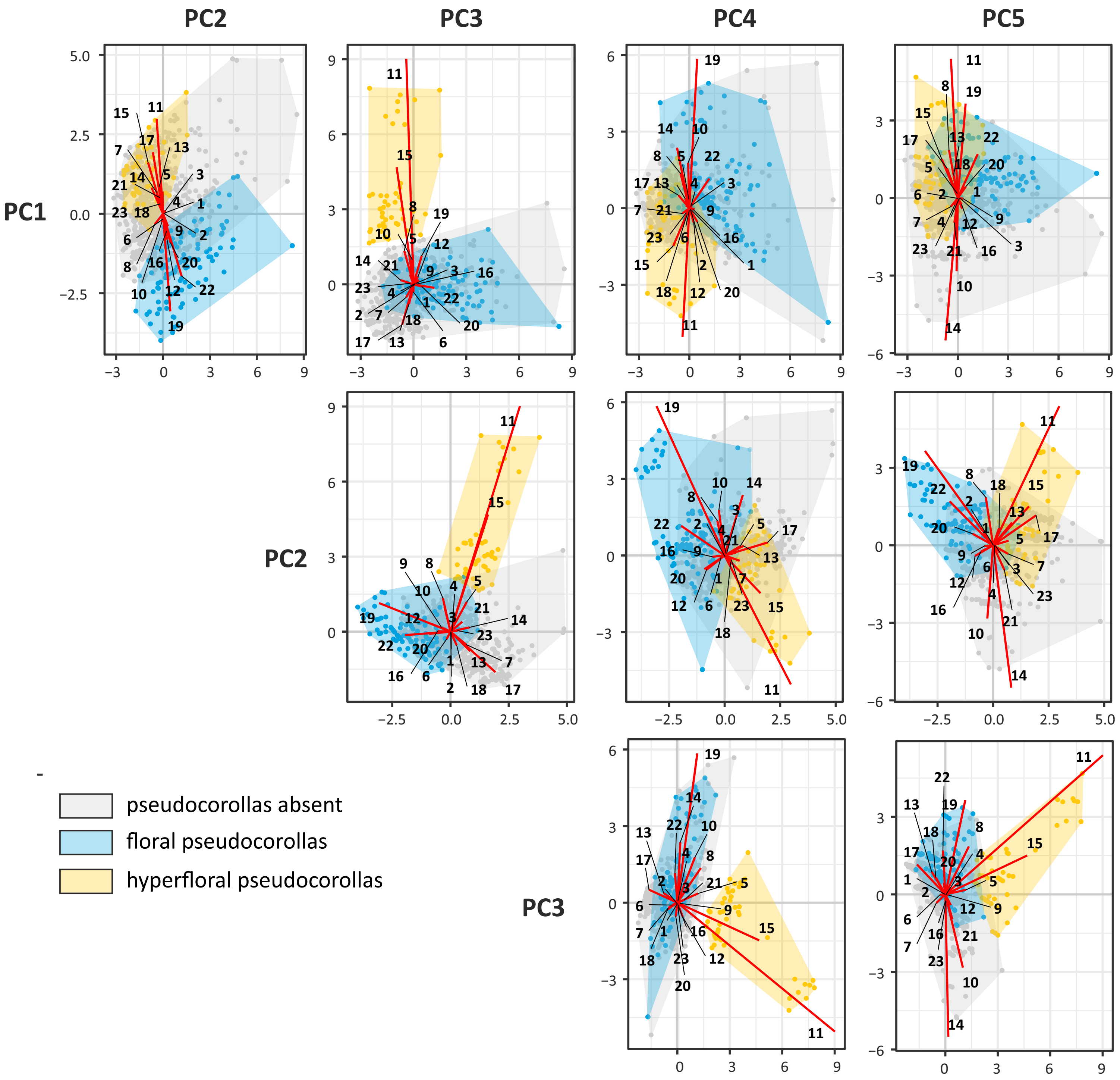

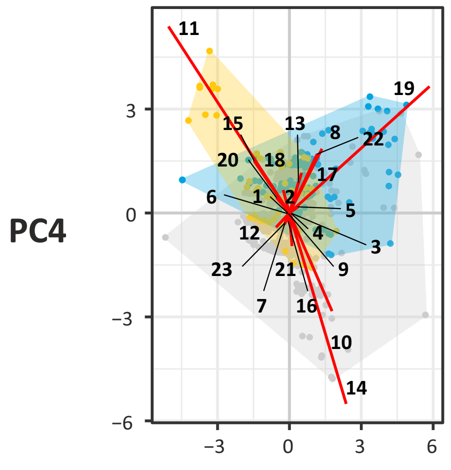

Appendix S9. Principal component analysis (PCA) for the mix of qualitative and quantitative inflorescence traits. The plot shows all possible combinations of the first five principal components (PC1-PC5), which together explain 50.04 % of variance. Each point on the graph represents individual species with colors indicating presence of floral (blue) or hyperfloral (yellow) pseudocorollas. Convex hulls represent parts of morphospace occupied by species with each of these traits.

Numbers at tips of eigenvectors indicate associated trait/state:

1 – minimum number of rays; **2** – maximum number of rays; **3** – minimum number of flowers per umbellet; **4** – maximum number of flowers per umbellet; **5** – umbel rays equal/subequal, **6** – umbel rays unequal; **7** – flowers yellow/yellowish; **8**– flowers purple/purplish; **9** – flowers white; **10** – single bract; **11** – bracts showy; **12** – bracts present; **13** – bracts absent; **14** – single bracteole; **15** – bracteoles showy; **16** – bracteoles present; **17** – bracteoles absent; **18** – sepals minute; **19** – sepals asymmetric; **20** – sepals conspicuous; **21** – sepals absent; **22** – ray flowers present; **23** – ray flowers absent.
