# Supplementary material for: Exceptional evolutionary lability of flower‐like inflorescences (pseudanthia) in Apiaceae subfamily Apioideae
Source: Am J Bot. 2022 Mar 20;109(3):437–55. doi: 10.1002/ajb2.1819 (PMC9310750; doi:10.1002/ajb2.1819)
Supplement: Supplementary file 10 — Appendix S10. Phylorate plots for all variants of MEDUSA (A–F) and BAMM (G–X) analyses. [file AJB2-109-437-s003.pdf]

# **Appendix S10.** Phylorate plots for all variants of MEDUSA (A–F) and BAMM (G–X) analyses.

**Appendix S10A. Phylorate plot for MEDUSA results with mixed model**

**Appendix S10B. Phylorate plot for MEDUSA results with mixed model, without specified richness**

**Appendix S10C. Phylorate plot for MEDUSA results with yule model**

**Appendix S10D. Phylorate plot for MEDUSA results with yule model, without specified richness**

**Appendix S10E. Phylorate plot for MEDUSA results with birth-death model**

**Appendix S10F. Phylorate plot for MEDUSA results with birth-death model, without specified richness**

**Appendix S10G. Phylorate plot for BAMM results with poisson rate prior = 0.05, estimated number of shifts = 5 and without specified richness**

**Appendix S10H. Phylorate plot for BAMM results with poisson rate prior = 0.05, estimated number of shifts = 10 and without specified richness**

**Appendix S10I. Phylorate plot for BAMM results with poisson rate prior = 0.05, estimated number of shifts = 20 and without specified richness**

**Appendix S10J. Phylorate plot for BAMM results with poisson rate prior = 0.05, estimated number of shifts = 5 and with specified richness**

**Appendix S10K. Phylorate plot for BAMM results with poisson rate prior = 0.05, estimated number of shifts = 10 and with specified richness**

**Appendix S10L. Phylorate plot for BAMM results with poisson rate prior = 0.05, estimated number of shifts = 20 and with specified richness**

**Appendix S10M. Phylorate plot for BAMM results with poisson rate prior = 0.1, estimated number of shifts = 5 and without specified richness**

**Appendix S10N. Phylorate plot for BAMM results with poisson rate prior = 0.1, estimated number of shifts = 10 and without specified richness**

**Appendix S10O. Phylorate plot for BAMM results with poisson rate prior = 0.1, estimated number of shifts = 20 and without specified richness**

**Appendix S10P. Phylorate plot for BAMM results with poisson rate prior = 0.1, estimated number of shifts = 5 and with specified richness**

**Appendix S10Q. Phylorate plot for BAMM results with poisson rate prior = 0.1, estimated number of shifts = 10 and with specified richness**

**Appendix S10R. Phylorate plot for BAMM results with poisson rate prior = 0.1, estimated number of shifts = 20 and with specified richness**

**Appendix S10S. Phylorate plot for BAMM results with poisson rate prior = 0.2, estimated number of shifts = 5 and without specified richness**

**Appendix S10T. Phylorate plot for BAMM results with poisson rate prior = 0.2, estimated number of shifts = 10 and without specified richness**

**Appendix S10U. Phylorate plot for BAMM results with poisson rate prior = 0.2, estimated number of shifts = 20 and without specified richness**

**Appendix S10W. Phylorate plot for BAMM results with poisson rate prior = 0.2, estimated number of shifts = 5 and with specified richness**

**Appendix S10V. Phylorate plot for BAMM results with poisson rate prior = 0.2, estimated number of shifts = 10 and with specified richness**

**Appendix S10X. Phylorate plot for BAMM results with poisson rate prior = 0.2, estimated number of shifts = 20 and with specified richness**

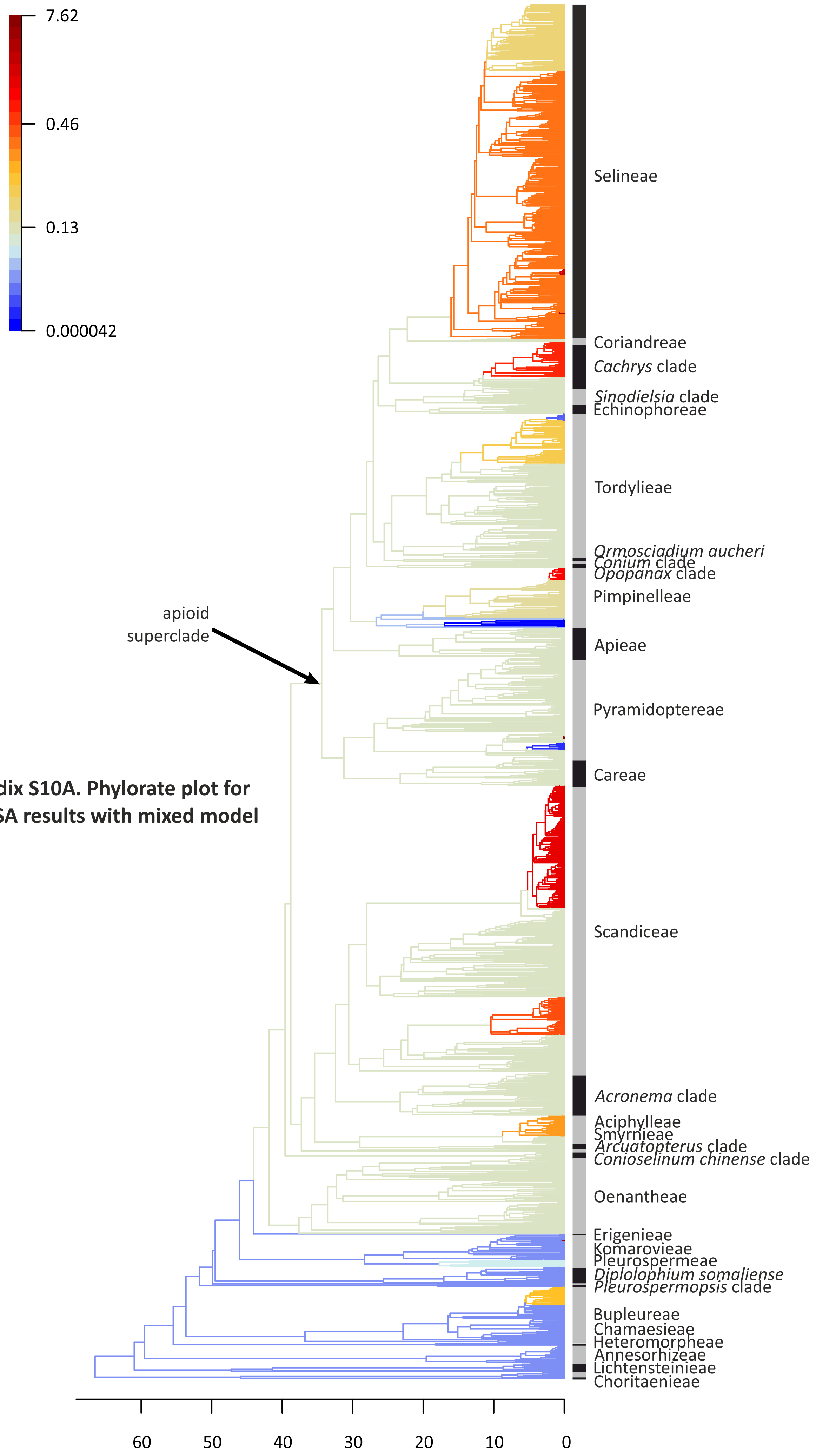


**Appendix S10A. Phylorate plot for MEDUSA results with mixed model**

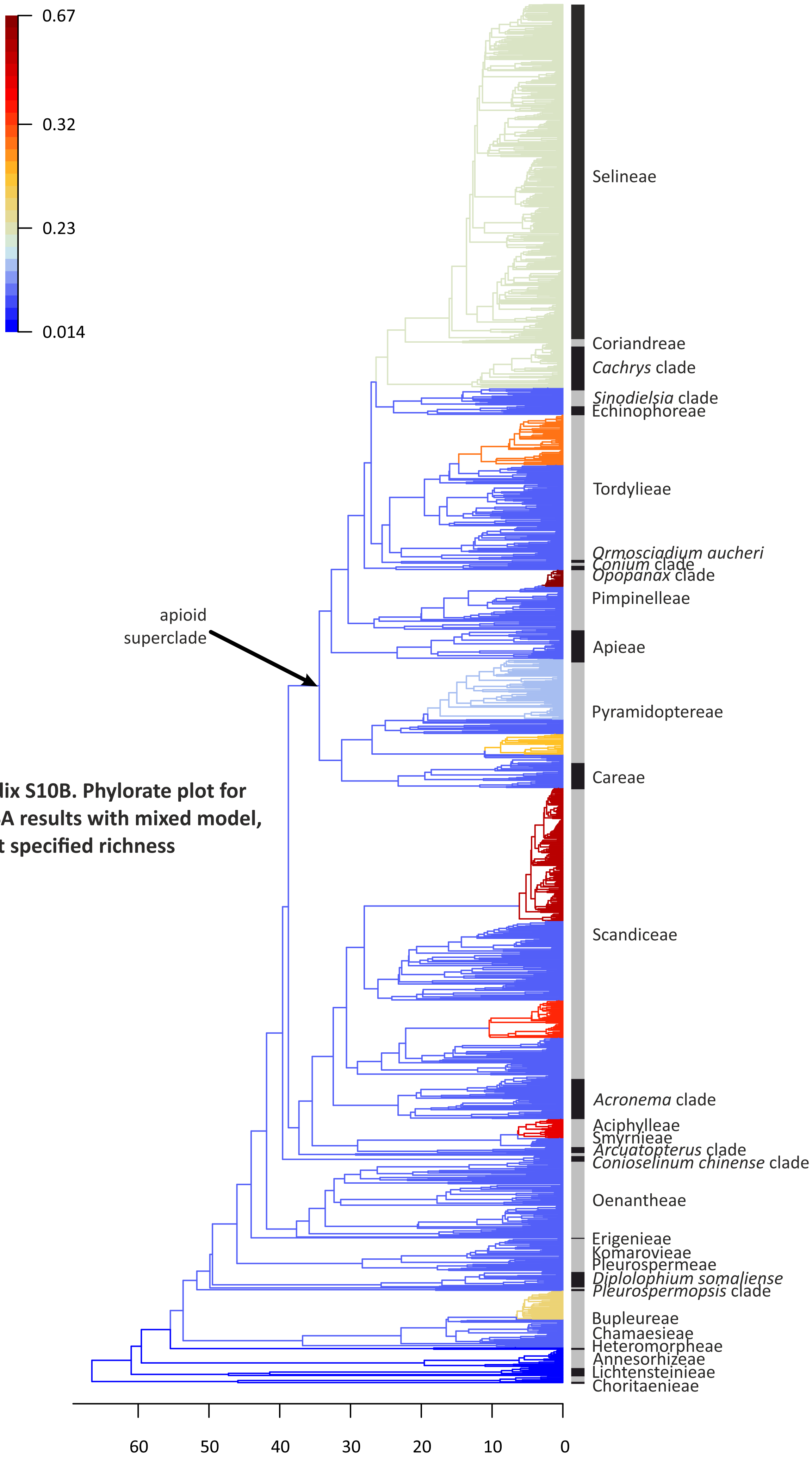


**Appendix S10B. Phylorate plot for MEDUSA results with mixed model, without specified richness**

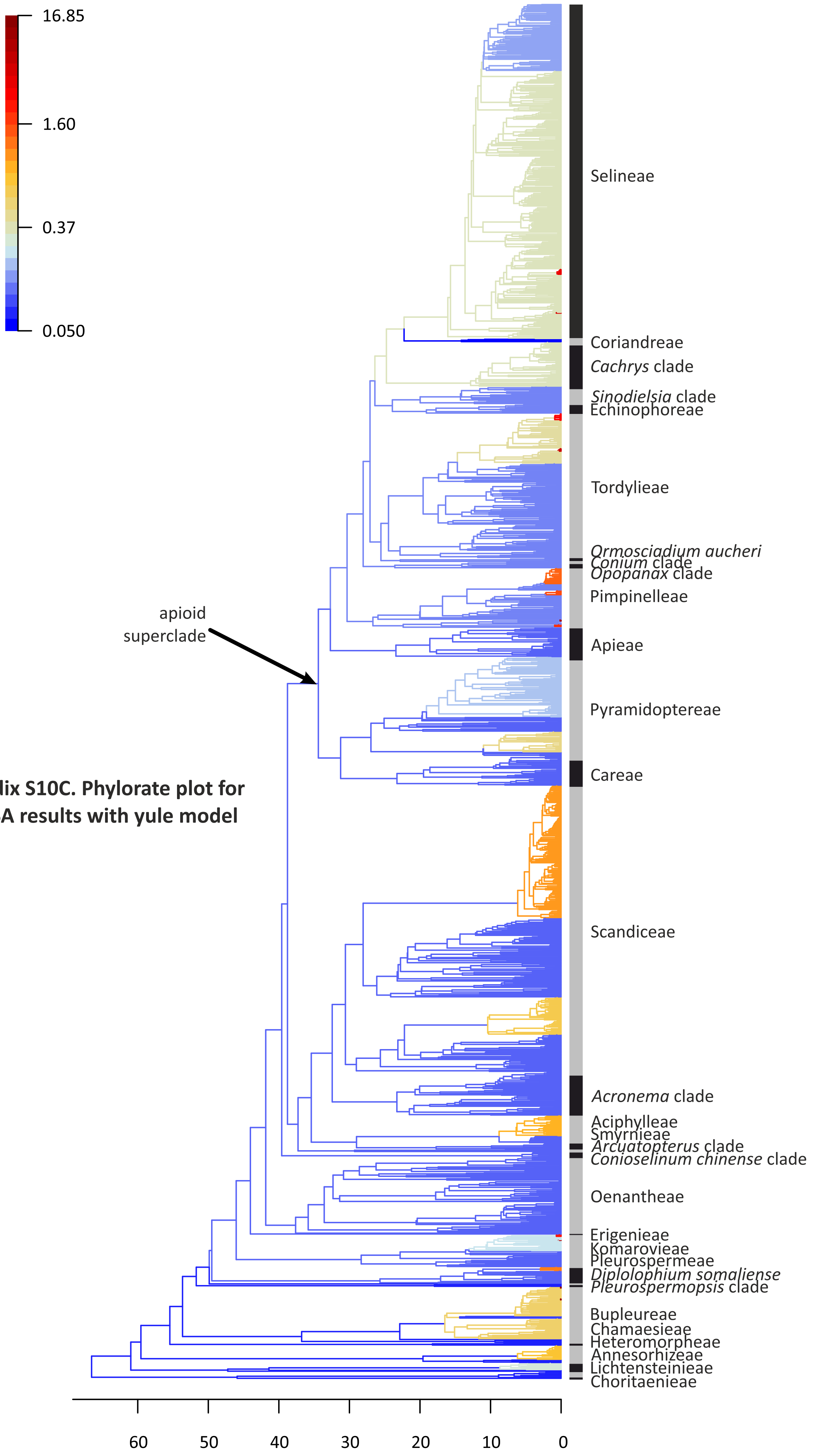


**Appendix S10C. Phylorate plot for MEDUSA results with yule model**

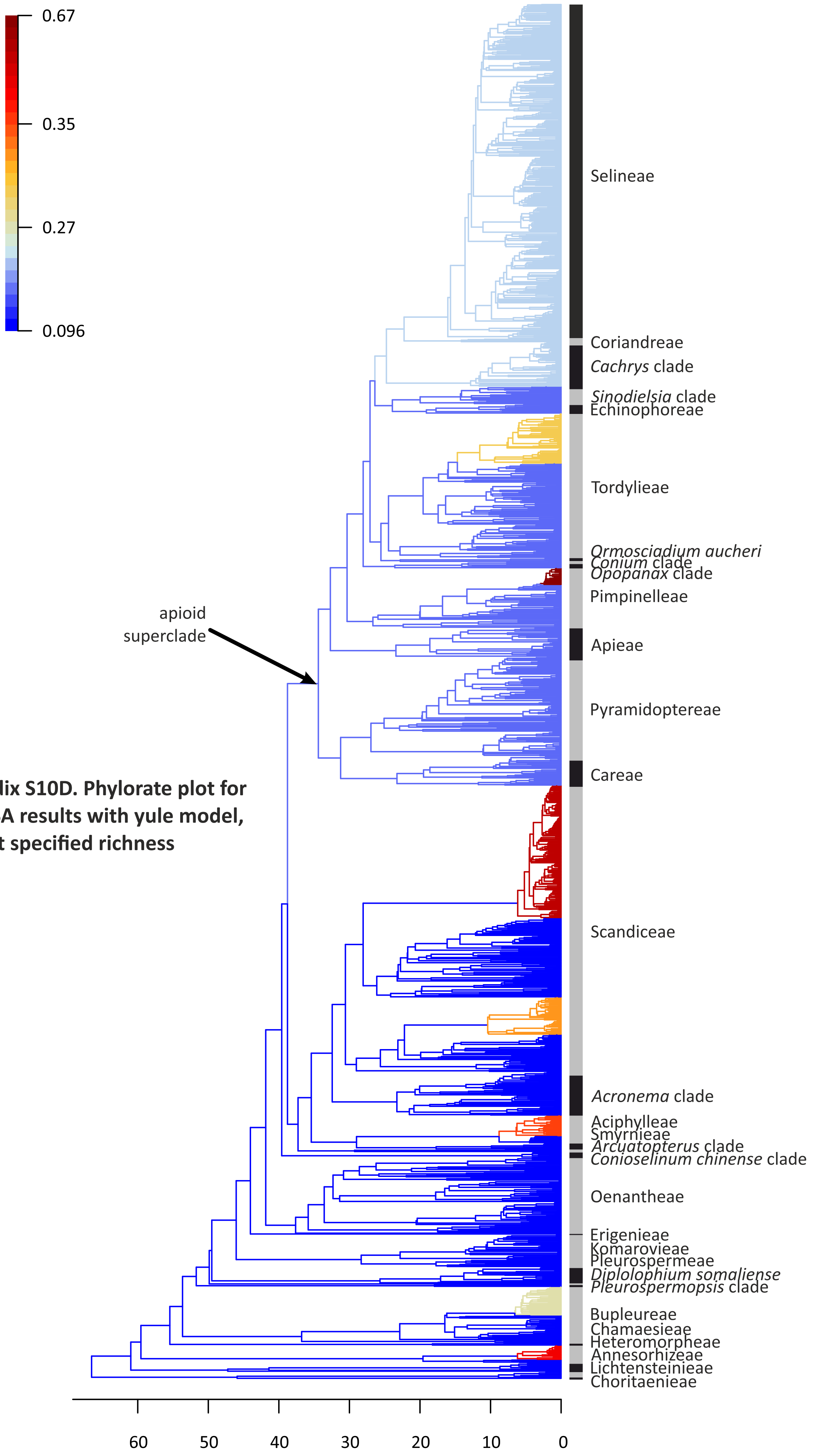


**Appendix S10D. Phylorate plot for MEDUSA results with yule model, without specified richness**

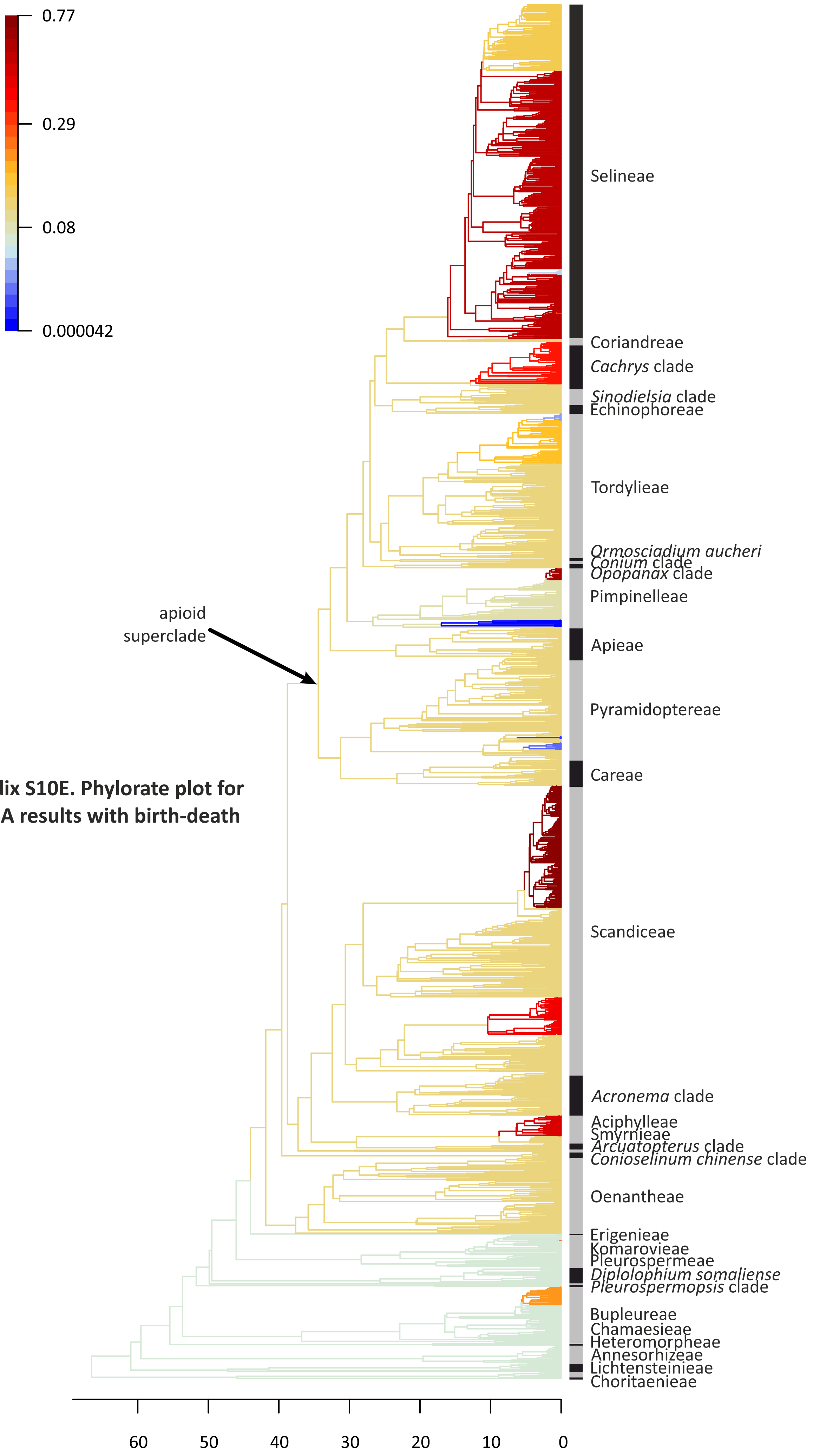


**Appendix S10E. Phylorate plot for MEDUSA results with birth-death model**

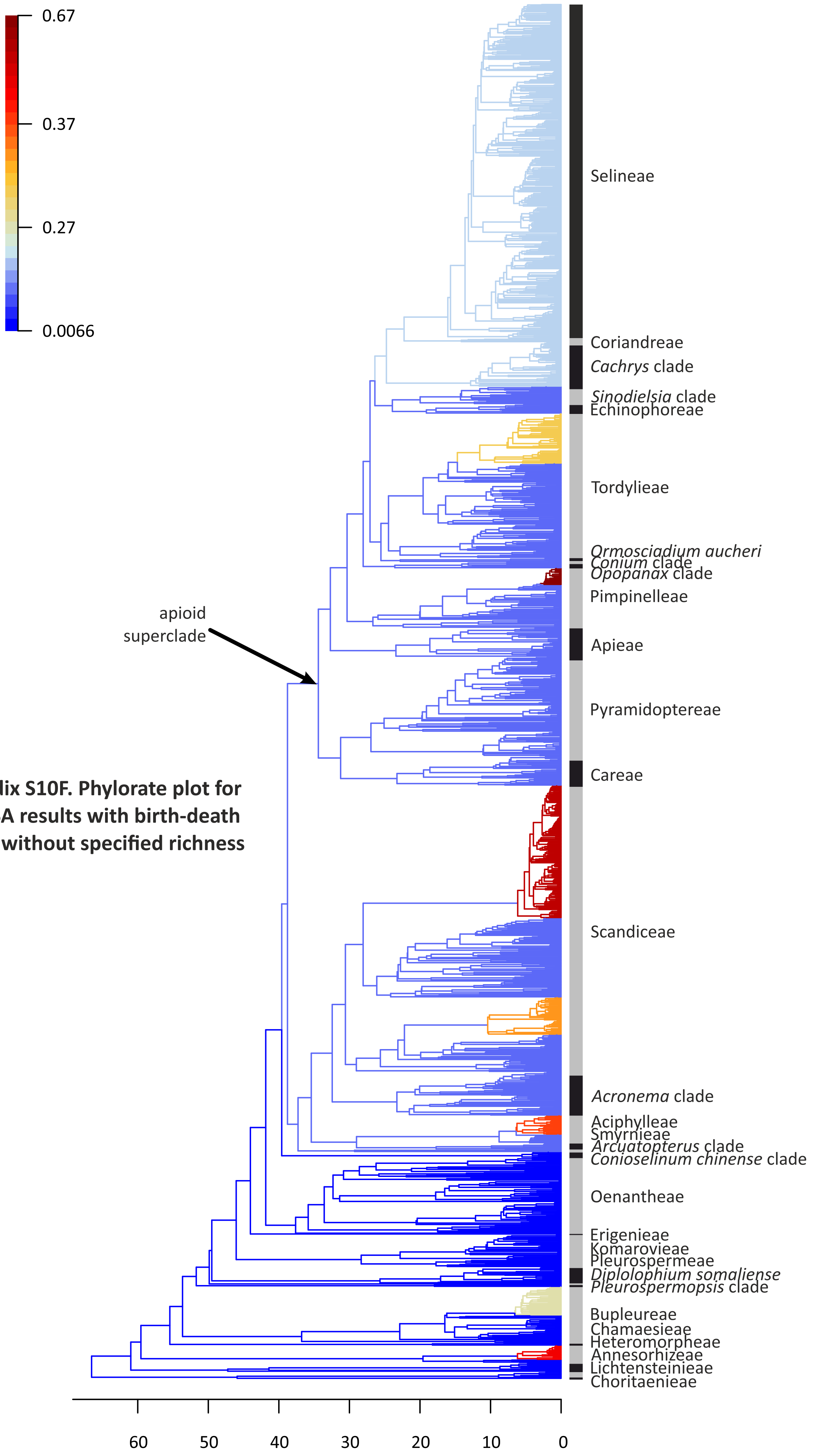


**Appendix S10F. Phylorate plot for MEDUSA results with birth-death model, without specified richness**

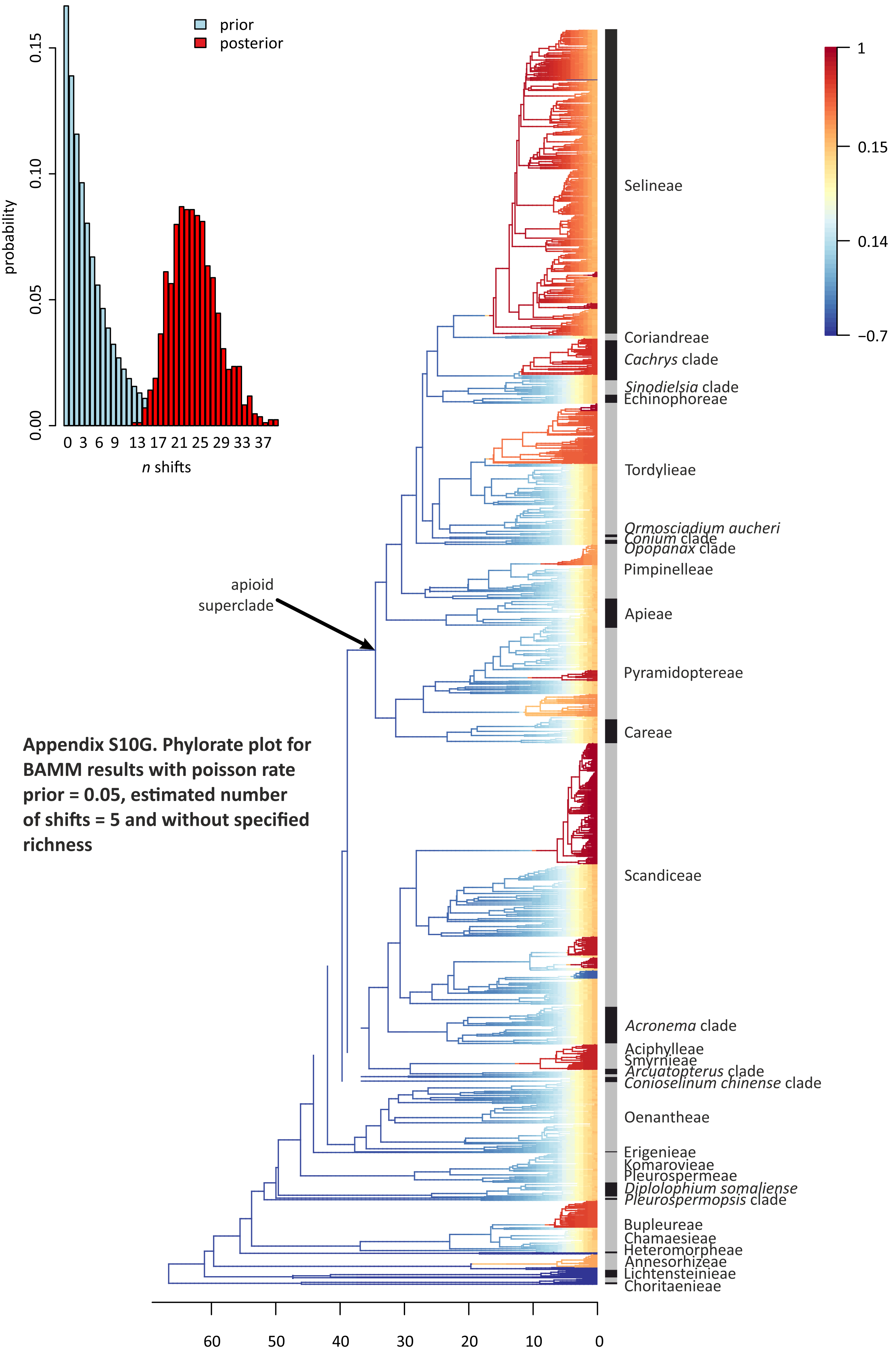


**Appendix S10G. Phylorate plot for BAMM results with poisson rate prior = 0.05, estimated number of shifts = 5 and without specified richness**

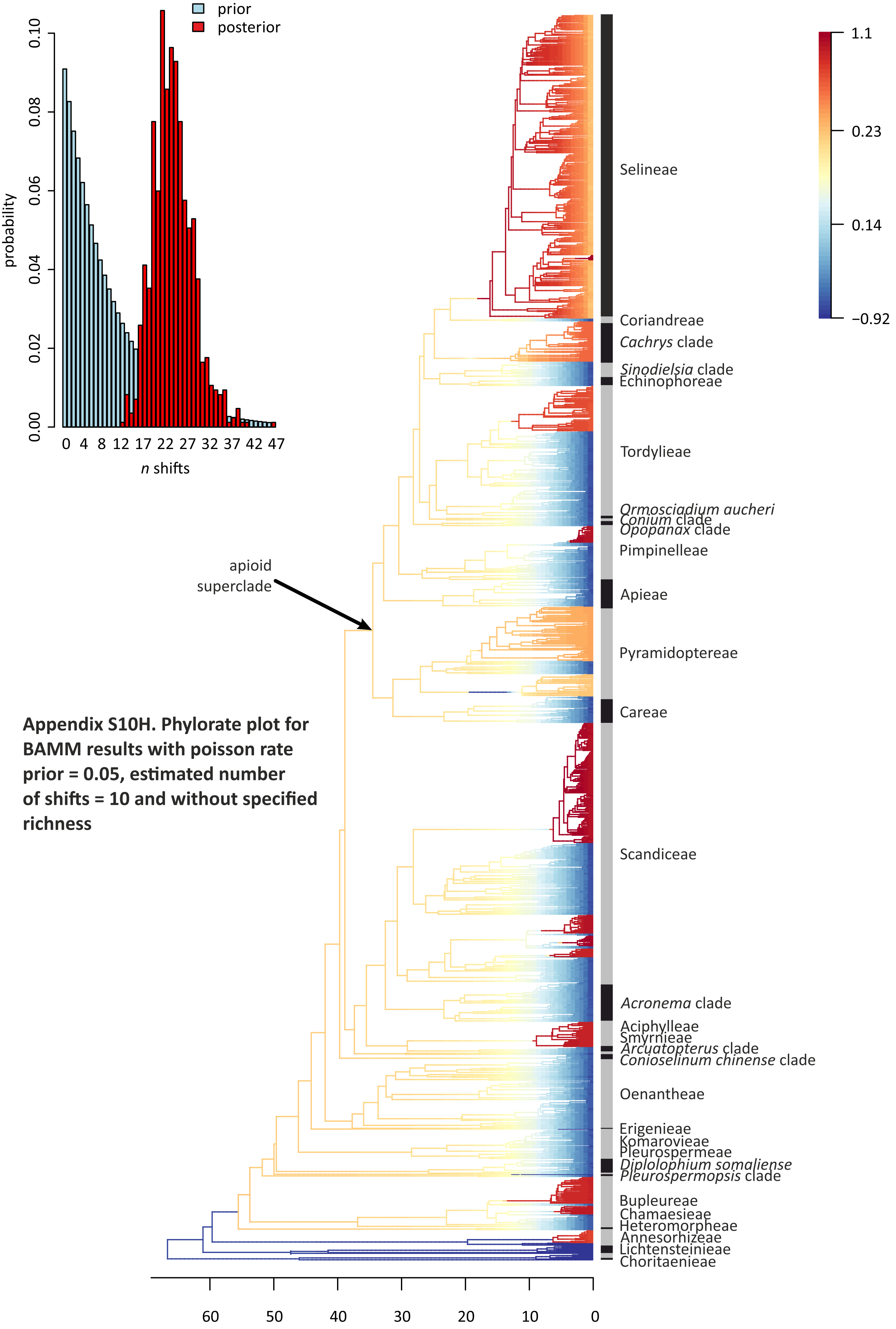


**Appendix S10H. Phylorate plot for BAMM results with poisson rate prior = 0.05, estimated number of shifts = 10 and without specified richness**

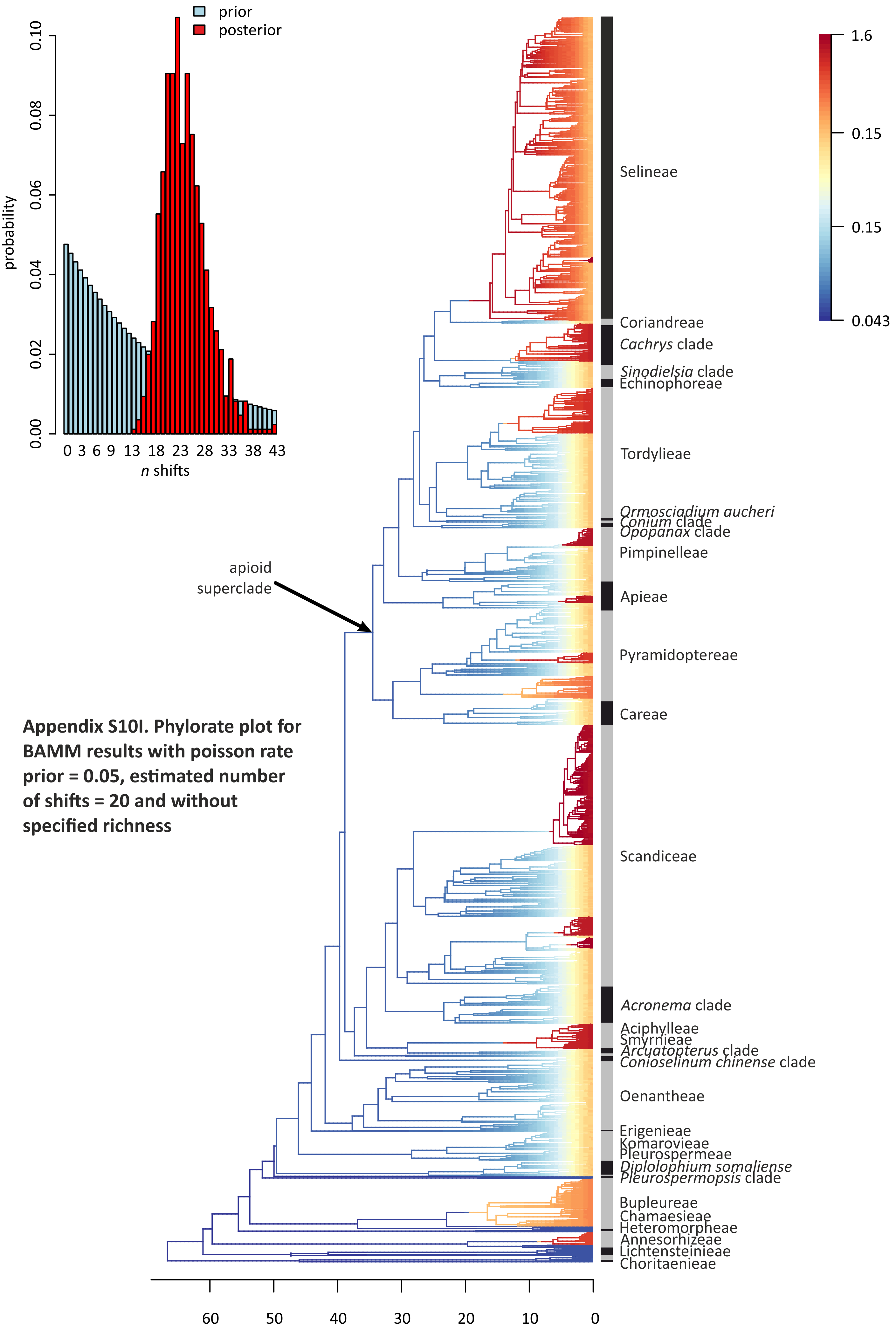


**Appendix S10I. Phylorate plot for BAMM results with poisson rate prior = 0.05, estimated number of shifts = 20 and without specified richness**

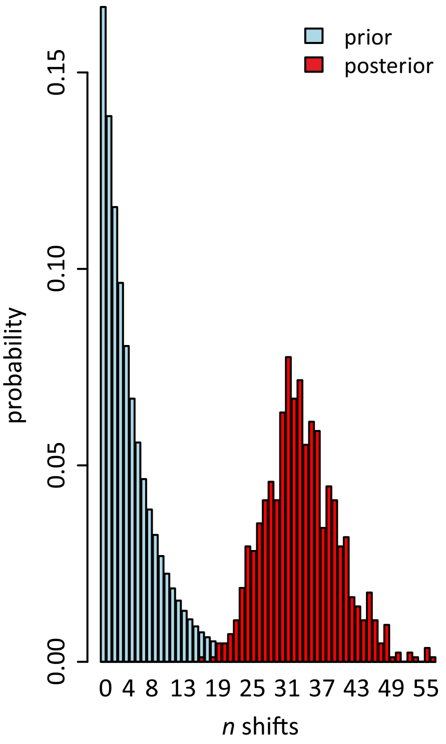


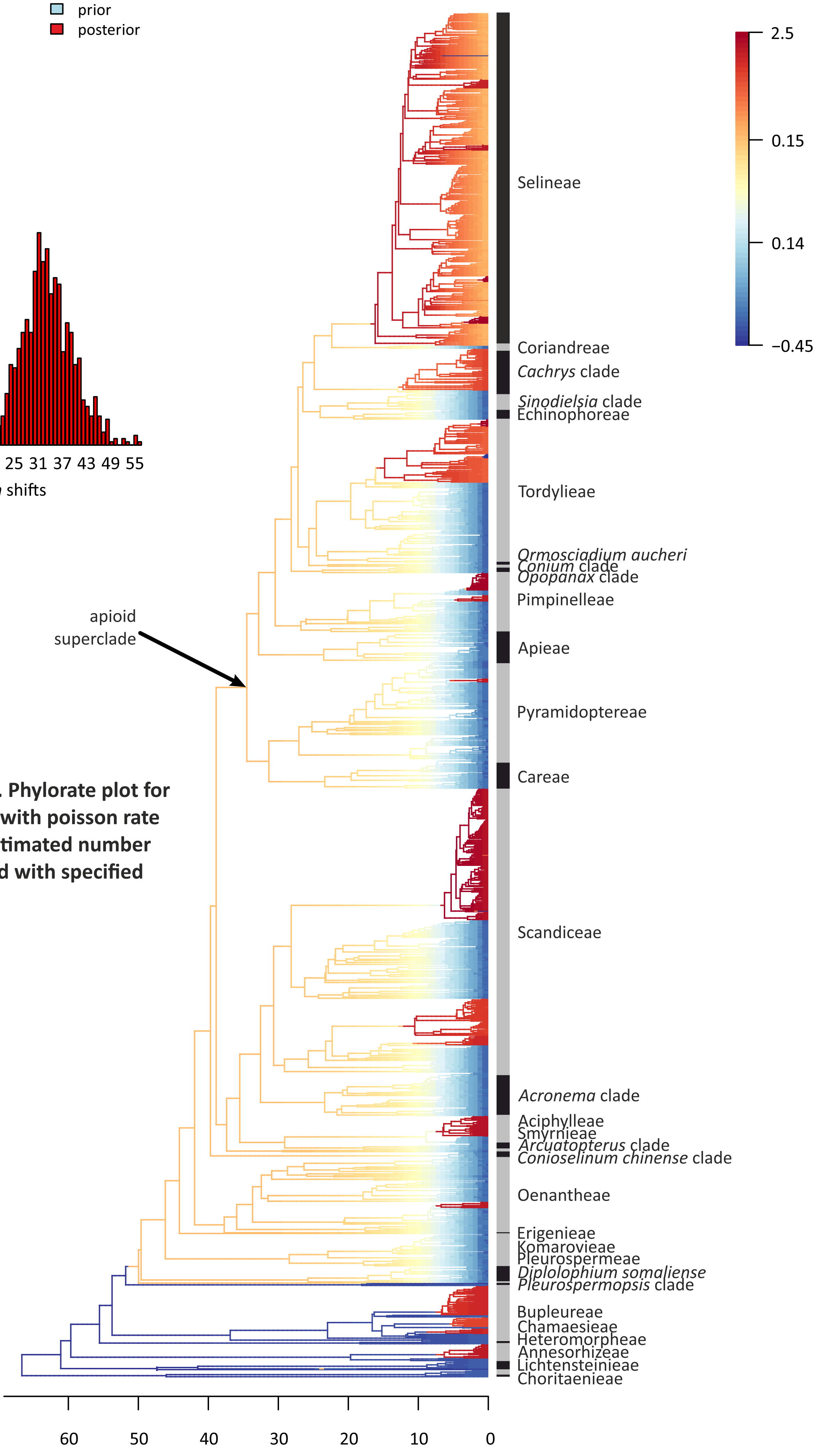


**Appendix S10J. Phylorate plot for BAMM results with poisson rate prior = 0.05, estimated number of shifts = 5 and with specified richness**

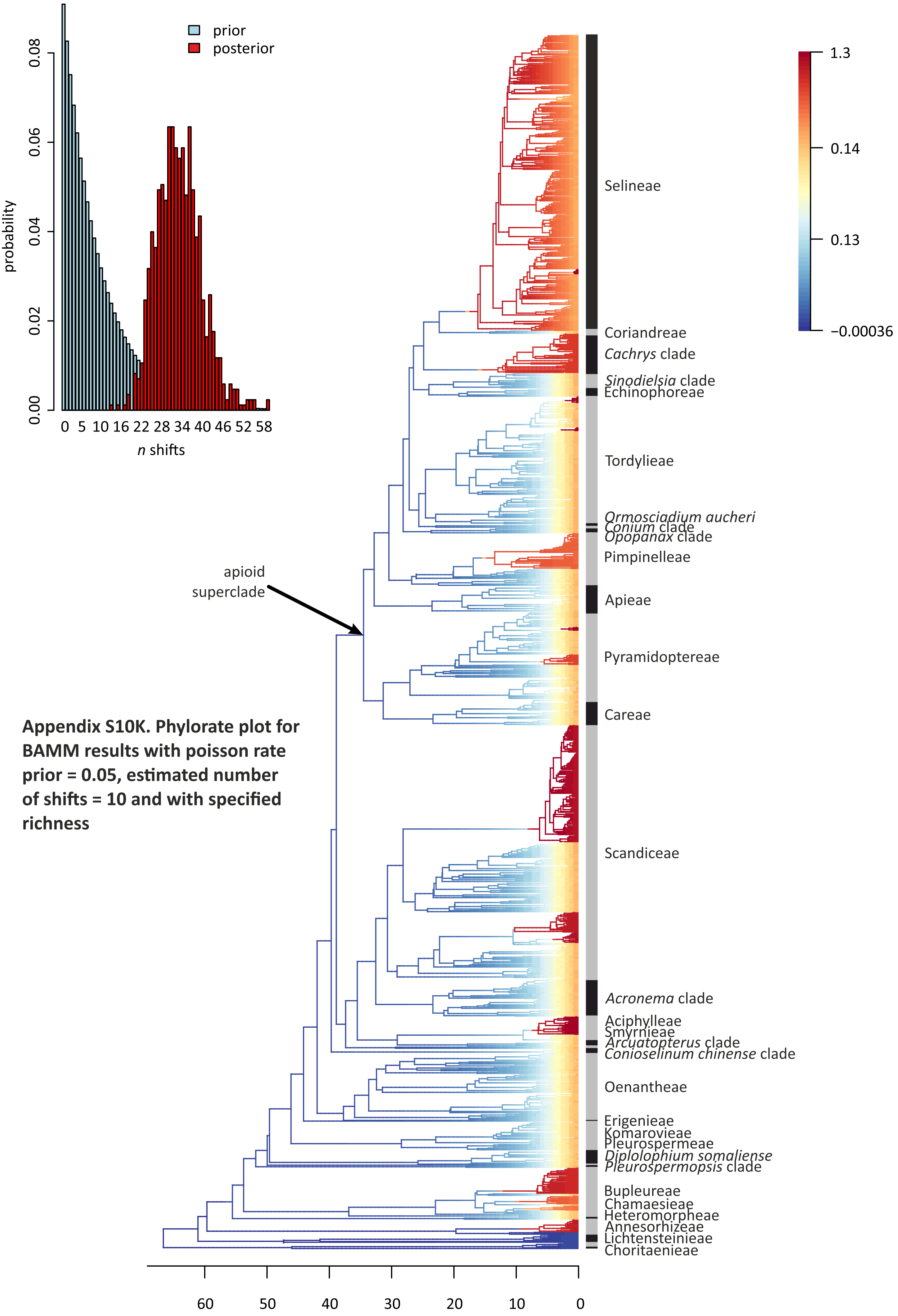


**Appendix S10K. Phylorate plot for BAMM results with poisson rate prior = 0.05, estimated number of shifts = 10 and with specified richness**

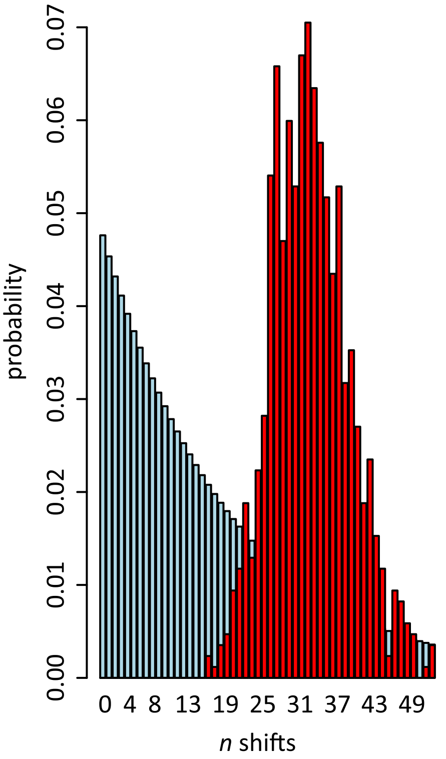


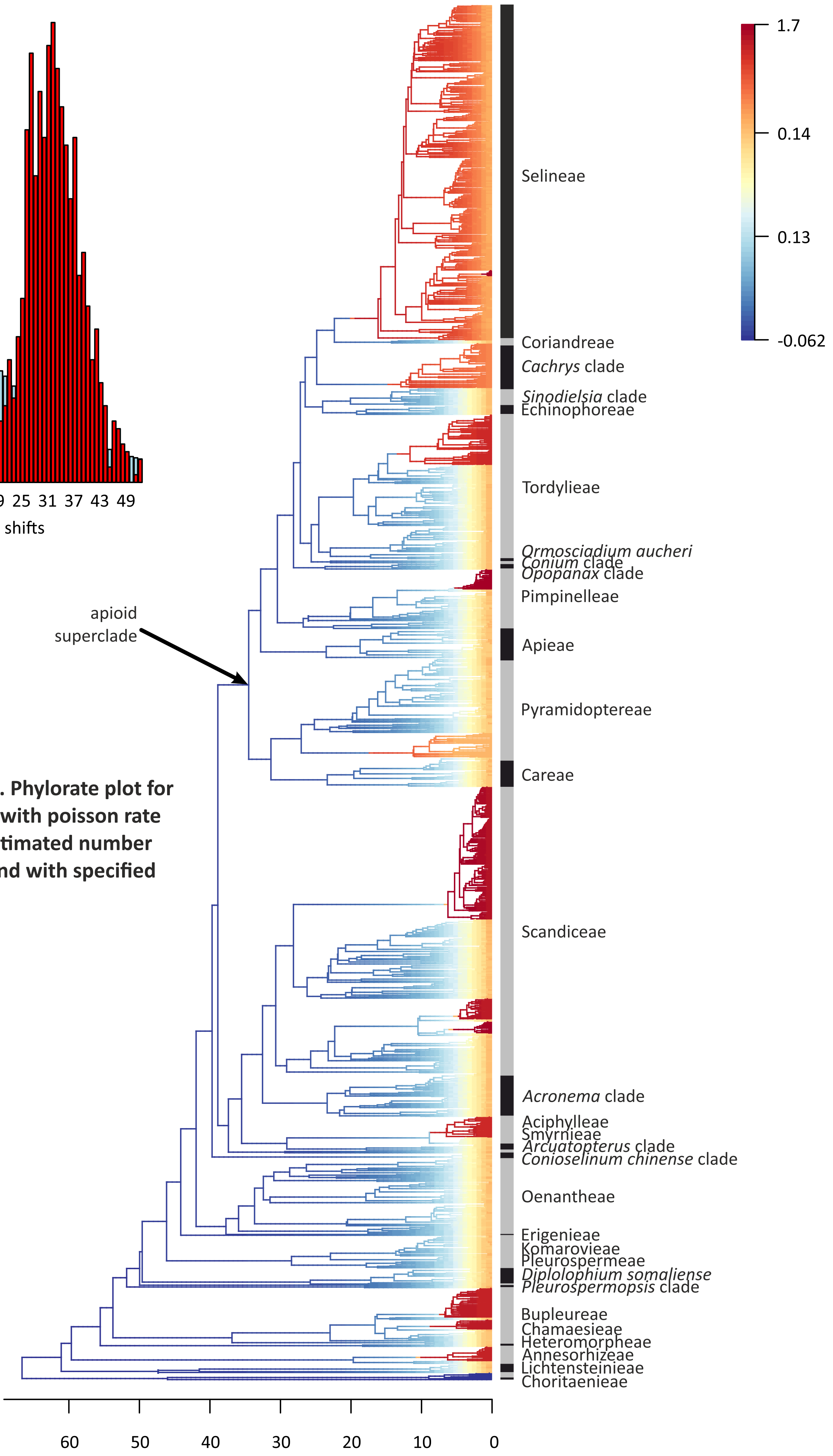


**Appendix S10L. Phylorate plot for BAMM results with poisson rate prior = 0.05, estimated number of shifts = 20 and with specified richness**

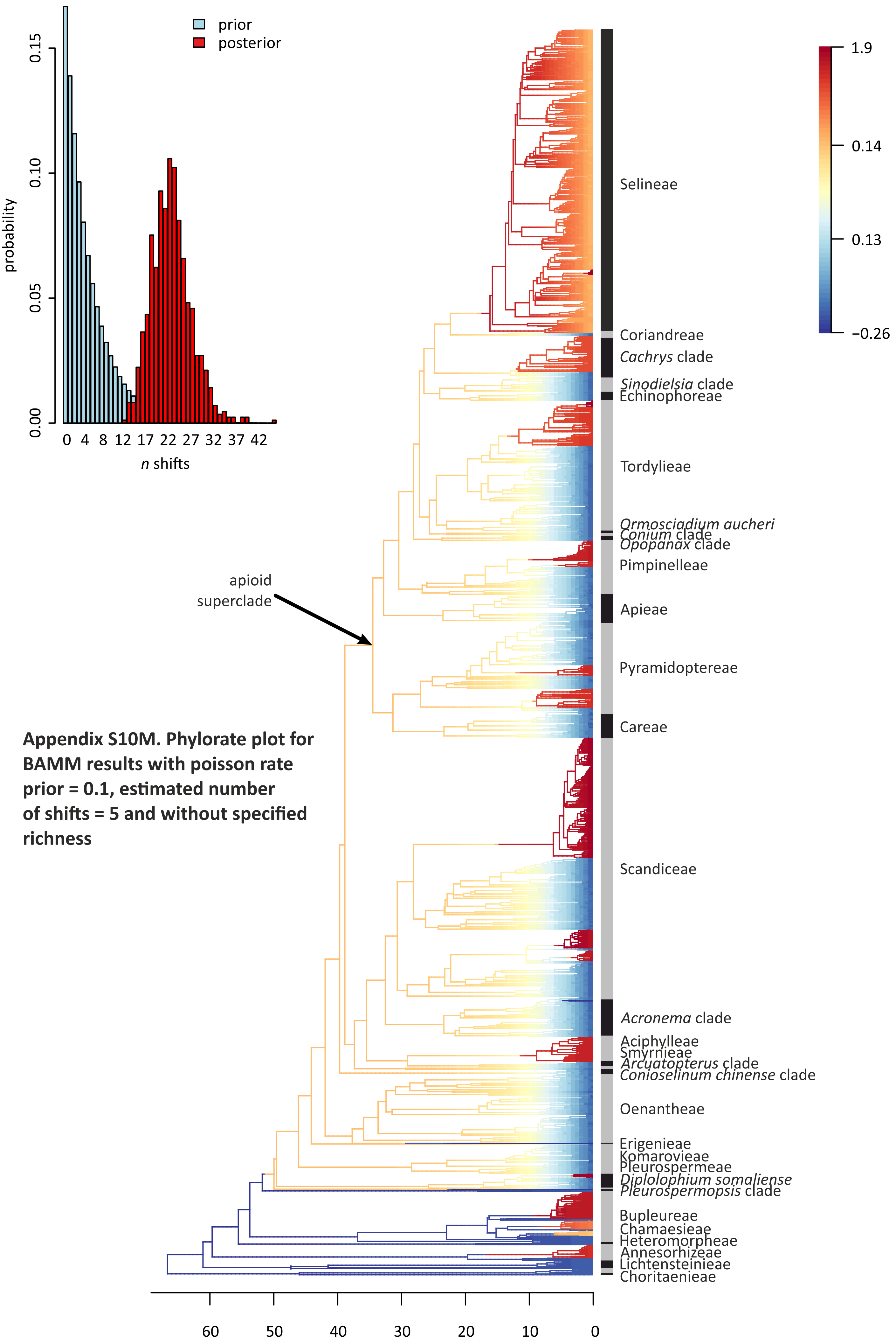


**Appendix S10M. Phylorate plot for BAMM results with poisson rate prior = 0.1, estimated number of shifts = 5 and without specified richness**

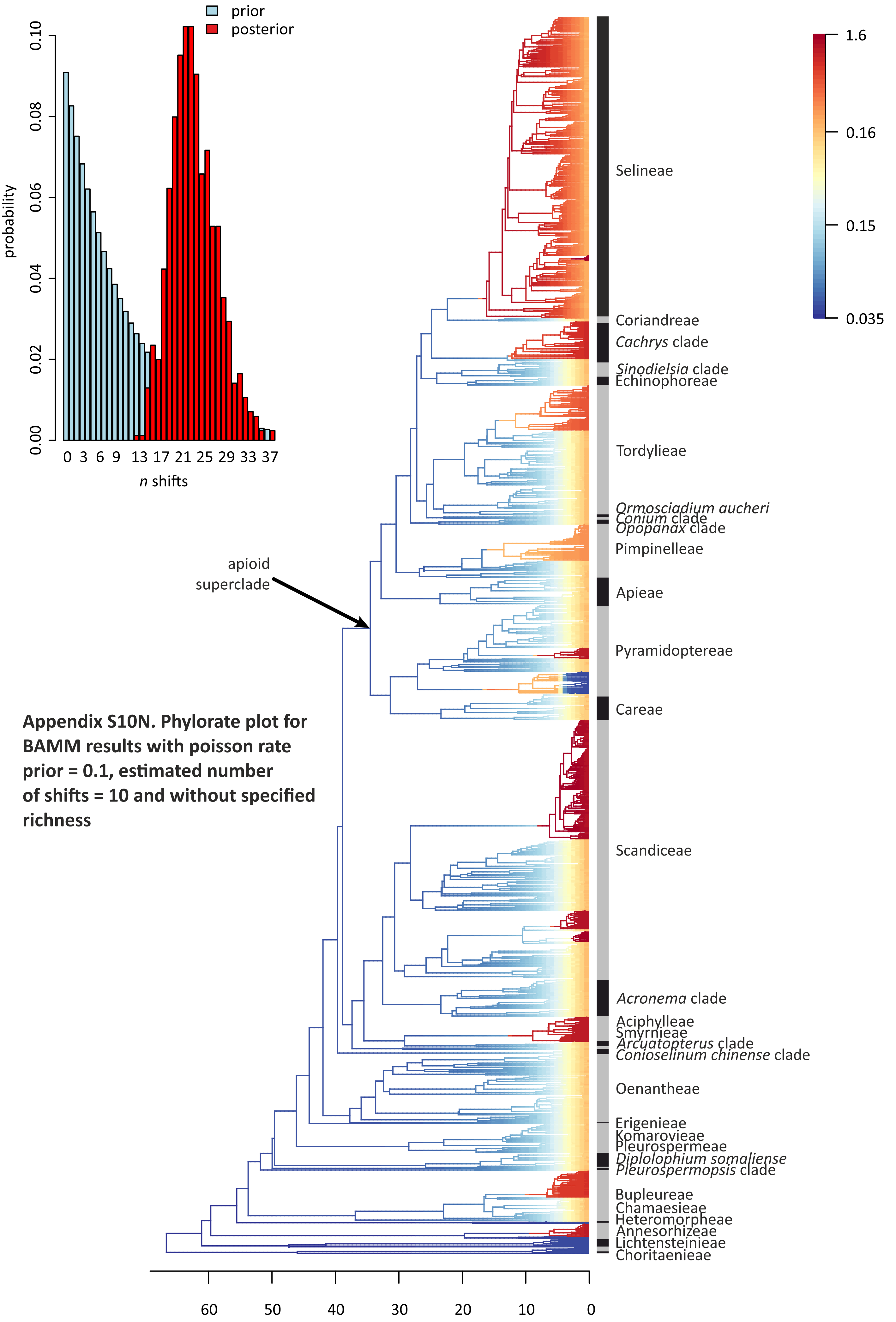


**Appendix S10N. Phylorate plot for BAMM results with poisson rate prior = 0.1, estimated number of shifts = 10 and without specified richness**

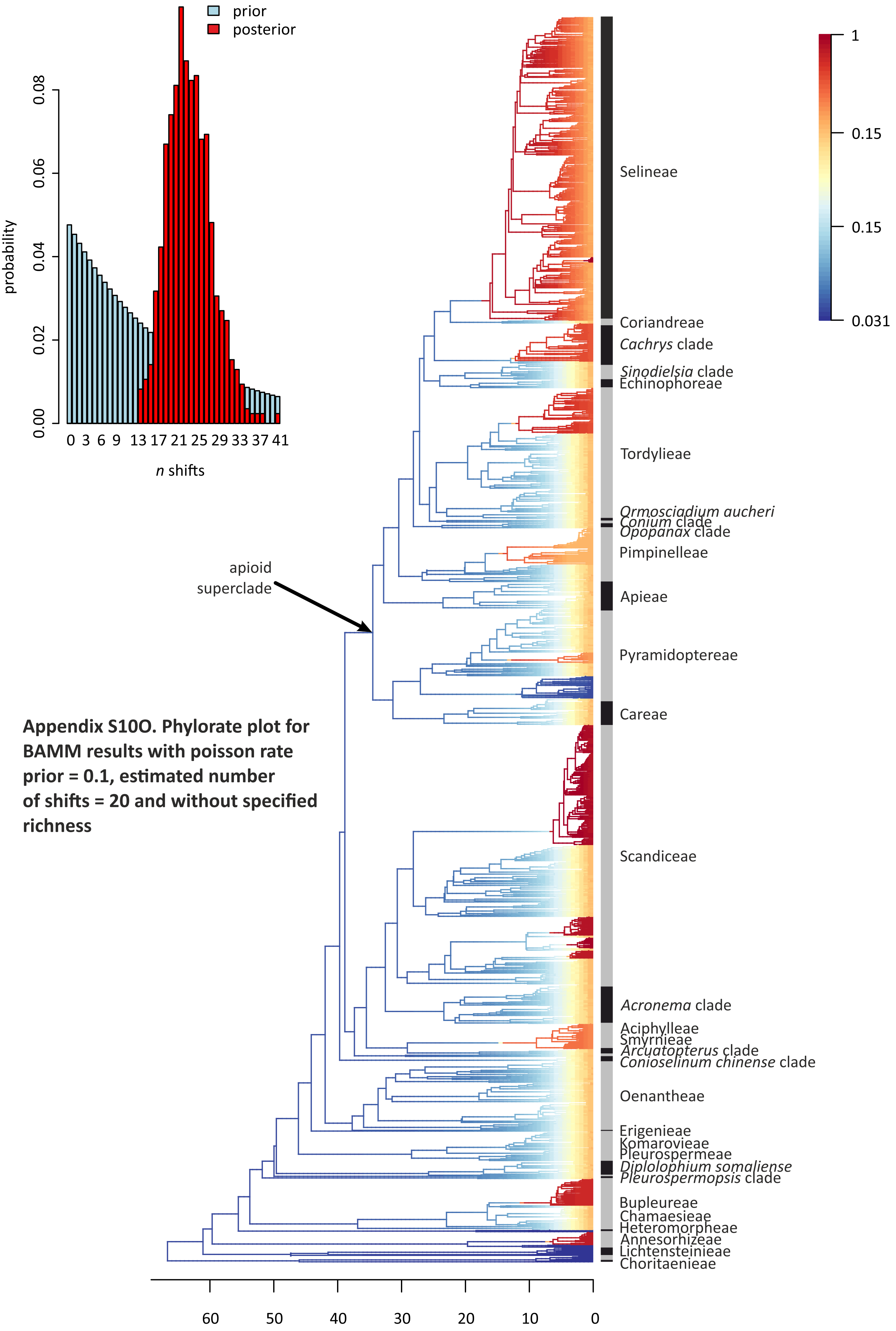


**Appendix S10O. Phylorate plot for BAMM results with poisson rate prior = 0.1, estimated number of shifts = 20 and without specified richness**

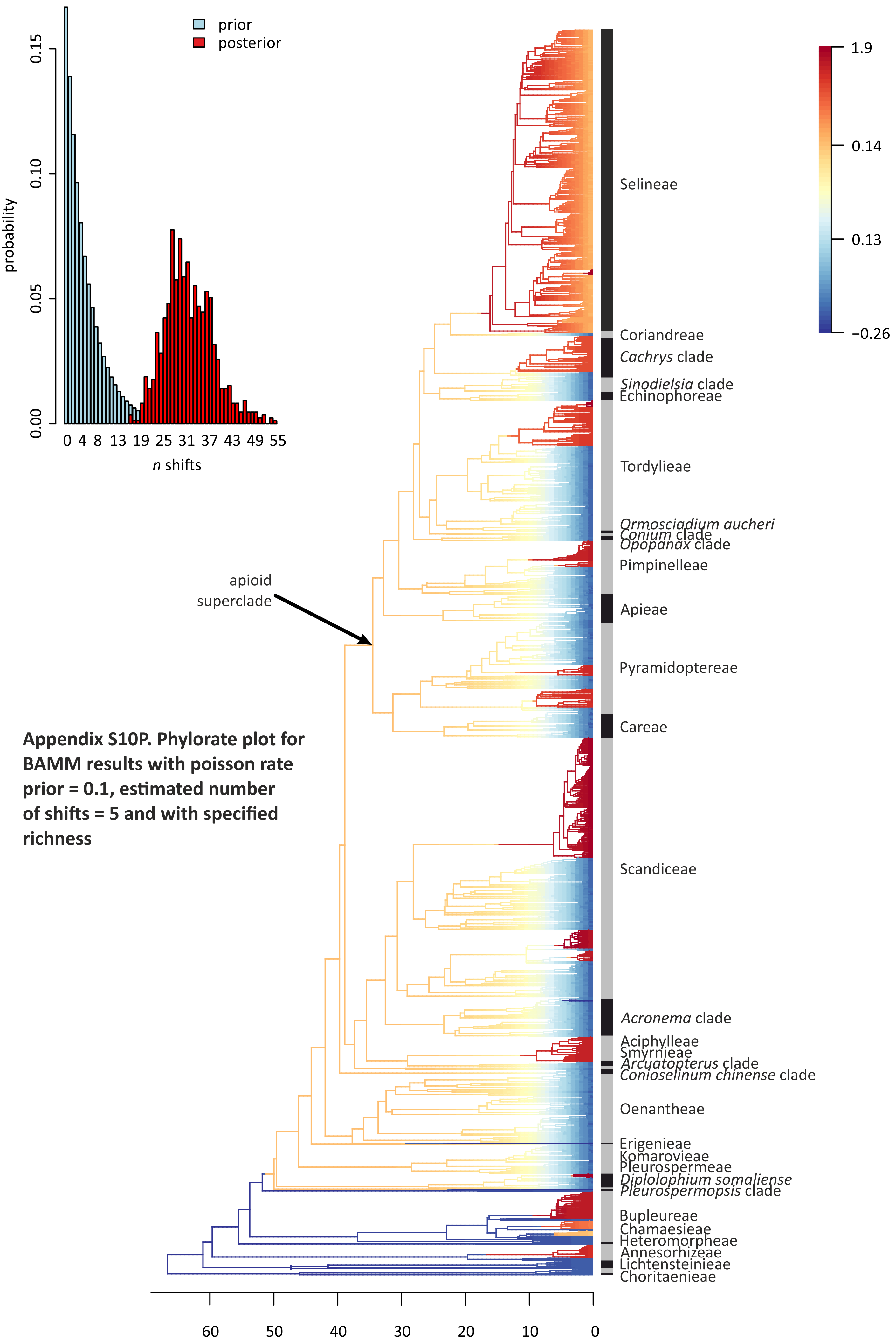


**Appendix S10P. Phylorate plot for BAMM results with poisson rate prior = 0.1, estimated number of shifts = 5 and with specified richness**

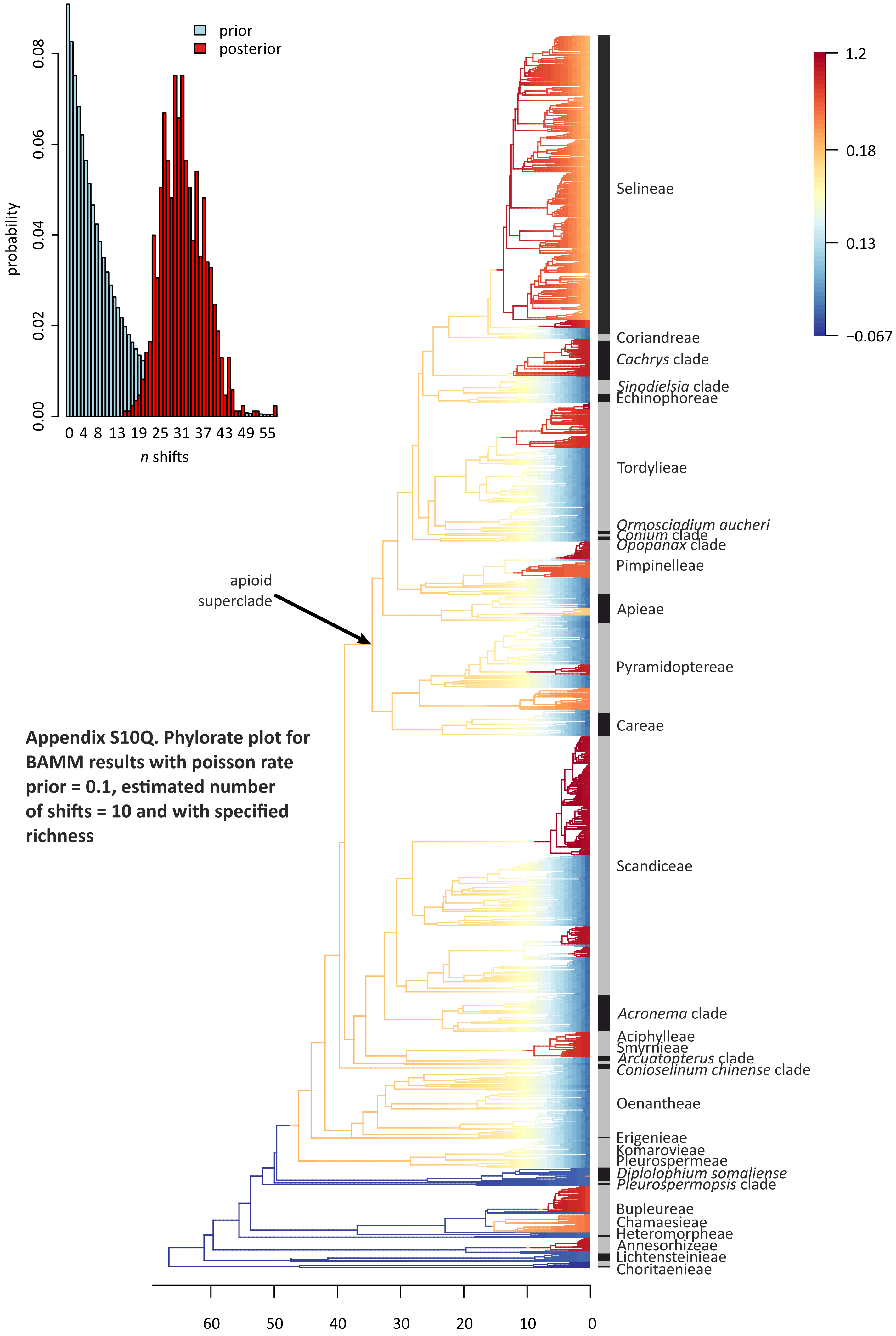


**Appendix S10Q. Phylorate plot for BAMM results with poisson rate prior = 0.1, estimated number of shifts = 10 and with specified richness**

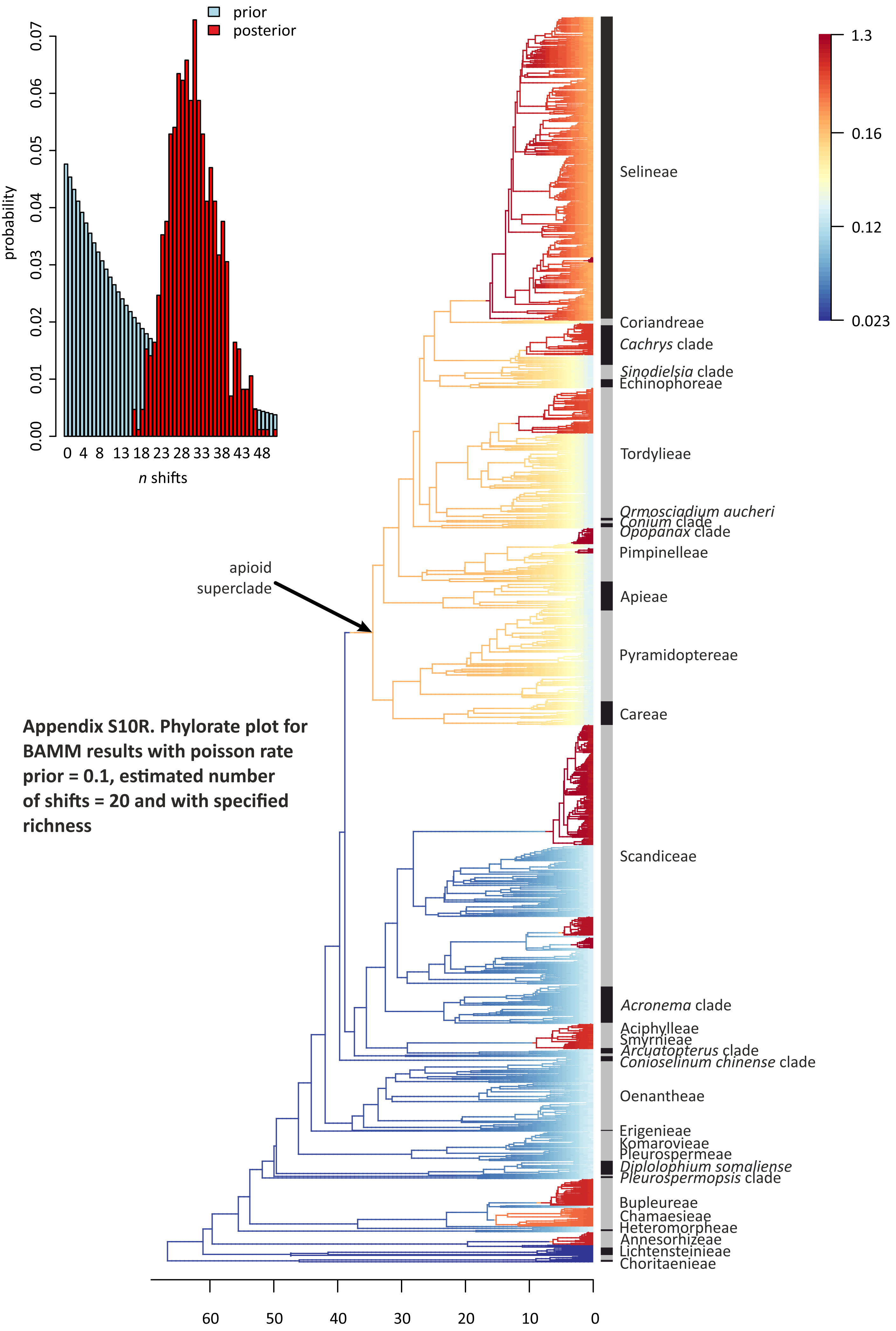


**Appendix S10R. Phylorate plot for BAMM results with poisson rate prior = 0.1, estimated number of shifts = 20 and with specified richness**

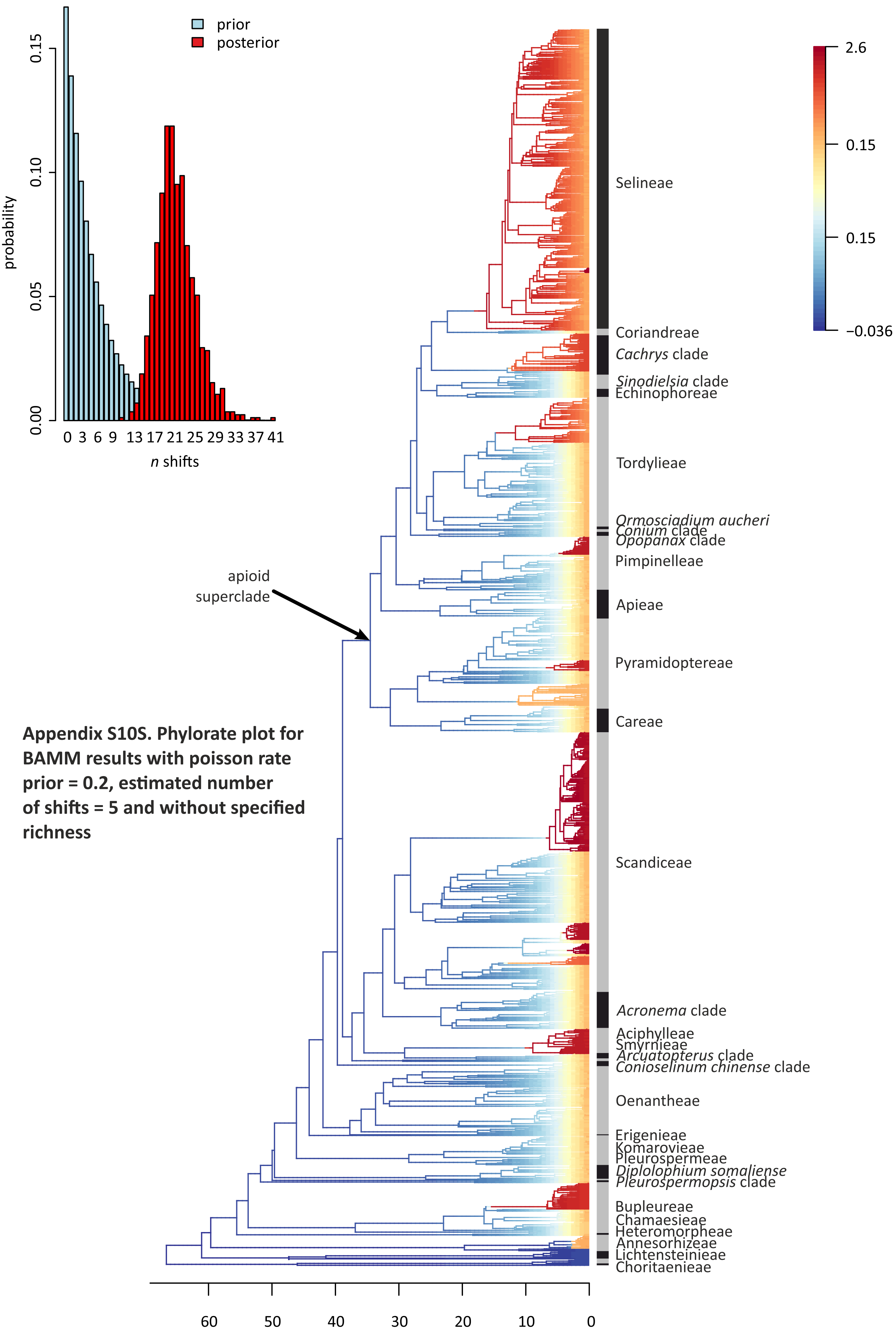


**Appendix S10S. Phylorate plot for BAMM results with poisson rate prior = 0.2, estimated number of shifts = 5 and without specified richness**

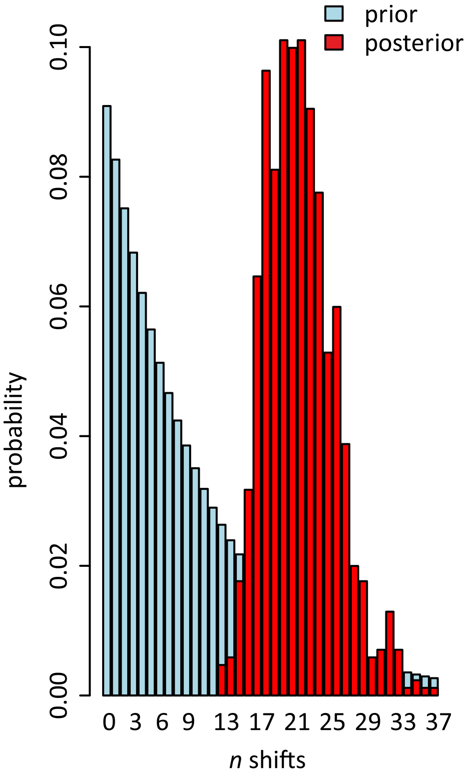


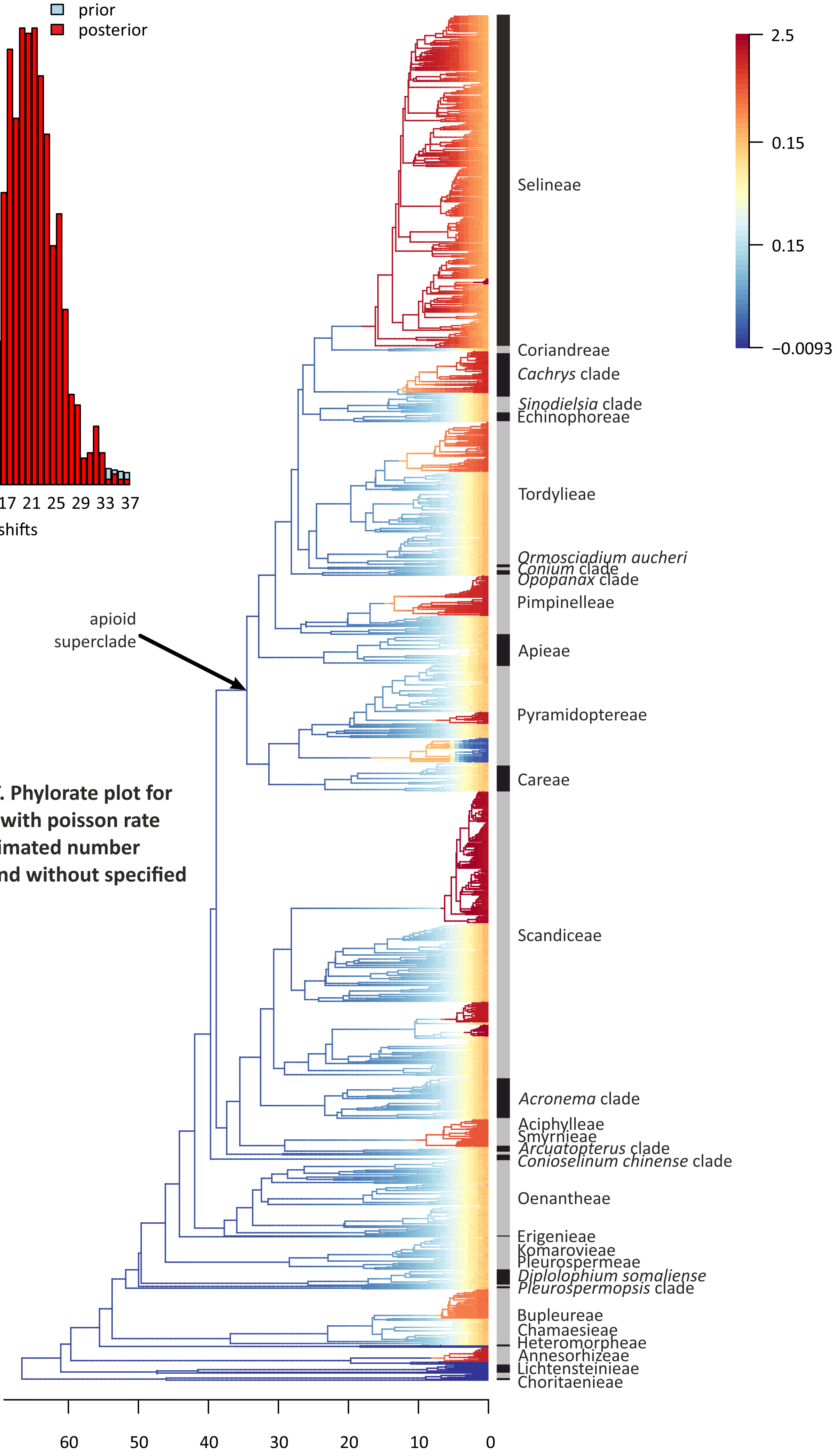


**Appendix S10T. Phylorate plot for BAMM results with poisson rate prior = 0.2, estimated number of shifts = 10 and without specified richness**

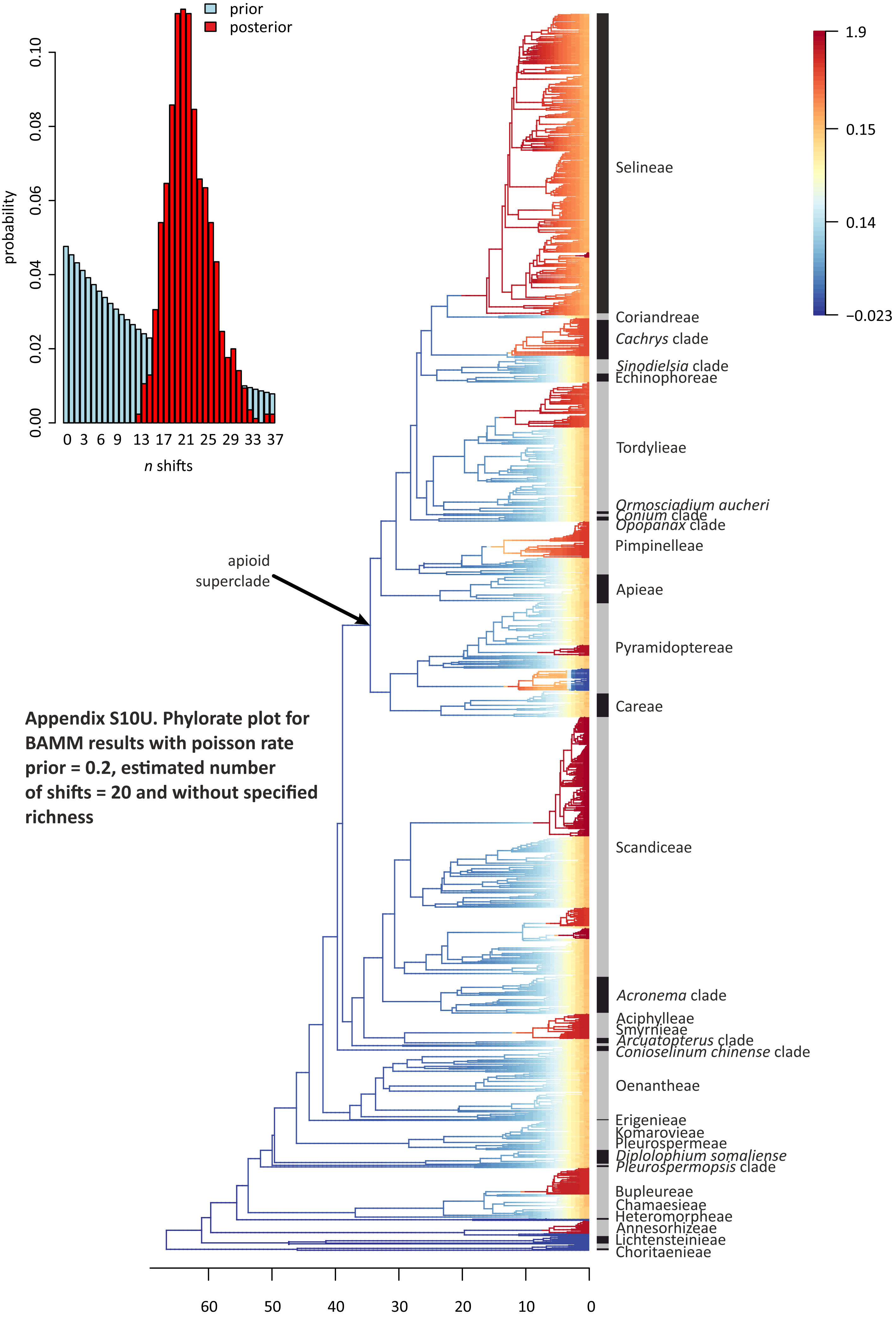


**Appendix S10U. Phylorate plot for BAMM results with poisson rate prior = 0.2, estimated number of shifts = 20 and without specified richness**

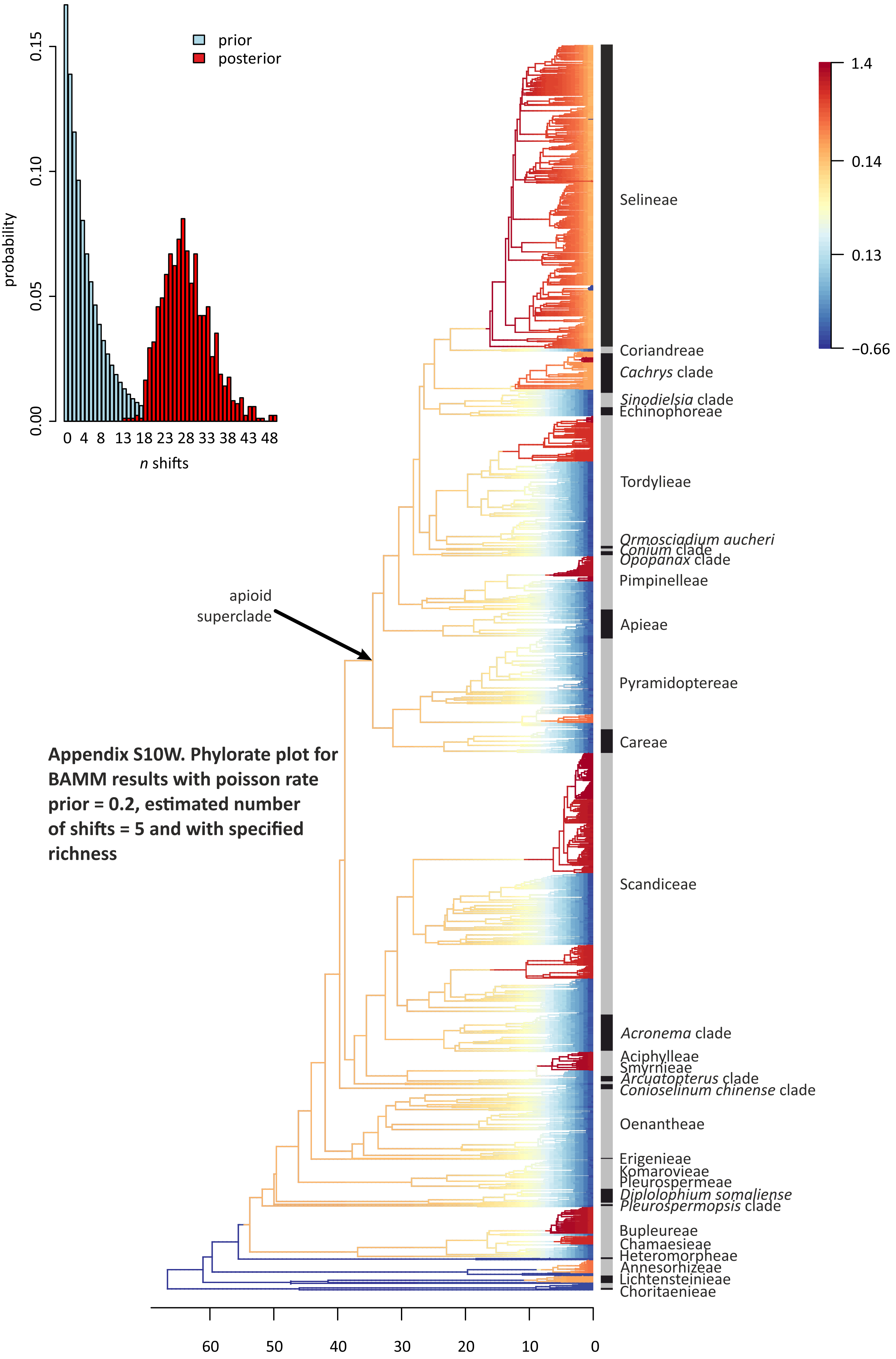


**Appendix S10W. Phylorate plot for BAMM results with poisson rate prior = 0.2, estimated number of shifts = 5 and with specified richness**

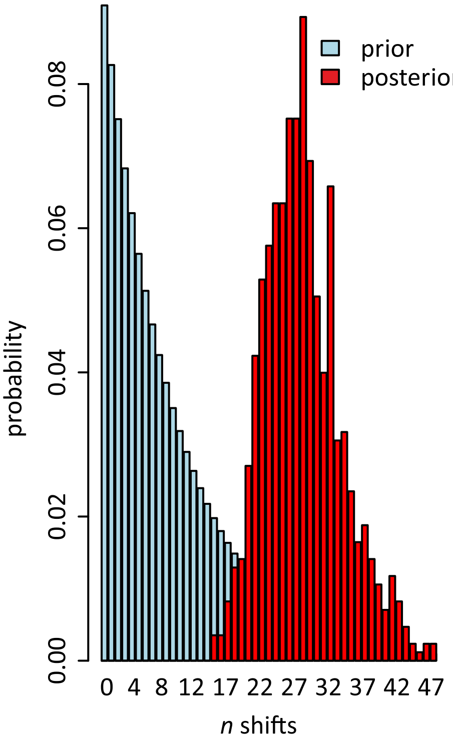


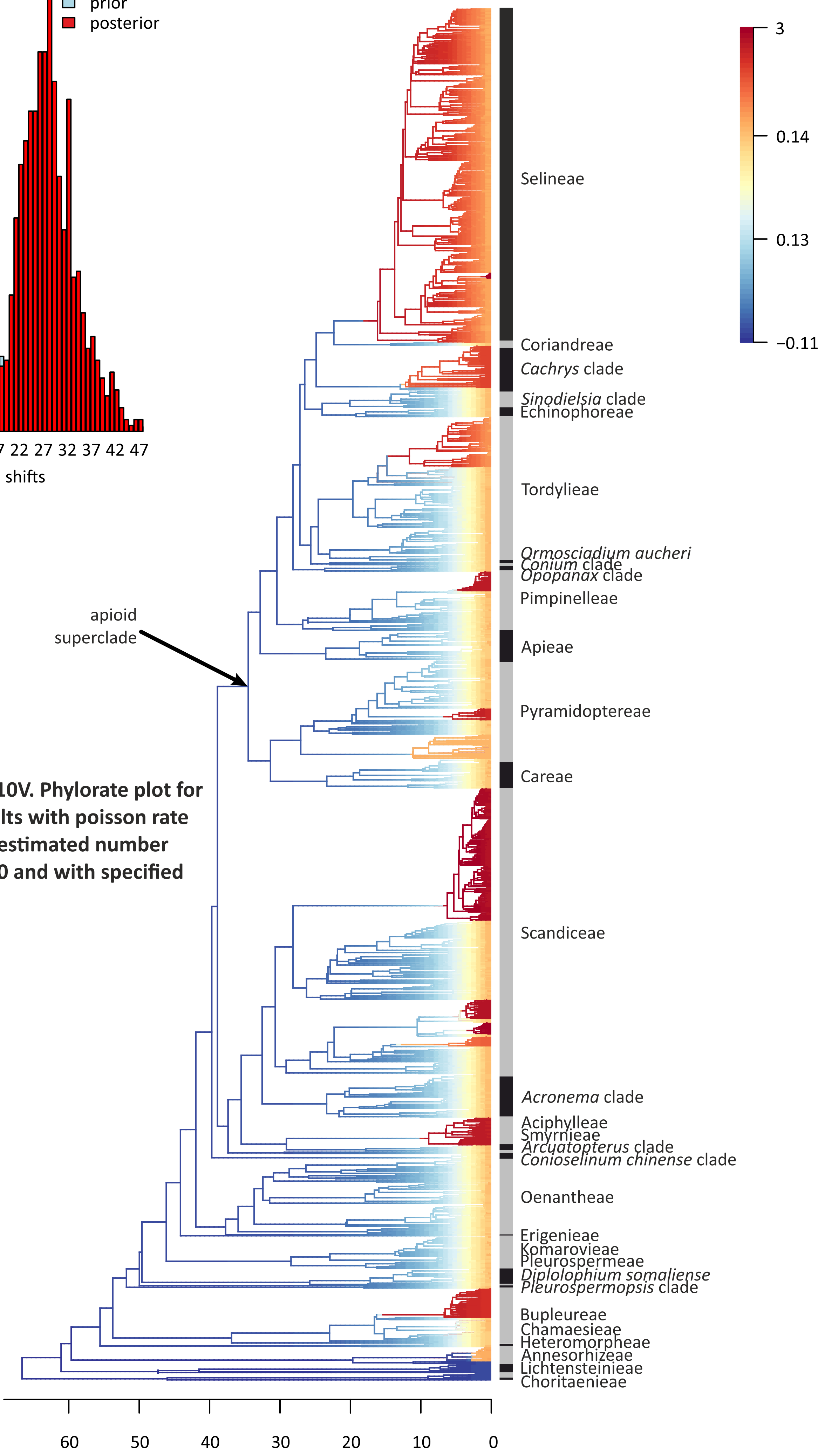


**Appendix S10V. Phylorate plot for BAMM results with poisson rate prior = 0.2, estimated number of shifts = 10 and with specified richness**

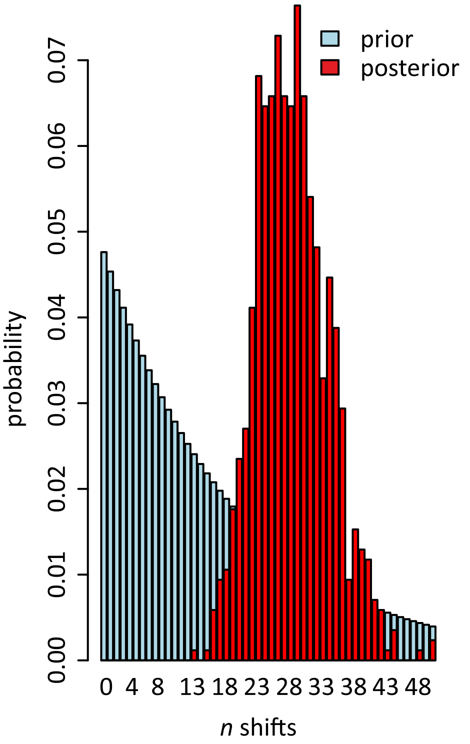


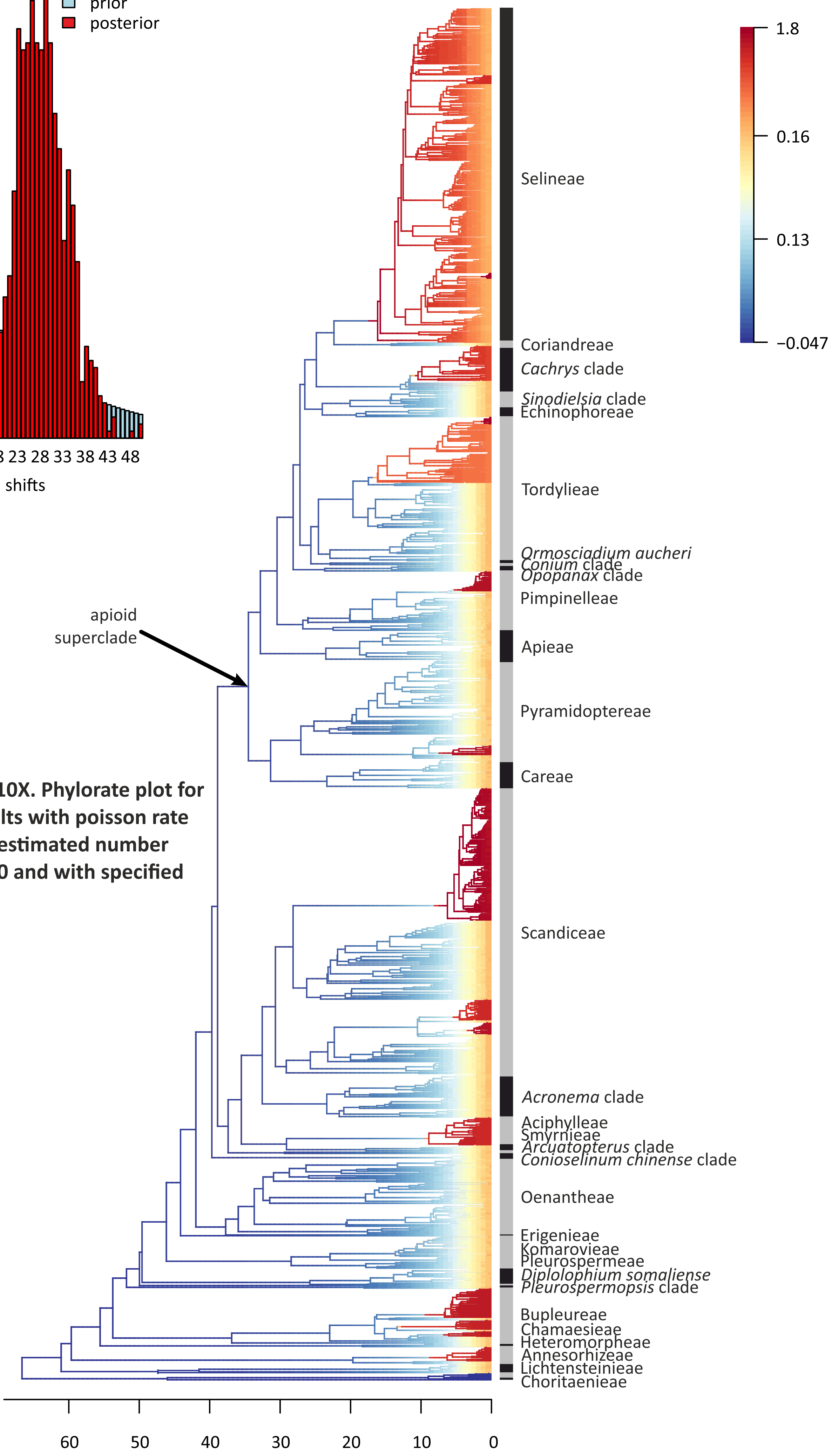


**Appendix S10X. Phylorate plot for BAMM results with poisson rate prior = 0.2, estimated number of shifts = 20 and with specified richness**
